# Supplementary material for: 2-(2-(Dimethylamino)vinyl)-4H-pyran-4-ones as Novel and Convenient Building-Blocks for the Synthesis of Conjugated 4-Pyrone Derivatives
Source: Molecules. 2022 Dec 16;27(24):8996. doi: 10.3390/molecules27248996 (PMC9788530; doi:10.3390/molecules27248996)
Supplement: Supplementary file 1 [file molecules-27-08996-s001.zip › molecules-2077145-supplementary.pdf]

Supplementary materials

2-(2-(Dimethylamino)vinyl)-4*H*-pyran-4-ones as Novel and Convenient Building-Blocks for the Synthesis of Conjugated 4-Pyrone Derivatives

Dmitrii L. Obydennov,* Diana I. Nigamatova, Alexander S. Shirinkin, Oleg E. Melnikov, Vladislav V. Fedin, Sergey A. Usachev, Alena E. Simbirtseva, Mikhail Y. Kornev, and Vyacheslav Y. Sosnovskikh

Institute of Natural Sciences and Mathematics, Ural Federal University, 51 Lenina Ave., 620000 Ekaterinburg, Russia
* Correspondence: dobydennov@mail.ru; Tel.: +7-343-3899597

Table of Contents

1. Quantum mechanical calculations details S2
2. Cartesian coordinates of the GS optimized structure for compounds **4a** and **5a** S3
3. Cartesian coordinates of the S_1 relaxed geometry for compounds **4a** and **5a** S8
4. Table S1. Ground state frontier orbitals for compounds **4a** and **5a** in vacuo S13
5. Table S2. Ground state frontier orbitals for compounds **4a** and **5a** in DMSO S14
6. Table S3. Ground state frontier orbitals for compounds **4a** and **5a** in ethanol S15
7. Table S4. Ground state frontier orbitals for compounds **4a** and **5a** in methanol S16
8. Table S5. Frontier orbitals of the first singlet excited state for the relaxed geometry of compounds **4a** and **5a** in vacuo S17
9. Table S6. Frontier orbitals of the first singlet excited state for the relaxed geometry of compounds **4a** and **5a** in DMSO S18
10. Table S7. Frontier orbitals of the first singlet excited state for the relaxed geometry of compounds **4a** and **5a** in ethanol S19
11. Table S8. Frontier orbitals of the first singlet excited state for rhe relaxed geometry of compounds **4a** and **5a** in methanol S20
12. Calculated normalized UV-vis spectra for compounds **4a** and **5a** in DMSO, methanol, and ethanol S21
13. Electron density difference maps for compounds **4a** and **5a** S24

The method of preparation of 2-methyl-6-trifluoromethyl-4-pyrone (**1c**) S28

^{1}H, ^{19}F and ^{13}C NMR spectra of compounds **2** S29

^{1}H and ^{13}C NMR spectra of compounds **4a,b** S44

^{1}H and ^{13}C NMR spectra of compounds **5a,b** S48

^{1}H and ^{13}C NMR spectra of compounds **6a–e** S52

^{1}H and ^{13}C NMR spectra of compounds **7a,b** S62

^{1}H and ^{13}C NMR spectra of compounds **8a,b** S66

^{1}H and ^{13}C NMR spectra of compound **9** S70

1. Quantum mechanical calculations details

The ground state molecular geometry of two compounds under investigation was fully optimized at density functional theory (DFT) level, both in vacuo and in the solvated phase (DMSO, MeOH, EtOH). For all geometry optimizations, the B3LYP hybrid functional[1], coupled with the 6-31G(d,p)++ basis set was chosen. Solvent effects were taken into account via the implicit conductor-like polarizable continuum model (C-PCM). For the evaluation of energetics, Solvation Model Density (SMD) parametrization was employed.[2] The vibrational frequencies and thermochemicals were computed in harmonic approximation at T = 298.15 K and p = 1 atm, and no imaginary frequencies were found.

The UV-vis absorption spectra for the equilibrium geometries were calculated at time dependent density functional theory (TD-DFT) level, accounting for $S_0 \rightarrow S_n$ (n = 1 to 7). The nature of the vertical excited electronic state was analyzed both in vacuo and in the solvated phase.

The first singlet excited state $S(\pi,\pi^*)$ state geometry was optimized using analytical gradients and the first transitions $S_1 \rightarrow S_0$ of the emission. Properties of the excited states were calculated using the long-range corrected functional CAM-B3LYP[3] coupled with the 6−31G(d,p)++ basis set. The non-equilibrium solvation regime was set for vertical excited states calculations in the solvent phase, whereas the equilibrium solvation was used for adiabatic ones. All calculated UV-vis spectra were plotted as gaussian curves with wavelengths of absorption/emission maxima as an expected value and $\sigma = 0.4$ eV.

The integration grid for the calculations was set to 96 radial shells and 302 angular points.
The RMS gradient convergence tolerance was set to 10^{-7} Hartree/Bohr for GS optimizations, and to 10^{-5} Hartree/Bohr for S_1 optimizations. The density matrix convergence threshold for the self-consistent field was set to 10^{-5} a.u. for all DFT and to 10^{-6} a.u. for all TD-DFT optimizations.

All calculations were performed using the US GAMESS (ver. 30 sep 2021, R2 Patch 1) software package for Linux x64.[4] Frontier MOs were plotted with MacMolPlt software (ver. 7.7).[5]

References

1. Becke, A.D. Density-functional thermochemistry. III. The role of exact exchange. J. Chem. Phys. 1993, 98, 5648–5652. doi: 10.1063/1.464913.
2. Marenich, A.V.; Cramer, C.J.; Truhlar, D.G. Universal solvation model based on solute electron density and on a continuum model of the solvent defined by the bulk dielectric constant and atomic surface tensions. J. Phys. Chem. B 2009, 113, 6378–6396. doi: 10.1021/jp810292n.
3. Yanai, T.; Tew, D.P.; Handy, N.C. A new hybrid exchange–correlation functional using the Coulomb-attenuating method (CAM-B3LYP). Chem. Phys. Lett. 2004, 393, 51–57. doi: 10.1016/j.cplett.2004.06.011.
4. Barca, G.M.J.; Bertoni, C.; Carrington, L.; Datta, D.; De Silva, N.; Deustua, J.E.; Fedorov, D.G.; Gour, J.R.; Gunina, A.O.; Guidez, E.; Harville, T.; Irle, S.; Ivanic, J.; Kowalski, K.; Leang, S.S.; Li, H.; Li, W.; Lutz, J.J.; Magoulas, I.; Mato, J.; Mironov, V.; Nakata, H.; Pham, B.Q.; Piecuch, P.; Poole, D.; Pruitt, S.R.; Rendell, A.P.; Roskop, L.B.; Ruedenberg, K.; Sattasathuchana, T.; Schmidt, M.W.; Shen, J.; Slipchenko, L.; Sosonkina, M.; Sundriyal, V.; Tiwari, A.; Vallejo, G.J.L.; Westheimer, B.; Włoch, M.; Xu, P.; Zahariev, F.; Gordon, M.S. Recent developments in the general atomic and molecular electronic structure system. J. Chem. Phys. 2020, 152, 154102. doi: 10.1063/5.0005188.
5. Bode, B.M.; Gordon, M.S. J. Mol. Graphics and Modeling 1998, 16, 133–138. doi: 10.1016/s1093-3263(99)00002-9.

2. Cartesian coordinates of the GS optimized structure for compounds 4a and 5a

Cartesian coordinates (Å) of the optimized structure for compound **4a** in vacuo

C	6.0	2.0032833288	-1.1754132562	-0.0040824306
C	6.0	0.6575621755	-1.0821830893	0.0350349529
C	6.0	2.1062277022	1.2704216221	-0.0309512678
O	8.0	0.0168863500	0.1289207861	0.0433940306
C	6.0	0.7416680288	1.2977591084	0.0093136134
C	6.0	2.8529514135	0.0222863109	-0.0405000439
H	1.0	2.6599580495	2.2024372589	-0.0574581214
O	8.0	4.0902171232	-0.0390633889	-0.0758866366
C	6.0	-0.0692269393	2.4937861373	0.0150671641
H	1.0	0.4862987396	3.4241871992	-0.0119766366
C	6.0	-1.4314764846	2.4779968539	0.0521031326
N	7.0	-2.2663283378	3.5577275181	0.1311807531
C	6.0	-3.6647951694	3.4003583439	-0.2471491451
H	1.0	-1.9451296486	1.5212124397	0.0452908605
H	1.0	-3.9876786021	2.3787849543	-0.0316380652
H	1.0	-3.8316758487	3.5990545863	-1.3174646067
H	1.0	-4.2881131161	4.0873986809	0.3339870250
C	6.0	-1.7176205991	4.9026236076	0.0759191708
H	1.0	-2.5022906453	5.6212495613	0.3238531549
H	1.0	-1.3174852599	5.1479118448	-0.9207243095
H	1.0	-0.9116260114	5.0086360028	0.8086828472
C	6.0	-0.3205339271	-2.2136110511	0.0743123771
H	1.0	2.4813090524	-2.1485749338	-0.0084167555
H	1.0	0.2042219812	-3.1708946683	0.0679242709
H	1.0	-0.9916287347	-2.1732339612	-0.7916197086
H	1.0	-0.9404386907	-2.1565135877	0.9766418945

Cartesian coordinates (Å) of the optimized structure for compound **5a** in vacuo

C	6.0	1.9801166574	-1.3464803409	-0.0358224973
C	6.0	0.6220811735	-1.2340851112	-0.0349368384
C	6.0	2.1310009547	1.0894226063	0.0418038021
O	8.0	0.0123979074	-0.0019252045	-0.0057379157
C	6.0	0.7692818899	1.1453099952	0.0358294033
C	6.0	2.8506818898	-0.1777509128	0.0005904281
H	1.0	2.7061149964	2.0082715354	0.0728222962
O	8.0	4.0902616966	-0.2554122370	-0.0016032085
C	6.0	-0.0044937784	2.3681357717	0.0646612500
H	1.0	0.5860445931	3.2740467701	0.1419329518
C	6.0	-1.3622164183	2.4153383681	-0.0032946459
N	7.0	-2.1546198094	3.5308772701	0.1016725185
C	6.0	-3.4924368069	3.4936936321	-0.4729072334
H	1.0	-1.9182051616	1.4903609359	-0.1255798151
H	1.0	-3.9042585296	2.4846198002	-0.3839746143
H	1.0	-3.4992371849	3.7752080600	-1.5384817708
H	1.0	-4.1493935790	4.1808017725	0.0699479362
C	6.0	-1.5341002602	4.8411192031	0.2055406816
H	1.0	-2.3000600438	5.5826612978	0.4451828556
H	1.0	-1.0331306078	5.1427614834	-0.7287887755
H	1.0	-0.7934761138	4.8404567961	1.0110514954
H	1.0	2.4364403283	-2.3299191292	-0.0579334383
C	6.0	-0.2966760420	-2.3521028411	-0.0504167991
H	1.0	0.1758668883	-3.3271063550	-0.0809579883
C	6.0	-1.6511926089	-2.2275614424	-0.0237206075
N	7.0	-2.5743067159	-3.2392434725	-0.1079062493
H	1.0	-2.0889494992	-1.2359306339	0.0418115743

C	6.0	-2.1224723204	-4.6206157652	-0.1173851442
C	6.0	-3.9190002927	-2.9998582933	0.3990423980
H	1.0	-4.1937396056	-1.9558833806	0.2262271075
H	1.0	-4.0043484594	-3.2061791884	1.4781882590
H	1.0	-4.6345907173	-3.6351756233	-0.1324026818
H	1.0	-1.3581077075	-4.7583929596	-0.8880317008
H	1.0	-2.9664531006	-5.2739253435	-0.3519655552
H	1.0	-1.6986120535	-4.9282010801	0.8526096805

Cartesian coordinates (Å) of the optimized structure for compound **4a** in DMSO

C	6.0	2.0031902580	-1.1722161931	0.0009063016
C	6.0	0.6541268227	-1.0806273306	0.0241617403
C	6.0	2.1142853094	1.2667484707	-0.0164895426
O	8.0	0.0219258834	0.1316088129	0.0299326128
C	6.0	0.7391355202	1.3027754276	0.0066534212
C	6.0	2.8453178978	0.0241382304	-0.0206366100
H	1.0	2.6646591770	2.2021148037	-0.0345108952
O	8.0	4.0970653122	-0.0455151315	-0.0418728446
C	6.0	-0.0605167010	2.4938950662	0.0062718923
H	1.0	0.4940355918	3.4253621626	-0.0106223254
C	6.0	-1.4347231566	2.4794708853	0.0177049086
N	7.0	-2.2528185554	3.5512491005	0.0625382785
C	6.0	-3.6919800666	3.3973475114	-0.1283876141
H	1.0	-1.9492015530	1.5231383083	0.0053247959
H	1.0	-3.9804719316	2.3561762597	0.0349356577
H	1.0	-3.9957191036	3.6917338342	-1.1424884367
H	1.0	-4.2355574521	4.0188549057	0.5912278955
C	6.0	-1.7117907925	4.9042370172	0.0324157048
H	1.0	-2.5118867866	5.6177149146	0.2389462086
H	1.0	-1.2763635463	5.1440480931	-0.9479150764
H	1.0	-0.9390200892	5.0259814394	0.7993610575
C	6.0	-0.3216106357	-2.2105681266	0.0465291899
H	1.0	2.4746578358	-2.1493877564	-0.0034949777
H	1.0	0.2008459312	-3.1695865238	0.0446438328
H	1.0	-0.9815507656	-2.1691745716	-0.8283867407
H	1.0	-0.9559276277	-2.1586493651	0.9396806611

Cartesian coordinates (Å) of the optimized structure for compound **5a** in DMSO

C	6.0	1.9898183327	-1.3444727799	0.0543058463
C	6.0	0.6233819448	-1.2352286252	0.0289082391
C	6.0	2.1474179748	1.0847303301	-0.0188019552
O	8.0	0.0251562302	0.0012567256	-0.0045391144
C	6.0	0.7770087074	1.1519996362	-0.0291958651
C	6.0	2.8500851244	-0.1798910967	0.0252151794
H	1.0	2.7211252666	2.0059232939	-0.0372046827
O	8.0	4.1056598374	-0.2629544494	0.0358311723
C	6.0	0.0140401788	2.3701710376	-0.0613201861
H	1.0	0.6028723709	3.2781491618	-0.1309983206
C	6.0	-1.3539858796	2.4175599187	0.0290616677
N	7.0	-2.1232224535	3.5282716742	0.0738908895
C	6.0	-3.5755236447	3.4356552058	-0.0381765007
H	1.0	-1.9102671933	1.4877100709	0.0911229421
H	1.0	-3.9002210207	2.4103641339	0.1557276123
H	1.0	-3.9192519447	3.7276472827	-1.0402143666
H	1.0	-4.0533744210	4.0913718040	0.6979462361
C	6.0	-1.5245273363	4.8508118350	-0.0492114479
H	1.0	-2.2819616808	5.6102327071	0.1550245256
H	1.0	-1.1232915484	5.0223107000	-1.0584685559
H	1.0	-0.7125226205	4.9747269605	0.6759685029
H	1.0	2.4392041283	-2.3320826102	0.0790612340

C	6.0	-0.2887514036	-2.3476192049	0.0306859755
H	1.0	0.1801306806	-3.3246365410	0.0663209987
C	6.0	-1.6526910853	-2.2224047457	-0.0328091444
N	7.0	-2.5547692208	-3.2289981527	-0.1125084213
H	1.0	-2.0924141597	-1.2297130967	-0.0383771839
C	6.0	-2.1202169339	-4.6185548696	-0.0595514994
C	6.0	-3.9717485398	-2.9667218122	0.1212311619
H	1.0	-4.1905628509	-1.9095256992	-0.0509752520
H	1.0	-4.2636571794	-3.2177558520	1.1510406340
H	1.0	-4.5825444576	-3.5596605319	-0.5671324862
H	1.0	-1.3298315734	-4.7999511227	-0.7955864939
H	1.0	-2.9635069425	-5.2699339475	-0.2990287250
H	1.0	-1.7426335230	-4.8911526588	0.9371539408

Cartesian coordinates (Å) of the optimized structure for compound **4a** in ethanol

C	6.0	1.9998423545	-1.1704607774	-0.0081791002
C	6.0	0.6506027232	-1.0761958446	0.0300309698
C	6.0	2.1210513744	1.2668028631	0.0103354849
O	8.0	0.0248866481	0.1386705678	0.0548533393
C	6.0	0.7434724401	1.3086268335	0.0394925159
C	6.0	2.8358210844	0.0236050816	-0.0294115767
H	1.0	2.6771473284	2.1985116854	-0.0021133430
O	8.0	4.0961373489	-0.0524691247	-0.0918139796
C	6.0	-0.0533358234	2.4986321121	0.0430686465
H	1.0	0.4997285932	3.4310042984	0.0360392161
C	6.0	-1.4286784193	2.4782766187	0.0277384575
N	7.0	-2.2524586080	3.5452276780	0.0587460076
C	6.0	-3.6855989095	3.3820423075	-0.1747653225
H	1.0	-1.9376178667	1.5188646662	0.0025258955
H	1.0	-3.9735532770	2.3406000357	-0.0126135162
H	1.0	-3.9597077993	3.6674919835	-1.1998464093
H	1.0	-4.2535990279	4.0055555687	0.5235911959
C	6.0	-1.7198396805	4.9027455642	0.0317110511
H	1.0	-2.5257970049	5.6107304030	0.2349082580
H	1.0	-1.2817736259	5.1462629702	-0.9464077634
H	1.0	-0.9530966586	5.0298098414	0.8033867569
C	6.0	-0.3300405742	-2.2006414193	0.0473472485
H	1.0	2.4699043254	-2.1475193776	-0.0320665782
H	1.0	0.1867298387	-3.1623543148	0.0194584991
H	1.0	-1.0026355732	-2.1368599443	-0.8164899176
H	1.0	-0.9490373895	-2.1598393867	0.9517391041

Cartesian coordinates (Å) of the optimized structure for compound **5a** in ethanol

C	6.0	1.9842724216	-1.3426787115	-0.0063520238
C	6.0	0.6160429605	-1.2315086898	0.0097008679
C	6.0	2.1491444453	1.0841700620	0.0428948971
O	8.0	0.0228581152	0.0074240249	0.0328852546
C	6.0	0.7773108356	1.1566590030	0.0490719814
C	6.0	2.8365638995	-0.1813534069	-0.0074533414
H	1.0	2.7279644250	2.0019132293	0.0538112706
O	8.0	4.1018557051	-0.2692758249	-0.0626901018
C	6.0	0.0197176262	2.3770333706	0.0616114288
H	1.0	0.6120652168	3.2848166822	0.0903220590
C	6.0	-1.3503886341	2.4229278857	0.0151109490
N	7.0	-2.1312340745	3.5270898688	0.0705832396
C	6.0	-3.5450397770	3.4373080042	-0.2901727491
H	1.0	-1.9046703516	1.4924723546	-0.0542562000
H	1.0	-3.9069196531	2.4176629095	-0.1349549603
H	1.0	-3.7105318734	3.7123428699	-1.3415034665

H	1.0	-4.1353600124	4.1080814609	0.3420177472
C	6.0	-1.5342511728	4.8570713372	0.0765327300
H	1.0	-2.3062850074	5.5974002195	0.2967283145
H	1.0	-1.0821533541	5.1052246250	-0.8948816591
H	1.0	-0.7651179774	4.9282123743	0.8524417823
H	1.0	2.4309490097	-2.3307856461	-0.0347963093
C	6.0	-0.2922521250	-2.3437139447	0.0052458998
H	1.0	0.1805960280	-3.3193145695	0.0052363981
C	6.0	-1.6584071481	-2.2237085914	-0.0168265451
N	7.0	-2.5566163940	-3.2339691619	-0.0924244666
H	1.0	-2.1042004164	-1.2343111271	0.0134671845
C	6.0	-2.1136603968	-4.6228932133	-0.0613917849
C	6.0	-3.9661403279	-2.9834000971	0.1985790216
H	1.0	-4.2009724696	-1.9284237741	0.0337144675
H	1.0	-4.2156192349	-3.2360076414	1.2391023124
H	1.0	-4.5983567005	-3.5814917217	-0.4652860380
H	1.0	-1.3354529673	-4.7935748484	-0.8123306212
H	1.0	-2.9575387792	-5.2758140740	-0.2948127482
H	1.0	-1.7197362374	-4.9040624135	0.9264994253

Cartesian coordinates (Å) of the optimized structure for compound **4a** in methanol

C	6.0	2.0000929414	-1.1705113088	-0.0026779247
C	6.0	0.6503498696	-1.0757679552	0.0243738104
C	6.0	2.1217179167	1.2665822187	-0.0057836954
O	8.0	0.0250087754	0.1392290303	0.0362603519
C	6.0	0.7433262150	1.3092879128	0.0181337403
C	6.0	2.8346036164	0.0234023724	-0.0228864163
H	1.0	2.6789602840	2.1977046949	-0.0172387401
O	8.0	4.0973843869	-0.0524974480	-0.0567497553
C	6.0	-0.0528631861	2.4986997244	0.0200248515
H	1.0	0.4997567729	3.4312247752	0.0044667322
C	6.0	-1.4286775721	2.4774957191	0.0249245071
N	7.0	-2.2511505612	3.5444117826	0.0634287738
C	6.0	-3.6890131210	3.3821783470	-0.1399192977
H	1.0	-1.9373744986	1.5177448323	0.0118499189
H	1.0	-3.9729391407	2.3391280509	0.0186133583
H	1.0	-3.9849065056	3.6769441957	-1.1560779969
H	1.0	-4.2423864396	3.9988479391	0.5762565572
C	6.0	-1.7183274910	4.9018771531	0.0243071519
H	1.0	-2.5228290991	5.6113327971	0.2278149457
H	1.0	-1.2866030600	5.1386272443	-0.9581401951
H	1.0	-0.9465141221	5.0340021579	0.7901717021
C	6.0	-0.3305514390	-2.1996344445	0.0436895900
H	1.0	2.4699969386	-2.1478673278	-0.0121931923
H	1.0	0.1857777683	-3.1617663331	0.0227844127
H	1.0	-0.9997571853	-2.1395135432	-0.8230004736
H	1.0	-0.9529739714	-2.1533287715	0.9454230681

Cartesian coordinates (Å) of the optimized structure for compound **5a** in methanol

C	6.0	1.9814883262	-1.3420839671	0.0400373673
C	6.0	0.6132189491	-1.2307886054	0.0108577525
C	6.0	2.1483723441	1.0836581617	-0.0138388485
O	8.0	0.0205446854	0.0079614174	-0.0223700777
C	6.0	0.7751907222	1.1577139628	-0.0322536389
C	6.0	2.8330591021	-0.1814305312	0.0280350996
H	1.0	2.7275306830	2.0012382643	-0.0160243064
O	8.0	4.1006086148	-0.2713291965	0.0600092628
C	6.0	0.0196669931	2.3771111332	-0.0550299587
H	1.0	0.6122209090	3.2836885217	-0.1083826926
C	6.0	-1.3502937874	2.4269552794	0.0216784161

N	7.0	-2.1169944066	3.5375764220	0.0745694219
C	6.0	-3.5698239759	3.4476294687	-0.0489561355
H	1.0	-1.9089777318	1.4978411555	0.0659720017
H	1.0	-3.8983489873	2.4223482534	0.1380976941
H	1.0	-3.9054843861	3.7454640454	-1.0518589979
H	1.0	-4.0516470468	4.1000770994	0.6873346062
C	6.0	-1.5167018074	4.8618761928	-0.0372647906
H	1.0	-2.2751012268	5.6201654263	0.1675457413
H	1.0	-1.1109978335	5.0394441350	-1.0433682503
H	1.0	-0.7094746731	4.9819823282	0.6936705401
H	1.0	2.4263822734	-2.3310332859	0.0664651329
C	6.0	-0.2932146819	-2.3440227910	0.0128450441
H	1.0	0.1811377843	-3.3184685335	0.0434402973
C	6.0	-1.6590654319	-2.2279299638	-0.0380427829
N	7.0	-2.5523234990	-3.2422397015	-0.1135976940
H	1.0	-2.1076335761	-1.2392479136	-0.0356066024
C	6.0	-2.1052997286	-4.6292125355	-0.0564398331
C	6.0	-3.9675083763	-2.9933330767	0.1510806225
H	1.0	-4.2028015076	-1.9405004890	-0.0258944478
H	1.0	-4.2331307115	-3.2385872187	1.1893027802
H	1.0	-4.5869149880	-3.5987550244	-0.5182022299
H	1.0	-1.3165041042	-4.8074905536	-0.7945431539
H	1.0	-2.9439616357	-5.2880242109	-0.2920684706
H	1.0	-1.7240239029	-4.8952667054	0.9404293872

3. Cartesian coordinates of the S_1 relaxed geometry for compounds 4a and 5a

Cartesian coordinates (Å) of the S_1 relaxed geometry for compound **4a** in vacuo

C	6.0	2.0017347376	-1.1633371813	-0.0083451048
C	6.0	0.6576075560	-1.0832466531	0.0353167234
C	6.0	2.0951593376	1.2717697196	-0.0339121942
O	8.0	-0.0005409512	0.1235959111	0.0448529186
C	6.0	0.7288854235	1.3036312245	0.0121018727
C	6.0	2.7840224206	0.0411559671	-0.0465719771
H	1.0	2.6459203455	2.2029266602	-0.0598946466
O	8.0	4.1010218243	-0.0279396513	-0.0931852806
C	6.0	-0.0748442153	2.4898601430	0.0260891241
H	1.0	0.4744018750	3.4231191950	-0.0101415006
C	6.0	-1.4301367426	2.4699311587	0.0888145250
N	7.0	-2.2651926775	3.5576615407	0.1829336935
C	6.0	-3.6233421443	3.4030914651	-0.3012920493
H	1.0	-1.9417975773	1.5136081108	0.1005853086
H	1.0	-3.9917285475	2.4078809923	-0.0437986836
H	1.0	-3.6976640288	3.5272382967	-1.3930134985
H	1.0	-4.2734429525	4.1409544109	0.1758431743
C	6.0	-1.6994600369	4.8823410916	0.0698689217
H	1.0	-2.4698392107	5.6230483625	0.2922230283
H	1.0	-1.2992440242	5.0829260786	-0.9370818810
H	1.0	-0.8896772184	5.0067944357	0.7933710741
C	6.0	-0.2973117413	-2.2224159128	0.0783083458
H	1.0	2.4879935290	-2.1294193150	-0.0145095839
H	1.0	0.2358340149	-3.1739597653	0.0701606735
H	1.0	-0.9732058552	-2.1887640849	-0.7833454508
H	1.0	-0.9152687919	-2.1710065058	0.9815296298

Cartesian coordinates (Å) of the S_1 relaxed geometry for compound **5a** in vacuo

C	6.0	2.0011917151	-1.3228726909	0.0103892830
C	6.0	0.6206863997	-1.2268137467	-0.0053472271
C	6.0	2.1516042229	1.0627645714	0.0027047089
O	8.0	-0.0100955141	0.0011456267	-0.0147248991
C	6.0	0.7697273780	1.1405117302	0.0018907812
C	6.0	2.8786429311	-0.1806123236	0.0064367843
H	1.0	2.7174572698	1.9883013100	0.0110706938
O	8.0	4.1307255904	-0.2604174425	0.0092855840
C	6.0	0.0206451203	2.3334857139	0.0187429347
H	1.0	0.5888597450	3.2551126530	0.0061555183
C	6.0	-1.3636642877	2.3456078804	0.0481468604
N	7.0	-2.1435347792	3.4563514953	0.1192680405
C	6.0	-3.5566131405	3.3374135235	-0.1728719712
H	1.0	-1.8987004915	1.4035630706	0.0524143774
H	1.0	-3.9201015476	2.3643228561	0.1642130942
H	1.0	-3.7615277325	3.4299326066	-1.2511147728
H	1.0	-4.1127433629	4.1145045255	0.3562190234
C	6.0	-1.5558414175	4.7751784884	0.0227651421
H	1.0	-2.3129367508	5.5233760430	0.2601144988
H	1.0	-1.1665182855	4.9758258703	-0.9872672536
H	1.0	-0.7359806804	4.8790040547	0.7375628426
H	1.0	2.4457787748	-2.3123572156	0.0180220823
C	6.0	-0.2716790931	-2.3165839348	-0.0149151344
H	1.0	0.1753229341	-3.3021570699	0.0187748384
C	6.0	-1.6462755597	-2.1563876179	-0.0657497931
N	7.0	-2.5578337206	-3.1626849777	-0.1251657043
H	1.0	-2.0601285556	-1.1555882268	-0.0991010489

C	6.0	-2.1436121566	-4.5407673636	0.0270474404
C	6.0	-3.9503250029	-2.8560264391	0.1282135457
H	1.0	-4.1825894688	-1.8618631730	-0.2596431788
H	1.0	-4.1851481651	-2.8732839817	1.2041884808
H	1.0	-4.5895074808	-3.5808273453	-0.3806410220
H	1.0	-1.3335840707	-4.7739614021	-0.6681920458
H	1.0	-2.9859021335	-5.1957005853	-0.1982260158
H	1.0	-1.7967063719	-4.7512366765	1.0504826713

Cartesian coordinates (Å) of the S_1 relaxed geometry for compound **4a** in DMSO

C	6.0	2.0019152971	-1.1403326272	0.0728316384
C	6.0	0.6541725469	-1.0733456972	0.0314668449
C	6.0	2.1211947139	1.2648600540	-0.0707593358
O	8.0	-0.0157771157	0.1136163757	-0.0616966221
C	6.0	0.7112137596	1.2986938803	-0.0664866428
C	6.0	2.8516675975	0.0458773281	0.0064964252
H	1.0	2.6560240423	2.2093376505	-0.1040487863
O	8.0	4.1172872644	-0.0334629350	0.0213987212
C	6.0	-0.0705863570	2.4528841257	-0.0748410141
H	1.0	0.4643288249	3.3951498174	-0.0686215160
C	6.0	-1.4888210528	2.4456501433	-0.0869743108
N	7.0	-2.2443960150	3.5496517252	0.0632216576
C	6.0	-3.6867256291	3.4537832987	-0.0436364497
H	1.0	-2.0197981028	1.5036395117	-0.1321389741
H	1.0	-4.0103280607	2.4274080543	0.1371385457
H	1.0	-4.0280829098	3.7627425544	-1.0428501699
H	1.0	-4.1584462408	4.1036496029	0.6985471159
C	6.0	-1.6698715105	4.8799450443	0.1099346139
H	1.0	-2.4473854614	5.5965073179	0.3731545002
H	1.0	-1.2529830314	5.1671646381	-0.8666084543
H	1.0	-0.8768506558	4.9409336384	0.8611879208
C	6.0	-0.2891593184	-2.2210103875	0.0789615589
H	1.0	2.4740021704	-2.1142915426	0.1453142846
H	1.0	0.2500434510	-3.1667398560	0.1481548354
H	1.0	-0.9171166600	-2.2391831629	-0.8192292107
H	1.0	-0.9593241247	-2.1330036742	0.9417651695

Cartesian coordinates (Å) of the S_1 relaxed geometry for compound **5a** in DMSO

C	6.0	1.9860997167	-1.3172429468	0.0399264266
C	6.0	0.5992446833	-1.2263807349	-0.0008975574
C	6.0	2.1414828996	1.0591849829	0.0040996566
O	8.0	-0.0263699028	0.0051219603	-0.0882482572
C	6.0	0.7536466045	1.1477010743	-0.0242336034
C	6.0	2.8536827911	-0.1809703861	0.0121982756
H	1.0	2.7027417898	1.9883789260	0.0351205313
O	8.0	4.1313742115	-0.2648106773	0.0159522943
C	6.0	0.0227543076	2.3474182238	0.0014432443
H	1.0	0.6027181108	3.2622357523	0.0272988184
C	6.0	-1.3691040881	2.3893292793	-0.0142067704
N	7.0	-2.1161131869	3.5033330668	0.0453602853
C	6.0	-3.5607635109	3.4294984306	-0.0772347885
H	1.0	-1.9226171467	1.4591676379	-0.0461409698
H	1.0	-3.8993580382	2.4062373099	0.0932797376
H	1.0	-3.8911394201	3.7489462613	-1.0743849791
H	1.0	-4.0328846417	4.0754837979	0.6687812523
C	6.0	-1.5095384376	4.8210465805	0.0472622441
H	1.0	-2.2780338638	5.5696915132	0.2400573516
H	1.0	-1.0414983525	5.0461080872	-0.9204289886
H	1.0	-0.7506866706	4.8996896304	0.8320113786
H	1.0	2.4206278779	-2.3110111565	0.0944837579

C	6.0	-0.2789708428	-2.3233745048	0.0273613145
H	1.0	0.1796005489	-3.3028411512	0.0933295528
C	6.0	-1.6630348122	-2.1947088528	-0.0355437191
N	7.0	-2.5394304803	-3.2124123674	-0.0558081724
H	1.0	-2.1009663769	-1.2047926688	-0.0752576052
C	6.0	-2.0984105925	-4.5911780957	0.0355814615
C	6.0	-3.9648876327	-2.9585785728	0.0564934067
H	1.0	-4.1753013747	-1.9058513126	-0.1391796758
H	1.0	-4.3286590410	-3.2066051142	1.0622131515
H	1.0	-4.5152151371	-3.5621527458	-0.6710643177
H	1.0	-1.3477644295	-4.8092246728	-0.7303724992
H	1.0	-2.9505675012	-5.2519710727	-0.1251301119
H	1.0	-1.6708741651	-4.8112761528	1.0231843098

Cartesian coordinates (Å) of the S_1 relaxed geometry for compound **4a** in ethanol

C	6.0	1.9970528836	-1.1429634672	0.0335107084
C	6.0	0.6475438697	-1.0682570814	0.0373600221
C	6.0	2.1283354537	1.2626715075	-0.0033163859
O	8.0	-0.0161337986	0.1276529022	0.0086789453
C	6.0	0.7221566184	1.3082961502	0.0279281044
C	6.0	2.8410887644	0.0379488930	-0.0204049211
H	1.0	2.6717121192	2.2022484635	-0.0167013270
O	8.0	4.1194425945	-0.0434773003	-0.0951883755
C	6.0	-0.0563977740	2.4667276113	0.0305108370
H	1.0	0.4773595555	3.4094436412	0.0486901714
C	6.0	-1.4712036658	2.4486437989	-0.0108989517
N	7.0	-2.2455523612	3.5454953406	0.0479872892
C	6.0	-3.6767577534	3.4207835731	-0.1574113001
H	1.0	-1.9902220845	1.4992850230	-0.0546014818
H	1.0	-4.0002044004	2.4000209565	0.0536563239
H	1.0	-3.9418138183	3.6654687686	-1.1971980861
H	1.0	-4.2137137265	4.1040447734	0.5053748507
C	6.0	-1.6907312483	4.8860260323	0.0724741449
H	1.0	-2.4838507626	5.5974563351	0.3014321758
H	1.0	-1.2578936126	5.1541360701	-0.9023070548
H	1.0	-0.9161766241	4.9758693435	0.8392126852
C	6.0	-0.3034999740	-2.2084223496	0.0634372328
H	1.0	2.4621192164	-2.1223315163	0.0481060777
H	1.0	0.2281105309	-3.1608113028	0.0788591975
H	1.0	-0.9546925183	-2.1810629455	-0.8180481034
H	1.0	-0.9499342676	-2.1495169160	0.9468438992

Cartesian coordinates (Å) of the S_1 relaxed geometry for compound **5a** in ethanol

C	6.0	1.9777500591	-1.3164903752	0.0334019871
C	6.0	0.5896566101	-1.2235967048	0.0306925050
C	6.0	2.1390472775	1.0588825871	0.0293749135
O	8.0	-0.0362461028	0.0114228383	-0.0007961109
C	6.0	0.7503728720	1.1528382227	0.0266784065
C	6.0	2.8349679812	-0.1823450875	0.0017767739
H	1.0	2.7044001208	1.9854043934	0.0417006989
O	8.0	4.1276910648	-0.2721293435	-0.0650248116
C	6.0	0.0264664413	2.3561639115	0.0308545348
H	1.0	0.6109135060	3.2683865763	0.0381911307
C	6.0	-1.3648687286	2.4027267420	0.0067219774
N	7.0	-2.1084479459	3.5185875914	0.0284069506
C	6.0	-3.5534409038	3.4466985031	-0.1042062192
H	1.0	-1.9213320104	1.4741467035	-0.0138843071
H	1.0	-3.8955410436	2.4244966601	0.0649043740
H	1.0	-3.8753365985	3.7654244469	-1.1040311315
H	1.0	-4.0305192659	4.0942269759	0.6376136978

C	6.0	-1.4985670919	4.8359026410	0.0050023677
H	1.0	-2.2672806517	5.5903386092	0.1736315462
H	1.0	-1.0219088733	5.0380267610	-0.9632377415
H	1.0	-0.7480170844	4.9317621053	0.7956889515
H	1.0	2.4082735104	-2.3126510977	0.0390276103
C	6.0	-0.2827424140	-2.3238465104	0.0366307547
H	1.0	0.1823410825	-3.3016617990	0.0728433999
C	6.0	-1.6677194171	-2.2039115601	-0.0201392638
N	7.0	-2.5338964278	-3.2282892740	-0.0663391499
H	1.0	-2.1142340351	-1.2170464979	-0.0401286824
C	6.0	-2.0810971257	-4.6056017757	0.0111645343
C	6.0	-3.9635481996	-2.9898987017	0.0388602417
H	1.0	-4.1840607994	-1.9375565365	-0.1476201434
H	1.0	-4.3324897490	-3.2525239833	1.0387273312
H	1.0	-4.5021098824	-3.5903367734	-0.6999395382
H	1.0	-1.3316211125	-4.8121400697	-0.7590406275
H	1.0	-2.9286833432	-5.2713632554	-0.1531192926
H	1.0	-1.6503377617	-4.8307104124	0.9959031227

Cartesian coordinates (Å) of the S_1 relaxed geometry for compound **4a** in methanol

C	6.0	1.9967642032	-1.1371144005	0.0804123092
C	6.0	0.6475802114	-1.0681319875	0.0259255524
C	6.0	2.1252408842	1.2623757072	-0.0828865835
O	8.0	-0.0171652517	0.1213429590	-0.0881420867
C	6.0	0.7188634790	1.3040163303	-0.1015980752
C	6.0	2.8396540242	0.0428792571	0.0105176420
H	1.0	2.6663279569	2.2030031272	-0.1119000055
O	8.0	4.1207865256	-0.0340856812	0.0526732157
C	6.0	-0.0625868717	2.4607445926	-0.0931608907
H	1.0	0.4702784046	3.4042069332	-0.0921621363
C	6.0	-1.4782109608	2.4433063798	-0.0736383498
N	7.0	-2.2401057290	3.5410296435	0.0683478377
C	6.0	-3.6844917509	3.4355940650	-0.0222383824
H	1.0	-2.0021486553	1.4964278291	-0.1105318097
H	1.0	-3.9990577561	2.4056370725	0.1531285889
H	1.0	-4.0386461061	3.7512488512	-1.0141304906
H	1.0	-4.1515590546	4.0746235543	0.7323277056
C	6.0	-1.6736168807	4.8766708456	0.1102360555
H	1.0	-2.4572632989	5.5899802191	0.3638841462
H	1.0	-1.2527058884	5.1608711337	-0.8645624810
H	1.0	-0.8868210566	4.9462617178	0.8675552720
C	6.0	-0.2996893571	-2.2100202127	0.0817506215
H	1.0	2.4598914822	-2.1131009762	0.1732275281
H	1.0	0.2345066726	-3.1576860997	0.1633131652
H	1.0	-0.9237213500	-2.2328361633	-0.8192001586
H	1.0	-0.9727089086	-2.1077435502	0.9410126409

Cartesian coordinates (Å) of the S_1 relaxed geometry for compound **5a** in methanol

C	6.0	1.9704765034	-1.3140971993	0.0337488623
C	6.0	0.5833853593	-1.2185035259	-0.0050952844
C	6.0	2.1407734713	1.0592861052	-0.0208024938
O	8.0	-0.0387127411	0.0165090718	-0.0660990367
C	6.0	0.7515778178	1.1566324169	-0.0464017583
C	6.0	2.8312143687	-0.1837075880	0.0154943252
H	1.0	2.7084374055	1.9845041381	-0.0080051055
O	8.0	4.1263953402	-0.2799589520	0.0574706567
C	6.0	0.0290198202	2.3605366956	-0.0358805115
H	1.0	0.6136575331	3.2727081164	-0.0331717371
C	6.0	-1.3628466950	2.4070910271	-0.0220698437
N	7.0	-2.1046302272	3.5216710667	0.0492177823

C	6.0	-3.5530487123	3.4537473149	-0.0464235788
H	1.0	-1.9194137129	1.4786517281	-0.0410820073
H	1.0	-3.8928172244	2.4306591111	0.1223343822
H	1.0	-3.8995750032	3.7820644184	-1.0347682921
H	1.0	-4.0081790323	4.0952262255	0.7137568416
C	6.0	-1.4943404905	4.8392484744	0.0417799930
H	1.0	-2.2593642430	5.5901302223	0.2396663247
H	1.0	-1.0358744063	5.0612859541	-0.9307090729
H	1.0	-0.7287468222	4.9180696268	0.8199591785
H	1.0	2.3932590243	-2.3123466234	0.0817787865
C	6.0	-0.2873529655	-2.3196900118	0.0148485201
H	1.0	0.1805840180	-3.2953225713	0.0687363916
C	6.0	-1.6727019235	-2.2063529209	-0.0375394395
N	7.0	-2.5328593290	-3.2361500439	-0.0577350406
H	1.0	-2.1246264422	-1.2221767155	-0.0683961522
C	6.0	-2.0706269374	-4.6096887875	0.0342757318
C	6.0	-3.9628636255	-3.0059267548	0.0612140176
H	1.0	-4.1926114591	-1.9569520068	-0.1327013563
H	1.0	-4.3184687266	-3.2611268494	1.0677732919
H	1.0	-4.5058762363	-3.6171579811	-0.6654170868
H	1.0	-1.3263591556	-4.8215605894	-0.7394023164
H	1.0	-2.9153782530	-5.2824975904	-0.1149178973
H	1.0	-1.6309343992	-4.8196294761	1.0183748821

4. Table S1. Ground state frontier orbitals for compounds 4a and 5a in vacuo

HOMO	LUMO
Ground state of **4a** 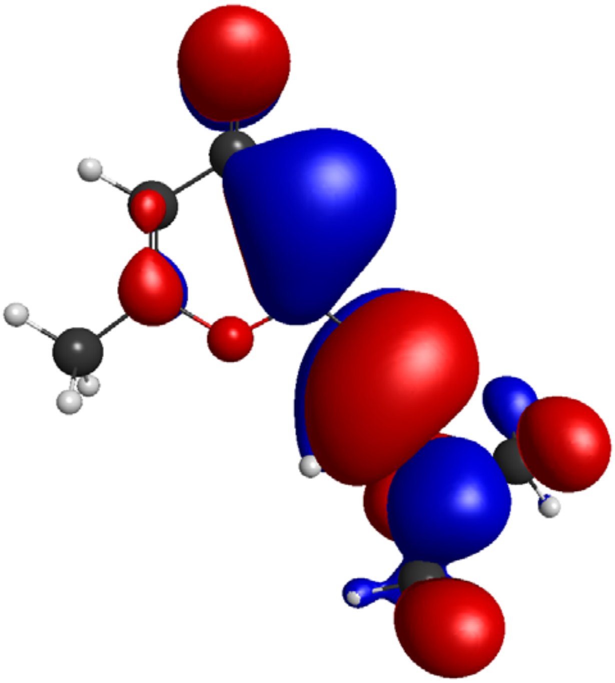 –5.50 eV	Ground state of **4a** 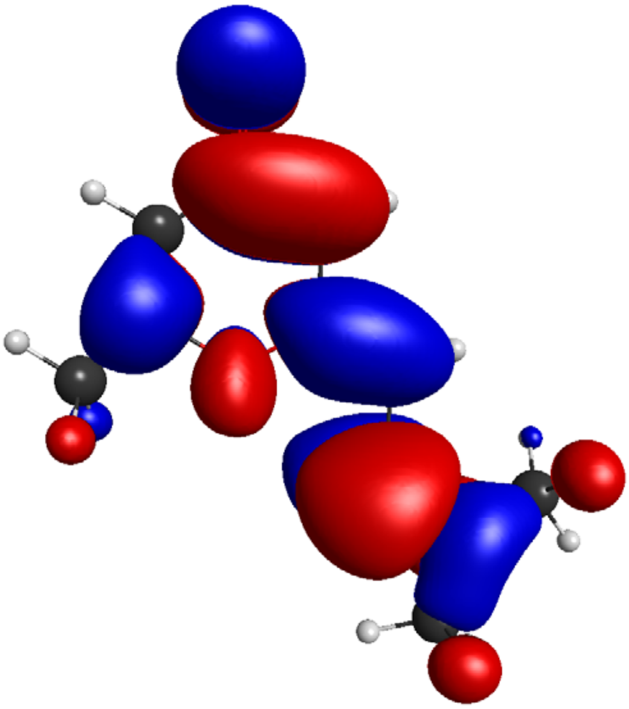 –1.22 eV
Ground state of **5a** 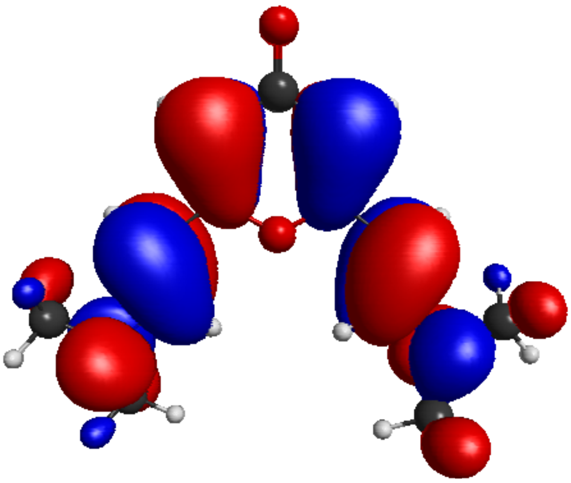 –5.20 eV	Ground state of **5a** 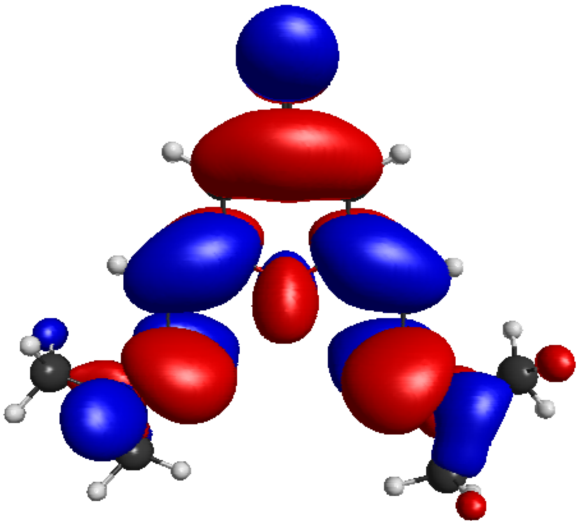 –1.20 eV

(isosurfaces correspond to 0.02psi/ 0.0004 rho)

5. Table S2. Ground state frontier orbitals for compounds 4a and 5a in DMSO

HOMO	LUMO
Ground state of **4a**<br>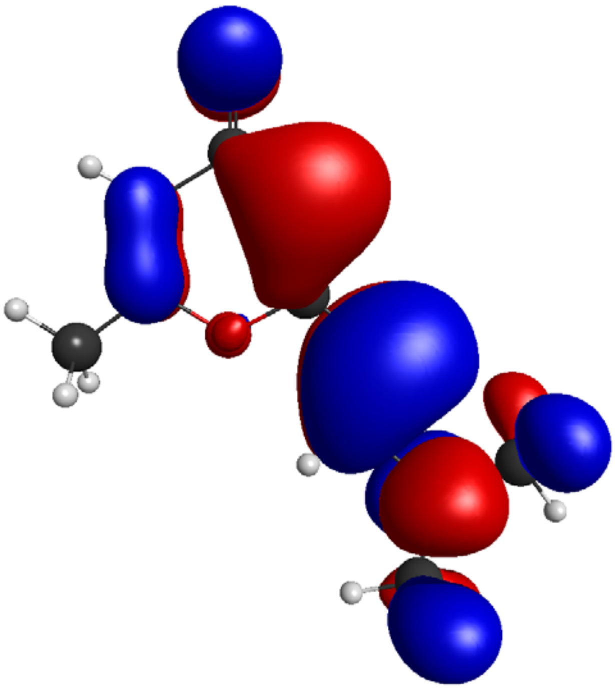<br>–5.28 eV	Ground state of **4a**<br>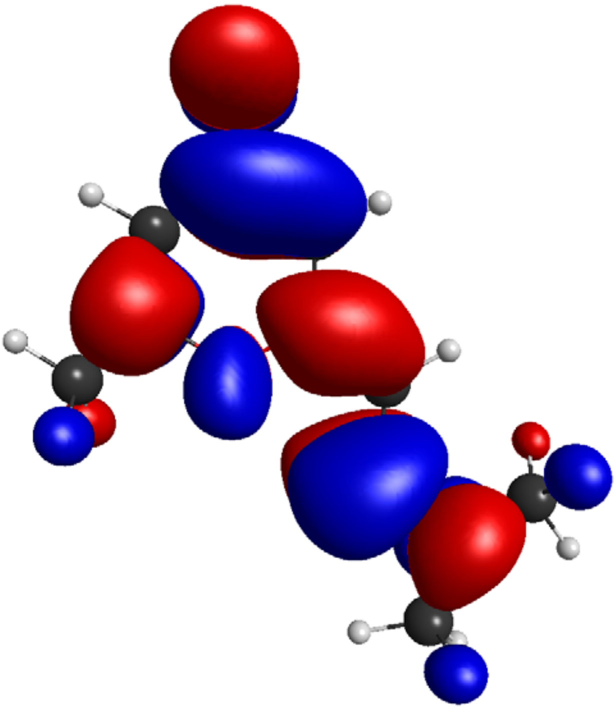<br>–1.20 eV
Ground state of **5a**<br>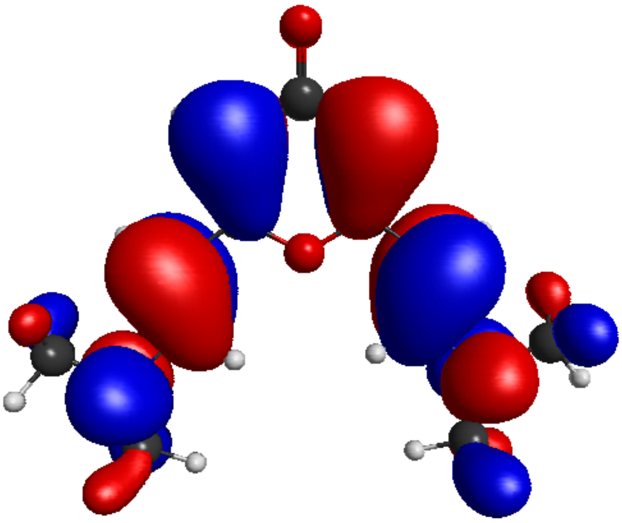<br>–5.01 eV	Ground state of **5a**<br>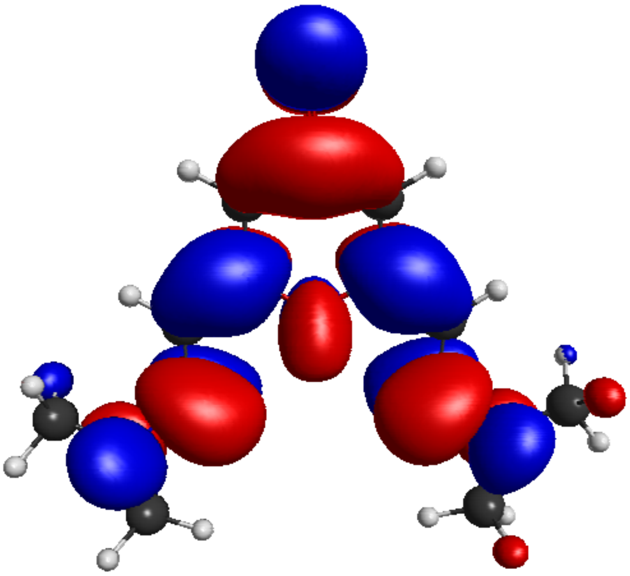<br>–1.25 eV

(isosurfaces correspond to 0.02psi/ 0.0004 rho)

6. Table S3. Ground state frontier orbitals for compounds 4a and 5a in ethanol

HOMO	LUMO
Ground state of **4a**<br>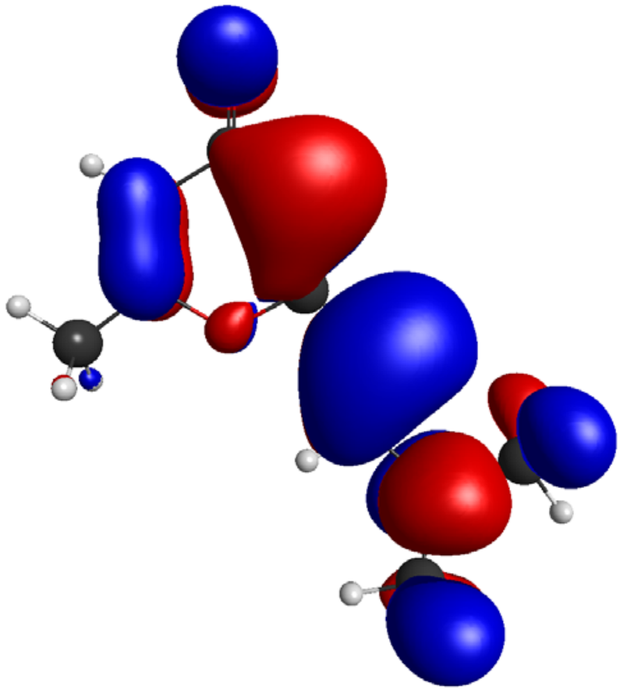<br>–5.36 eV	Ground state of **4a**<br>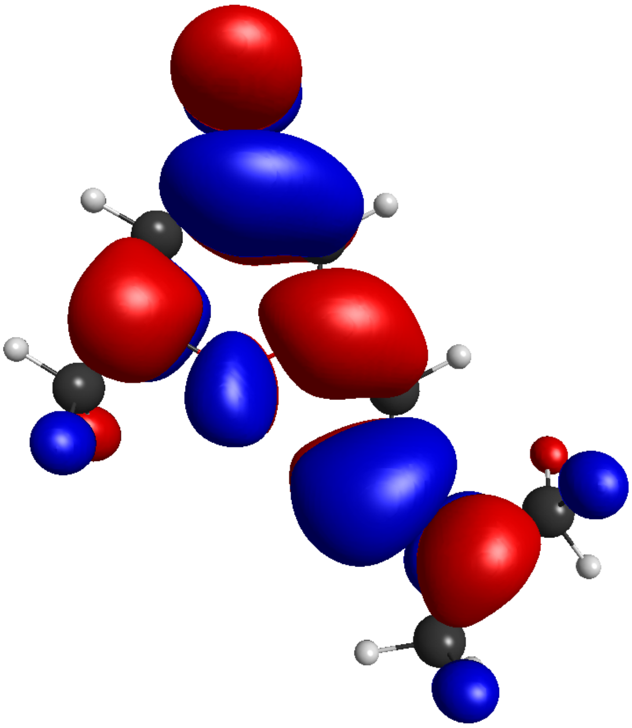<br>–1.36 eV
Ground state of **5a**<br>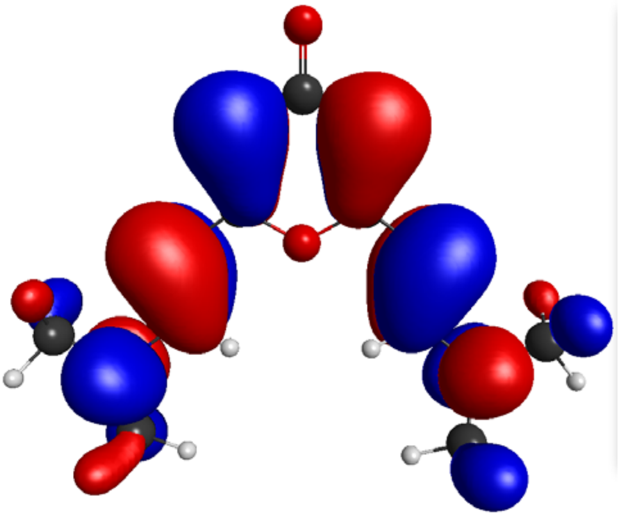<br>–5.09 eV	Ground state of **5a**<br>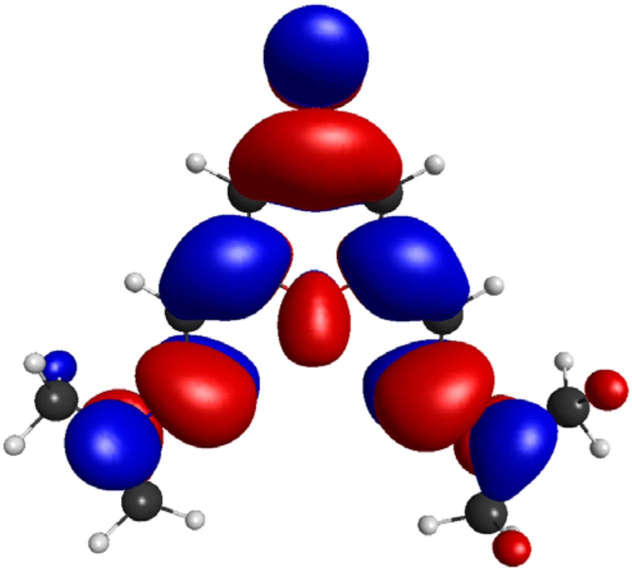<br>–1.41 eV

(isosurfaces correspond to 0.02psi/ 0.0004 rho)

7. Table S4. Ground state frontier orbitals for compounds 4a and 5a in methanol

HOMO	LUMO
Ground state of **4a**<br>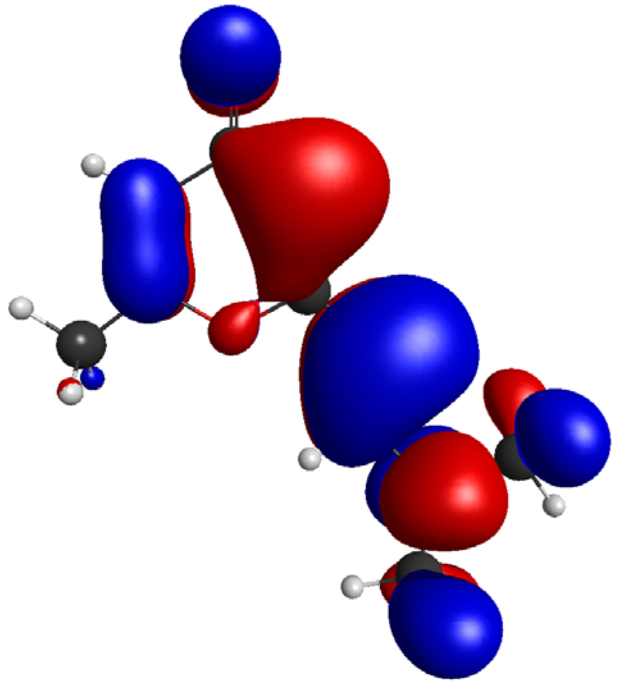<br>–5.36 eV	Ground state of **4a**<br>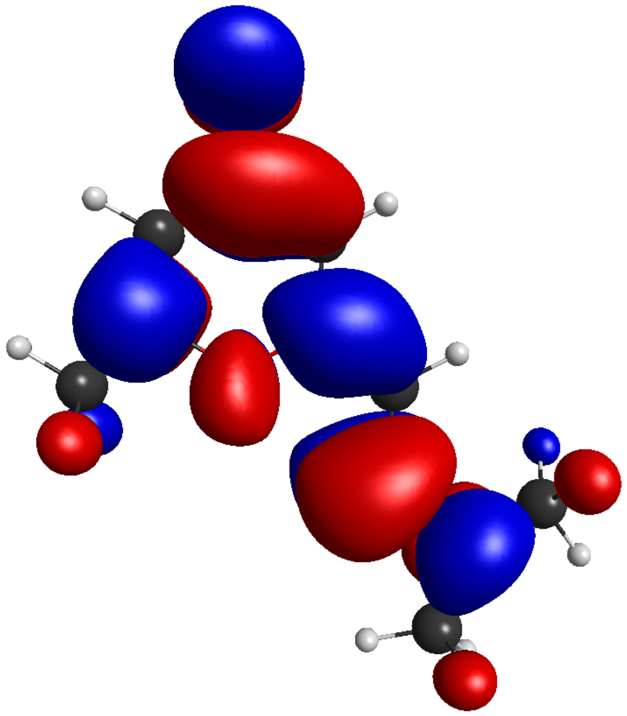<br>–1.36 eV
Ground state of **5a**<br>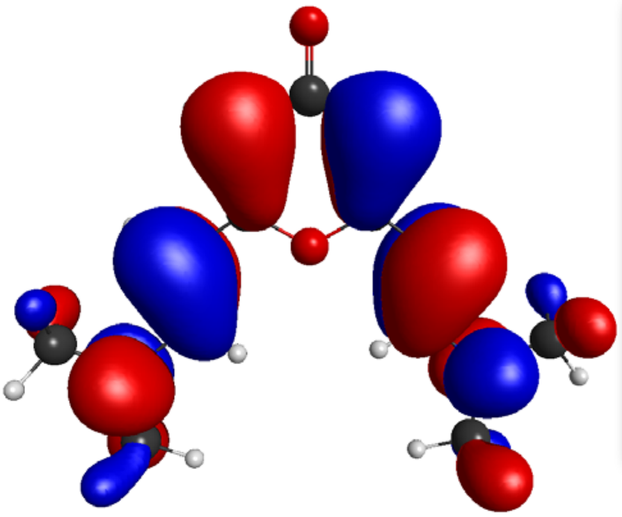<br>–5.06 eV	Ground state of **5a**<br>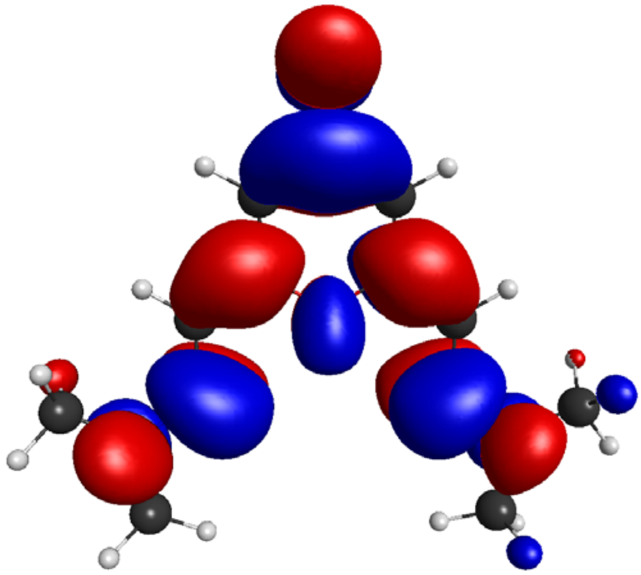<br>–1.41 eV

(isosurfaces correspond to 0.02psi/ 0.0004 rho)

8. Table S5. Frontier orbitals of the first singlet excited state for the relaxed geometry of compounds 4a and 5a in vacuo

HOMO	LUMO
S_1 excited state of **4a** 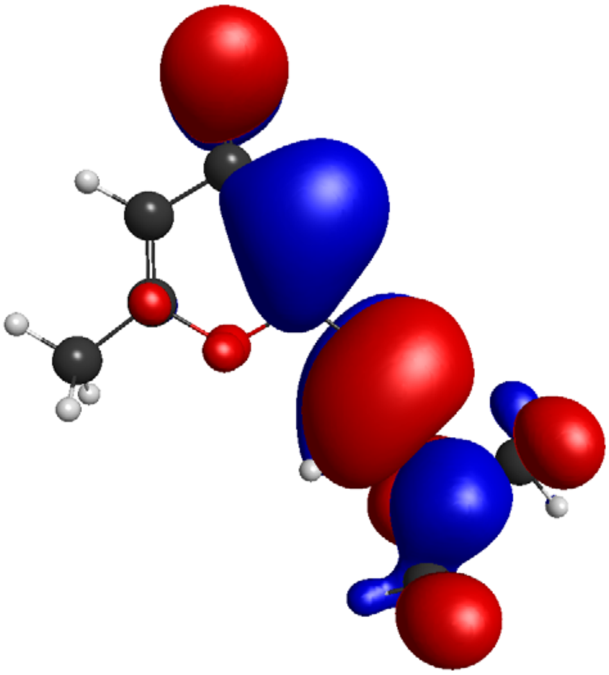 –7.05 eV	S_1 excited state of **4a** 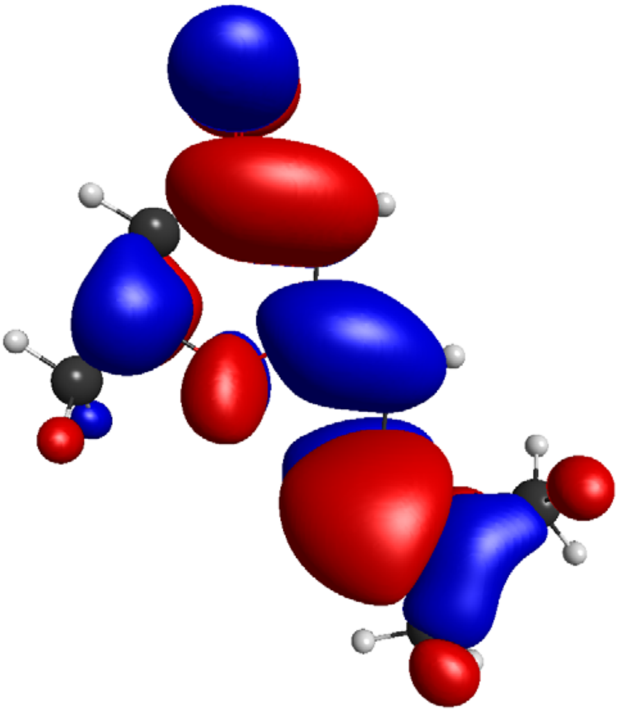 –0.46 eV
S_1 excited state of **5a** 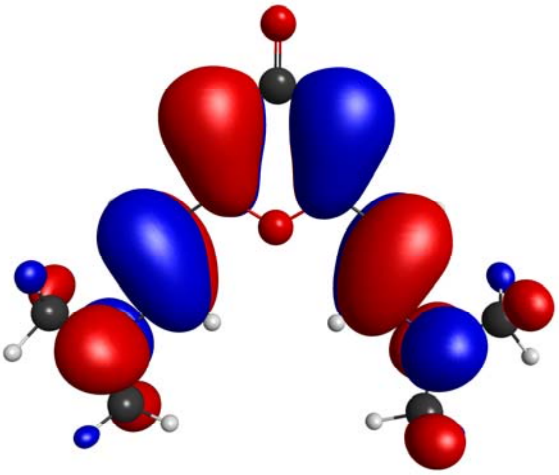 –6.37 eV	S_1 excited state of **5a** 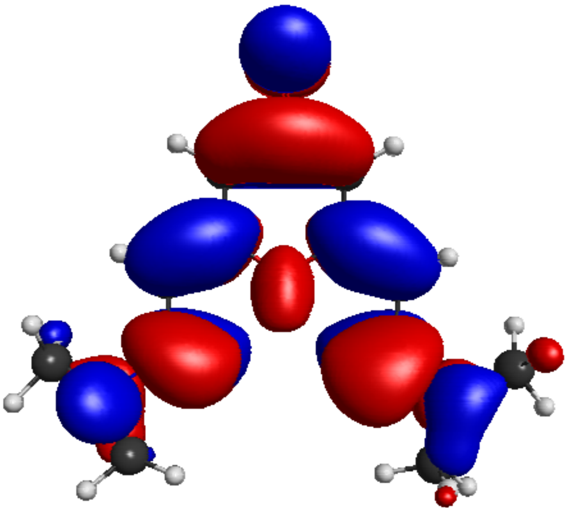 –0.33 eV

(isosurfaces correspond to 0.02psi/ 0.0004 rho)

9. Table S6. Frontier orbitals of the first singlet excited state for the relaxed geometry of compounds 4a and 5a in DMSO

HOMO	LUMO
S_1 excited state of **4a**<br>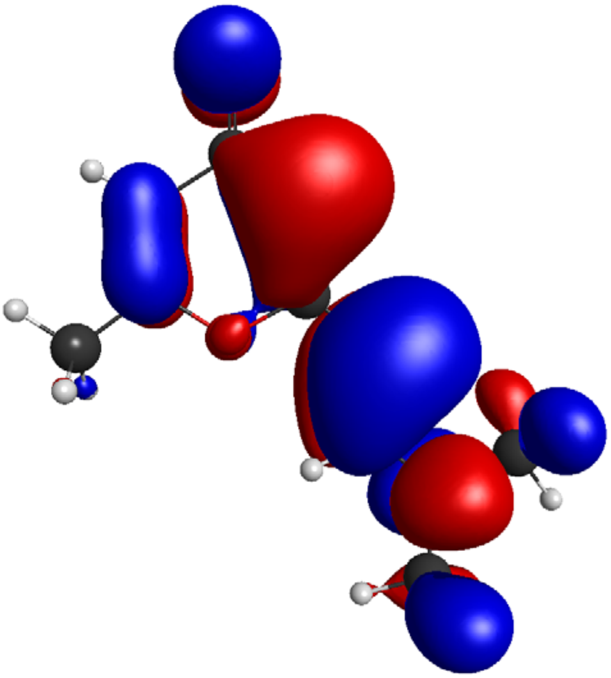<br>–6.59 eV	S_1 excited state of **4a**<br>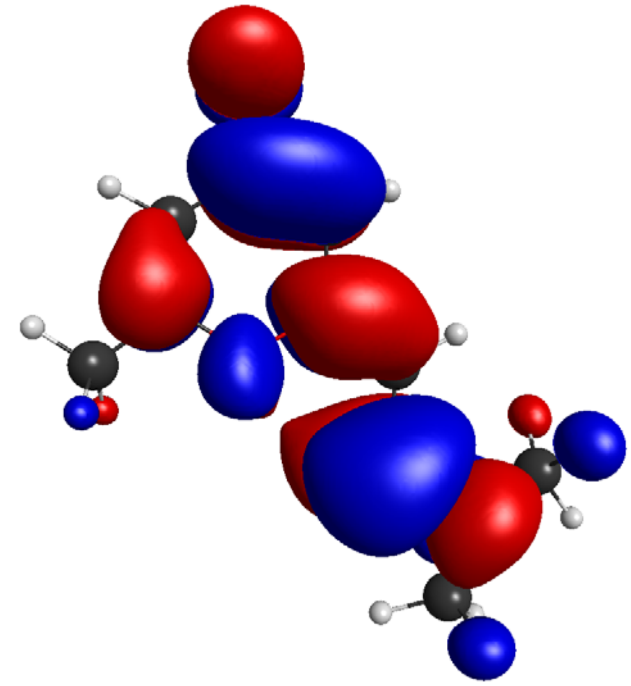<br>–0.38 eV
S_1 excited state of **5a**<br>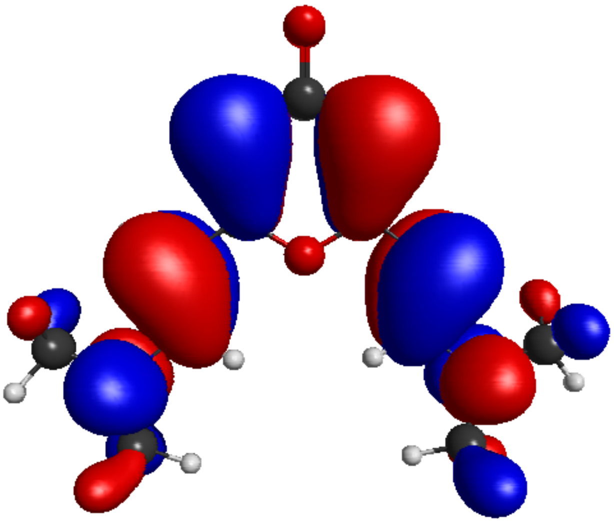<br>–6.23 eV	S_1 excited state of **5a**<br>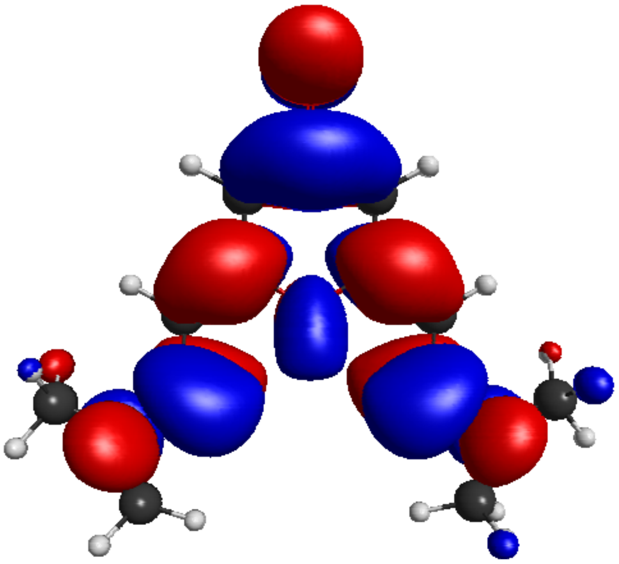<br>–0.38 eV

(isosurfaces correspond to 0.02psi/ 0.0004 rho)

10. Table S7. Frontier orbitals of the first singlet excited state for the relaxed geometry of compounds 4a and 5a in ethanol

HOMO	LUMO
S_1 excited state of **4a**<br>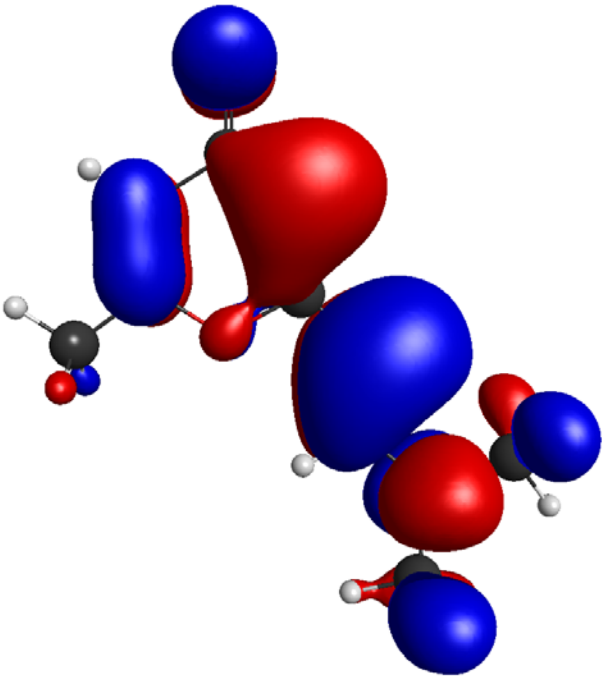<br>–6.69 eV	S_1 excited state of **4a**<br>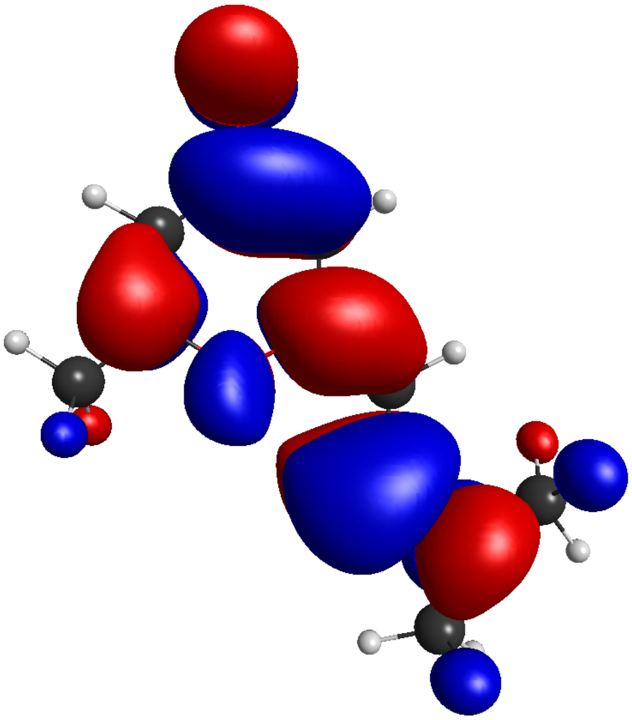<br>–0.52 eV
S_1 excited state of **5a**<br>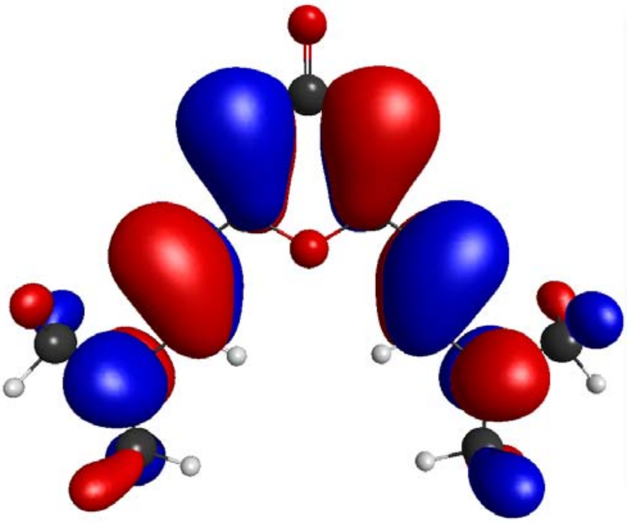<br>–6.25 eV	S_1 excited state of **5a**<br>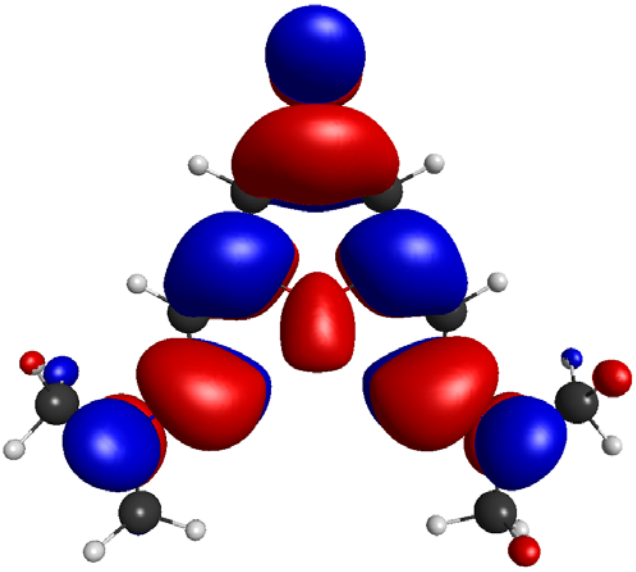<br>–0.52 eV

(isosurfaces correspond to 0.02psi/ 0.0004 rho)

11. Table S8. Frontier orbitals of the first singlet excited state for the relaxed geometry of compounds 4a and 5a in methanol

HOMO	LUMO
S_1 excited state of **4a**<br>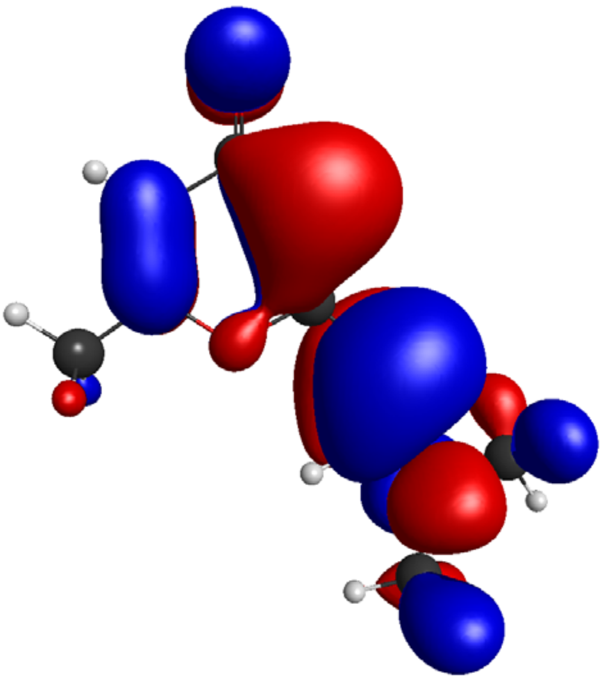<br>–6.69 eV	S_1 excited state of **4a**<br>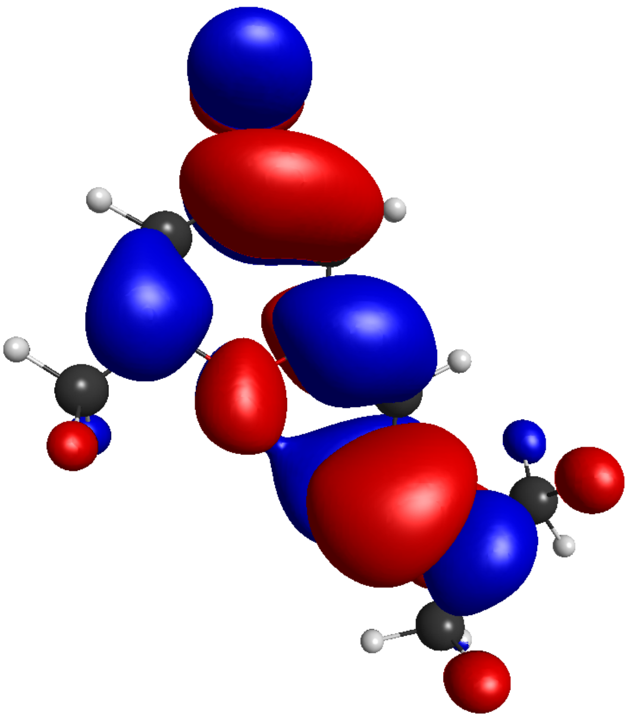<br>–0.52 eV
S_1 excited state of **5a**<br>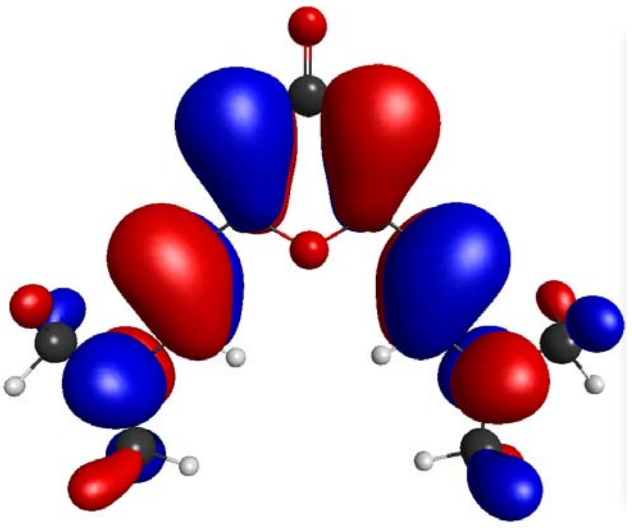<br>–6.29 eV	S_1 excited state of **5a**<br>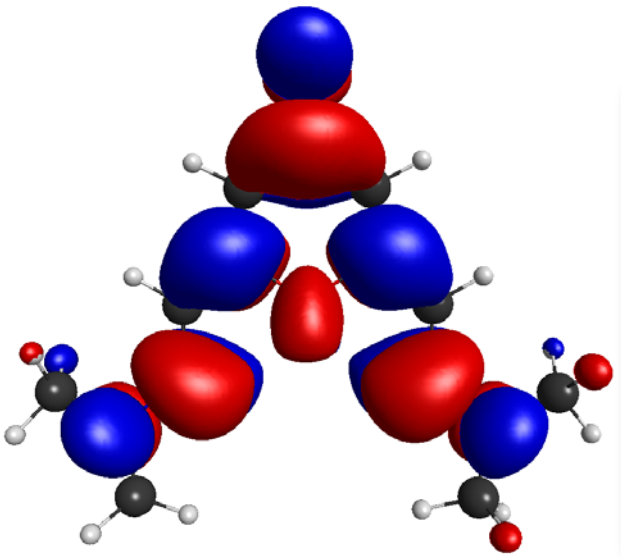<br>–0.54 eV

(isosurfaces correspond to 0.02psi/ 0.0004 rho)

12. Calculated normalized UV-vis spectra for compounds 4a and 5a in DMSO, methanol, and ethanol

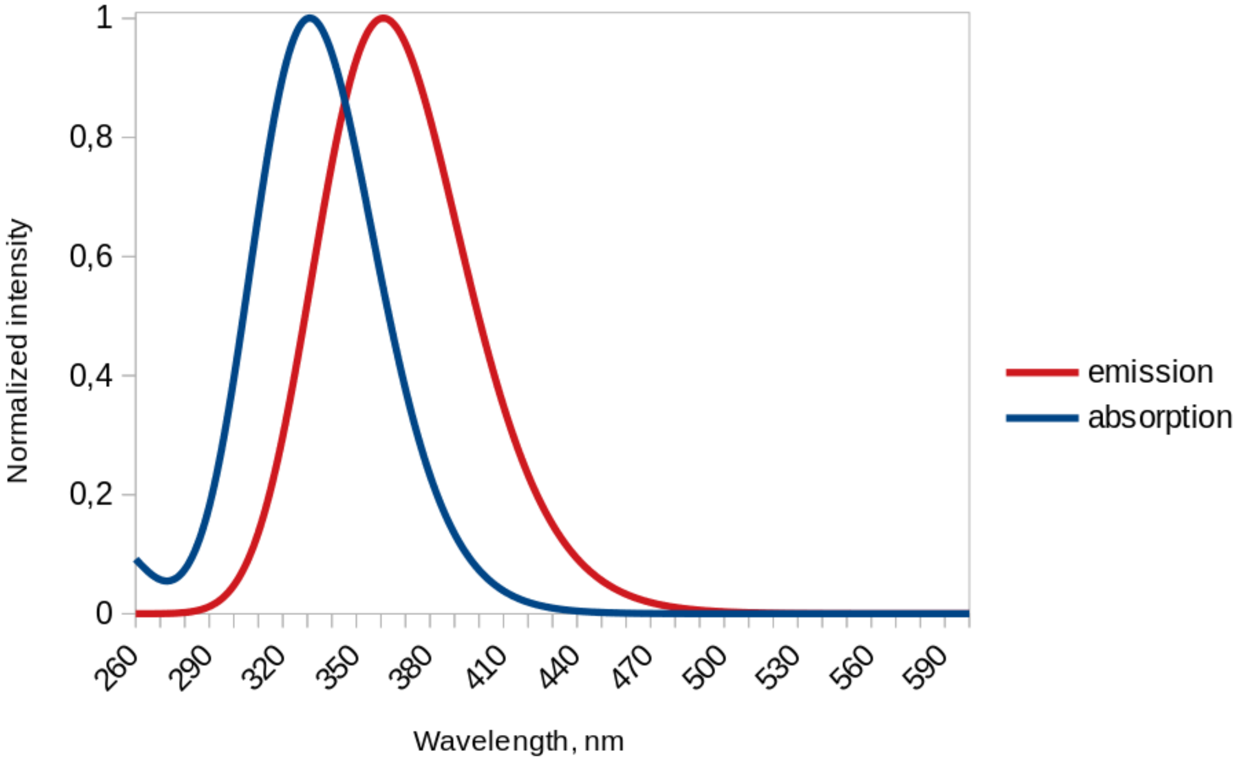

Figure S1. Normalized absorption and emission spectra of **4a** in DMSO at a CAM-B3LYP level.

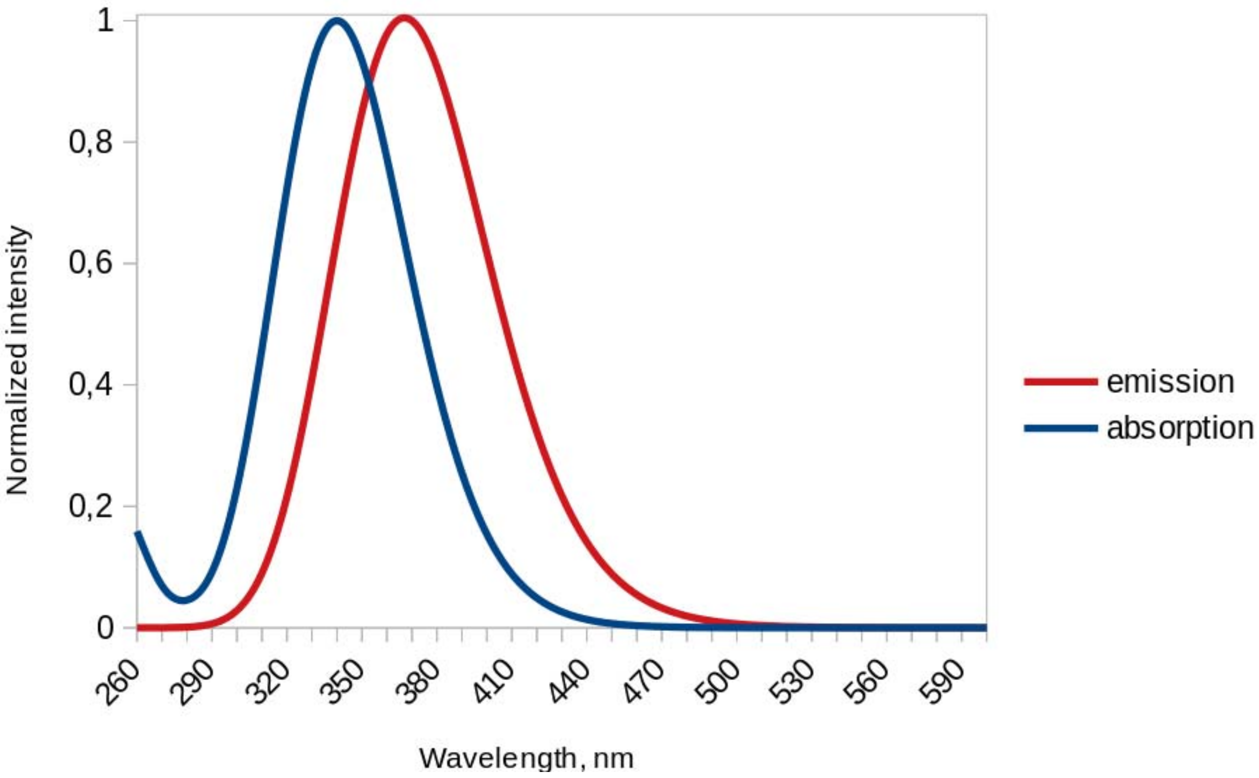

Figure S2. Normalized absorption and emission spectra of **4a** in ethanol at a CAM-B3LYP level.

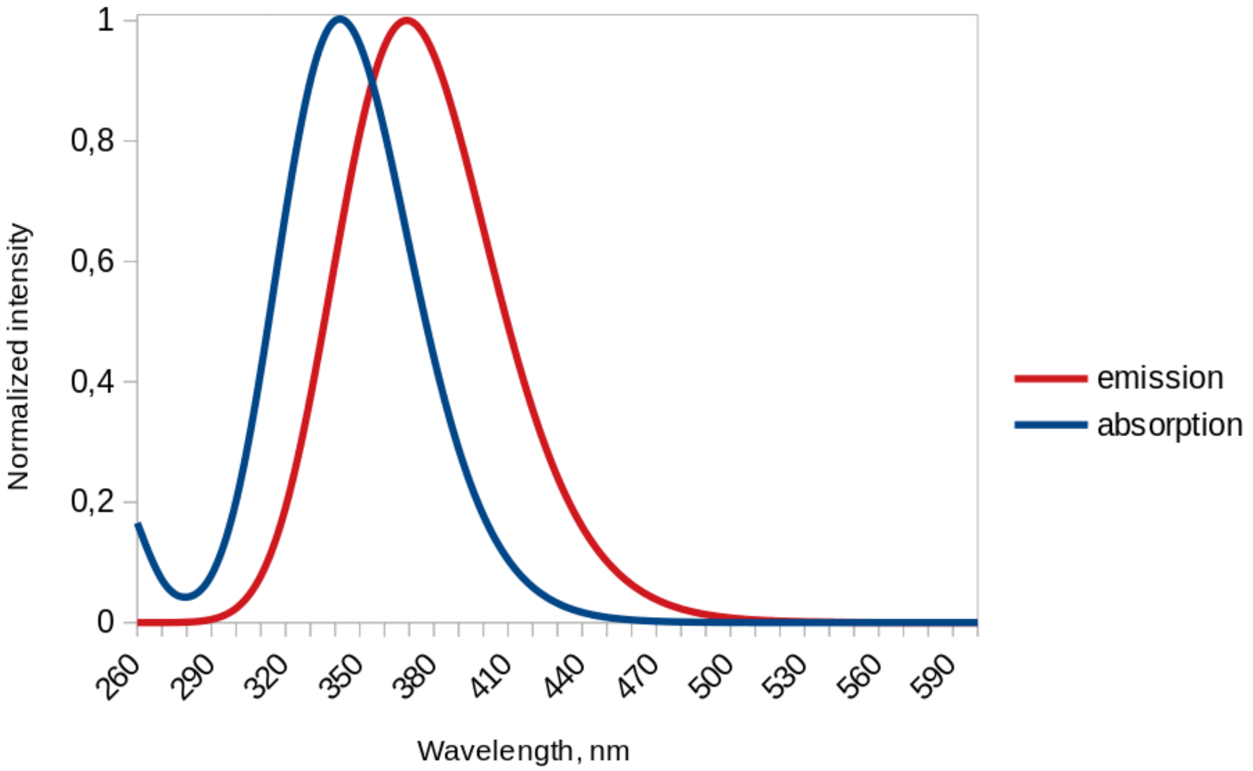

Figure S3. Normalized absorption and emission spectra of **4a** in methanol at a CAM-B3LYP level.

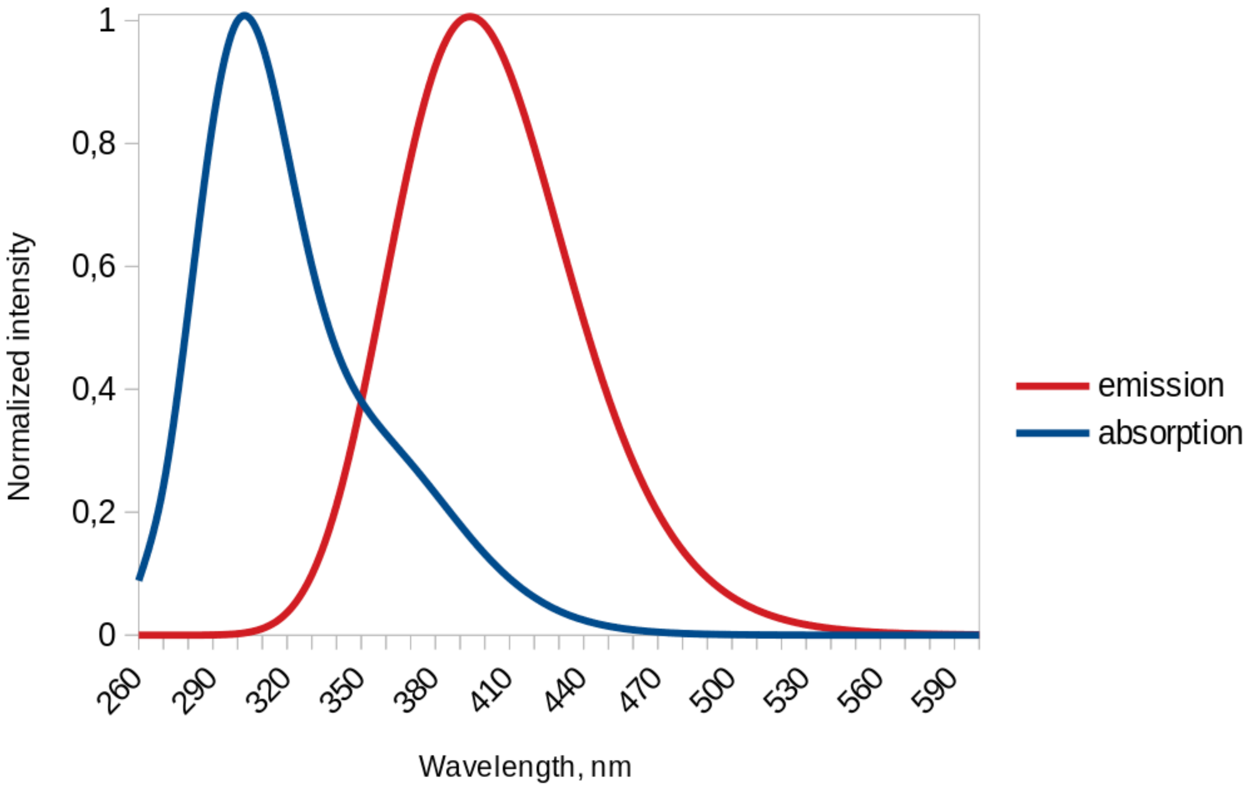

Figure S4. Normalized absorption and emission spectra of **5a** in DMSO at a CAM-B3LYP level.

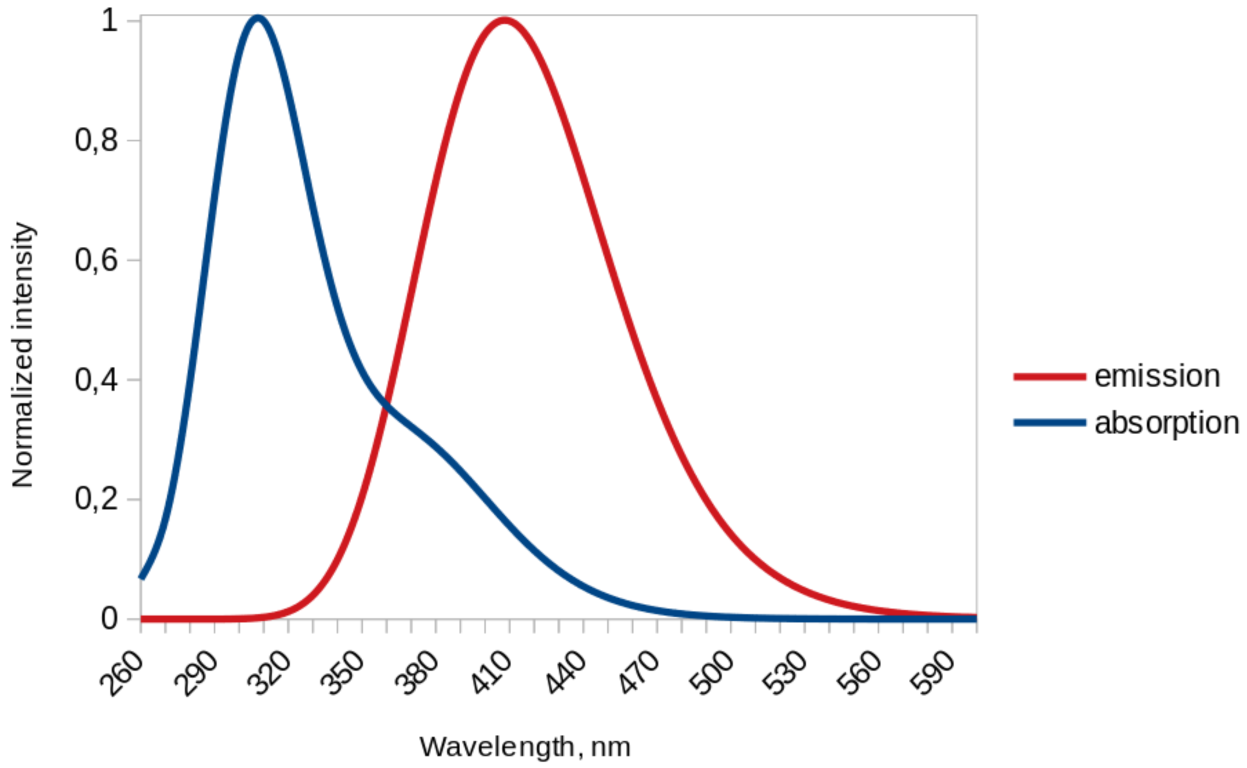

Figure S5. Normalized absorption and emission spectra of **5a** in ethanol at a CAM-B3LYP level.

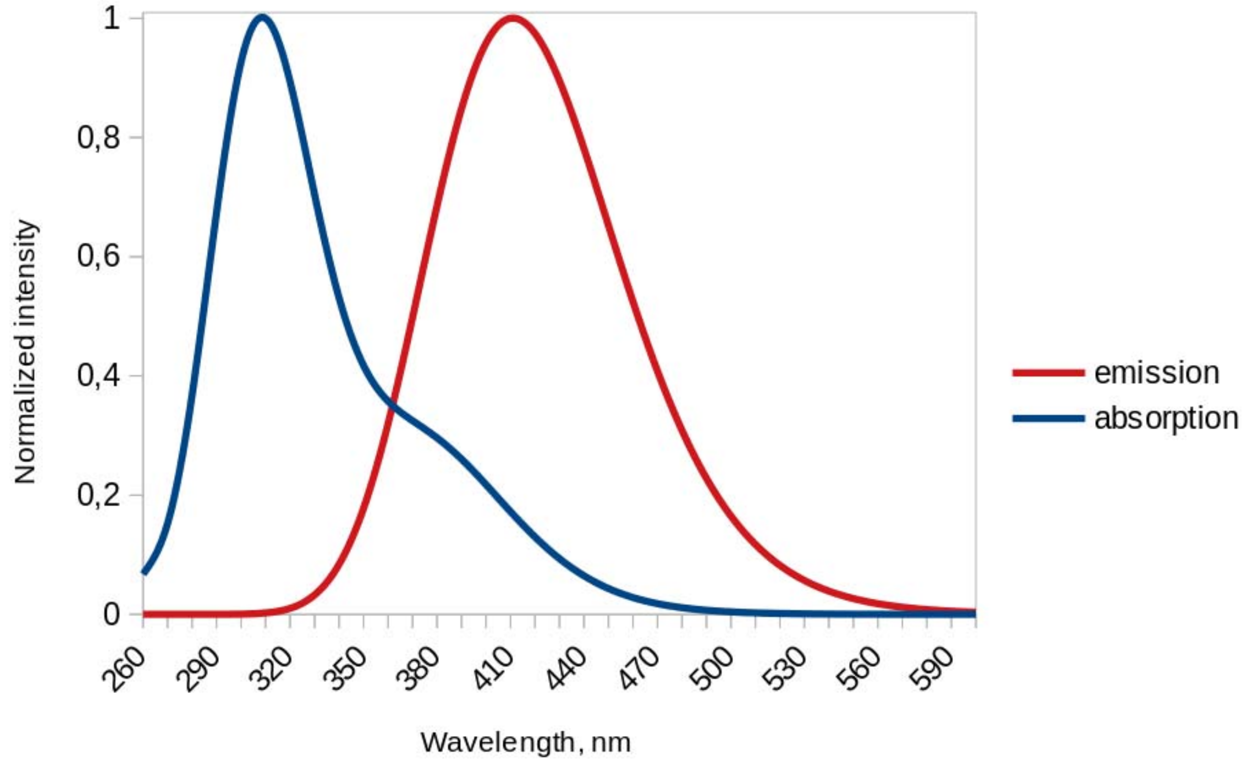

Figure S6. Normalized absorption and emission spectra of **5a** in methanol at a CAM-B3LYP level.

13. Electron density difference maps for compounds 4a and 5a

Blue and green regions correspond to the increase and decrease in electronic density respectively.

Electron density difference maps were calculated with the use of Multiwfn v3.8 [7].

7. Lu, T.; Chen, F. Multiwfn: A multifunctional wavefunction analyzer. *J. Comput. Chem.* **2012**, *33*, 580–592. doi: 10.1002/jcc.22885.

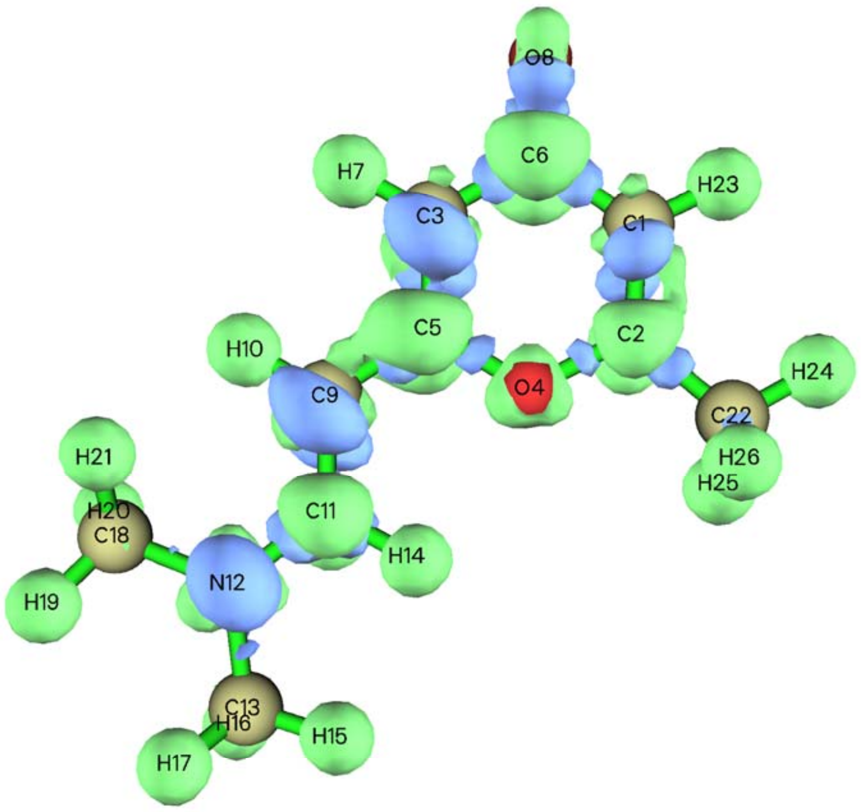

Figure S7. An electron density difference map for the $S_0 \rightarrow S_1$ transition of compound **4a** in vacuo.

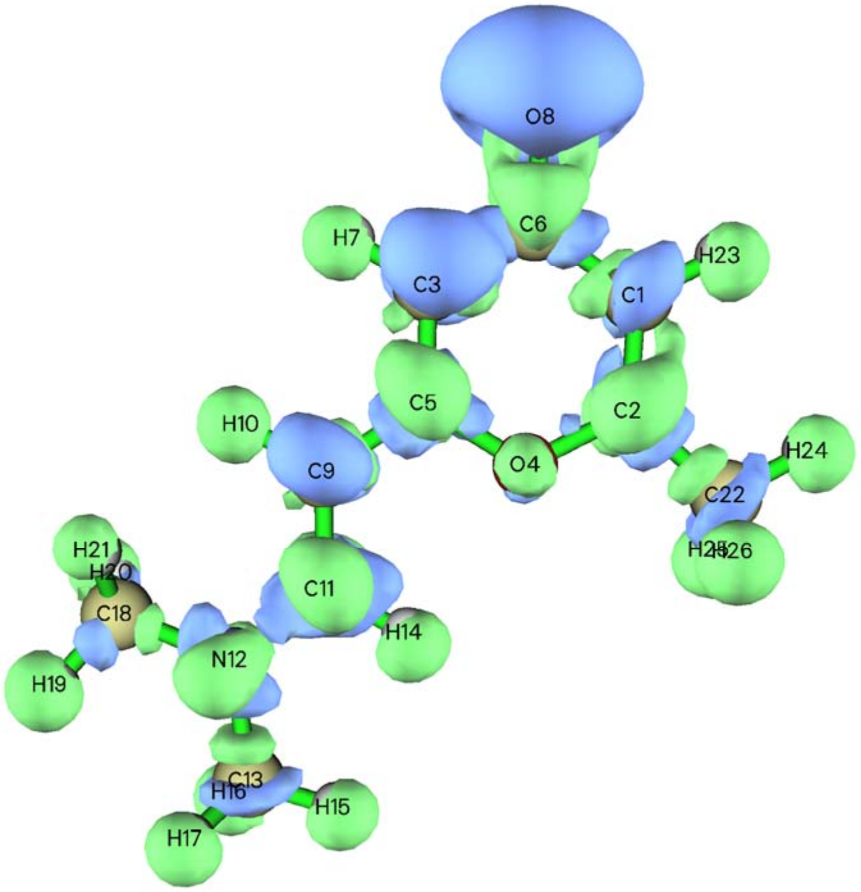

Figure S8. An electron density difference map for the $S_0 \rightarrow S_1$ transition of compound **4a** in methanol.

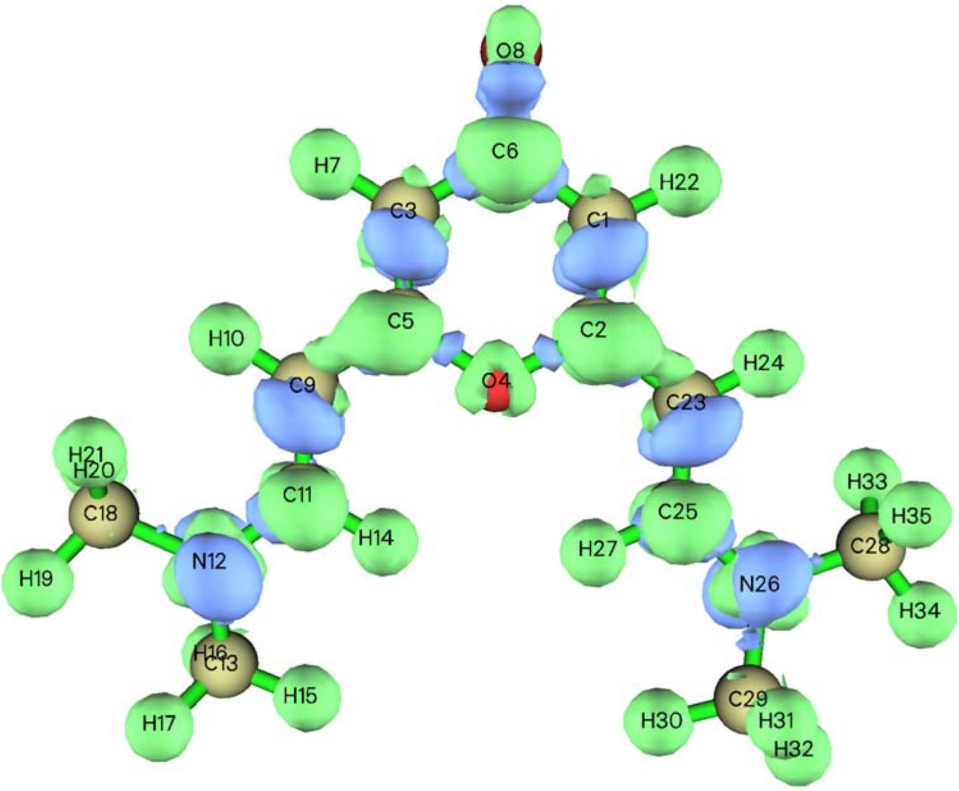

Figure S9. An electron density difference map for the $S_0 \rightarrow S_1$ transition of compound **5a** in vacuo.

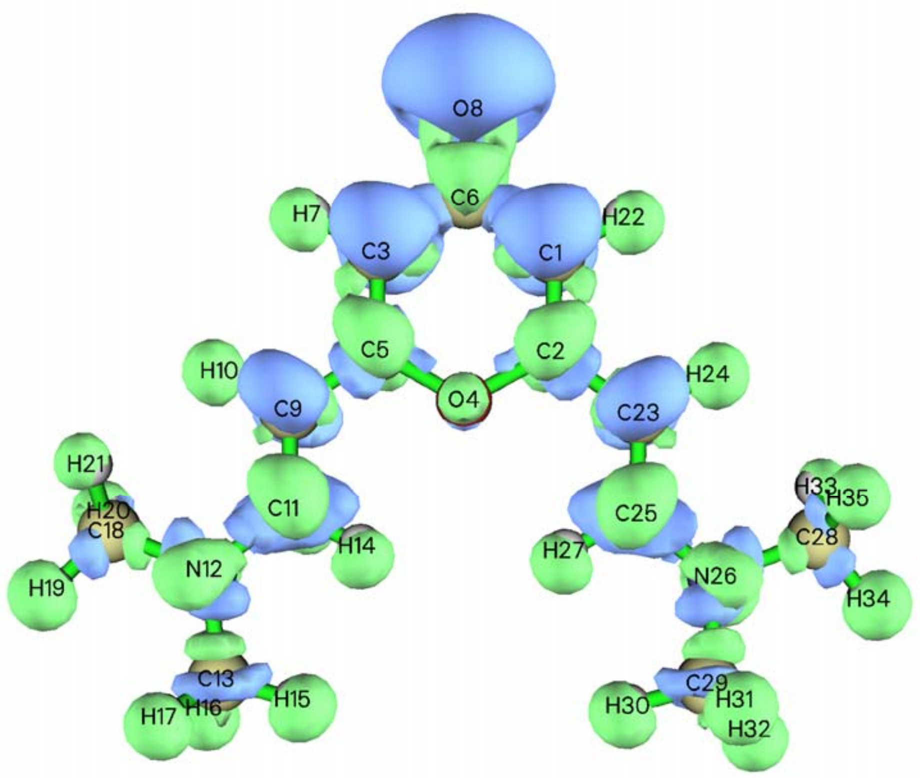

Figure S10. An electron density difference map for the $S_0 \rightarrow S_1$ transition of compound **5a** in methanol.

The method of preparation of 2-methyl-6-trifluoromethyl-4-pyrone (1c)

2-Methyl-6-(trifluoromethyl)-4*H*-pyran-4-one (1c).

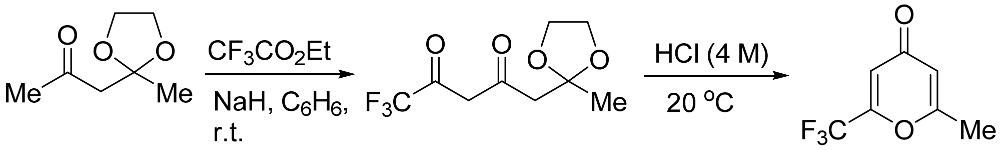

1c

NaH (60% dispersion in mineral oil) (1.67 g, 0.0416 mol) was added to the solution of 1-(2-methyl-1,3-dioxolan-2-yl)propan-2-one [8] (5.0 g, 0.0347 mol) and ethyl 2,2,2-trifluoroacetate (5.92 g, 0.0416 mol) in benzene (20 mL), and the reaction mixture was stirred at room temperature overnight. The obtained precipitate of the salt was filtered. The solid was stirred in HCl (40 mL, 4 M) at 0 °C for 1 h and then at room temperature overnight. The product was extracted with $CHCl_3$ (3×15 mL), dried on Na_2SO_4, evaporated. *n*-Hexane was added to the residue The *n*-hexane solution was cooled at –20°C overnight. The white crystals were obtained and filtered. Yield 1.85 g (30 %), mp 49–50 °C, white crystals.

^{1}H NMR (400 MHz, $CDCl_3$) δ 2.37 (3H, d, J = 0.8 Hz, Me), 6.26 (1H, d.q, J = 2.2 Hz, J = 0.8 Hz, H-3), 6.67 (1H, d, J = 2.2 Hz, H-5). ^{19}F NMR (376 MHz, $CDCl_3$) δ 90.1 (s, CF_3).

The NMR spectra are in accordance with the literature data [9].

8. Dorman, L. C. Synthesis of 2-methyl-4*H*-pyran-4-one. *J. Org. Chem.* **1967**, 32. 4105–4107. doi: 10.1021/jo01287a101.

9. Tyvorskii, V.I.; Bobrov, D.N.; Kulinkovich, O.G.; De Kimpe, N.; Tehrani, K.A. New synthetic approaches to 2-perfluoroalkyl-4*H*-pyran-4-ones. Tetrahedron **1998**, *54*, 2819–2826. doi: 10.1016/S0040-4020(98)83018-4.

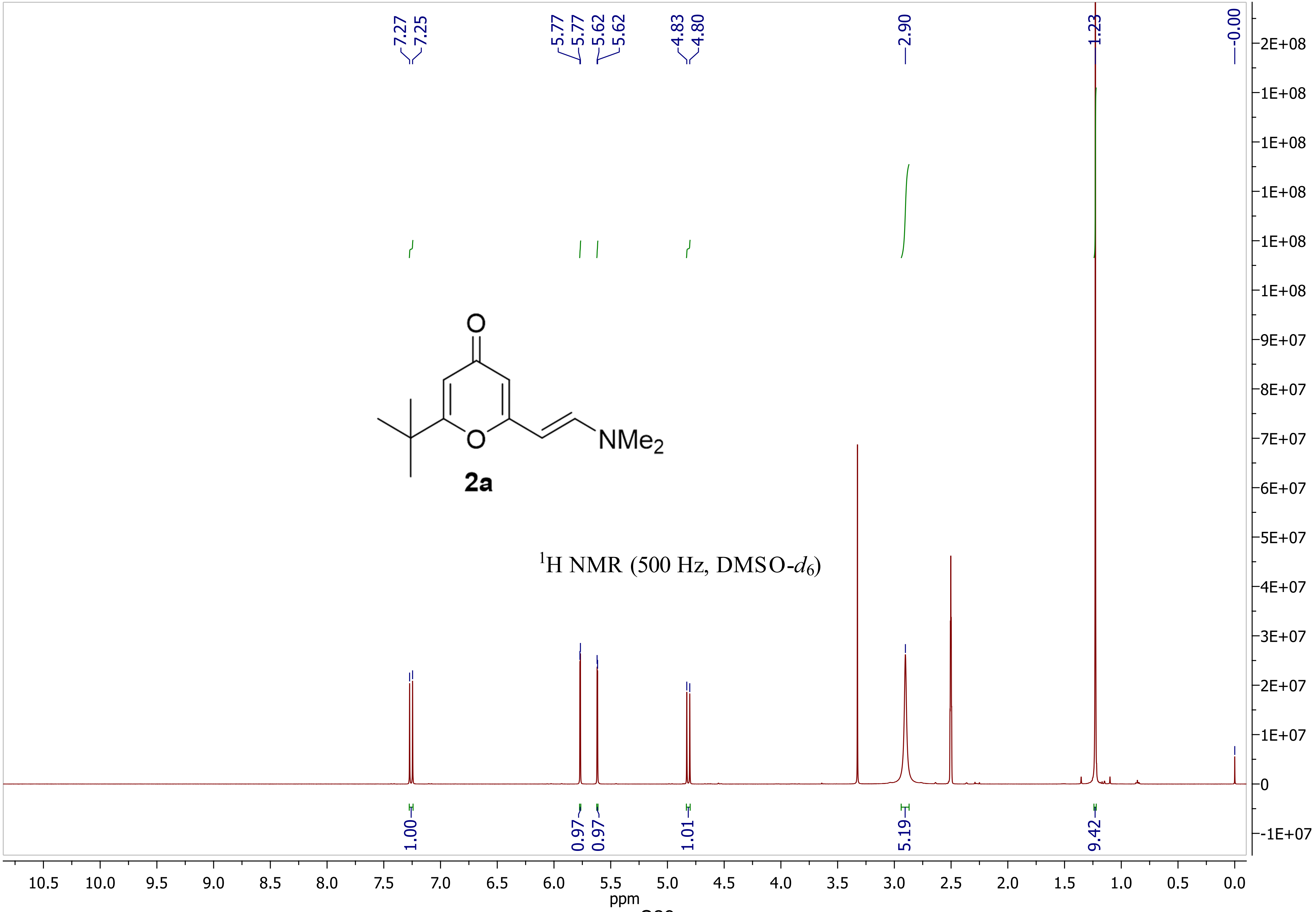
7.27
7.25
5.77
5.77
5.62
5.62
4.83
4.80
2.90
1.23
-0.00
O
O
NMe2
2a
1H NMR (500 Hz, DMSO-d6)
1.00
0.97
0.97
1.01
5.19
9.42
ppm

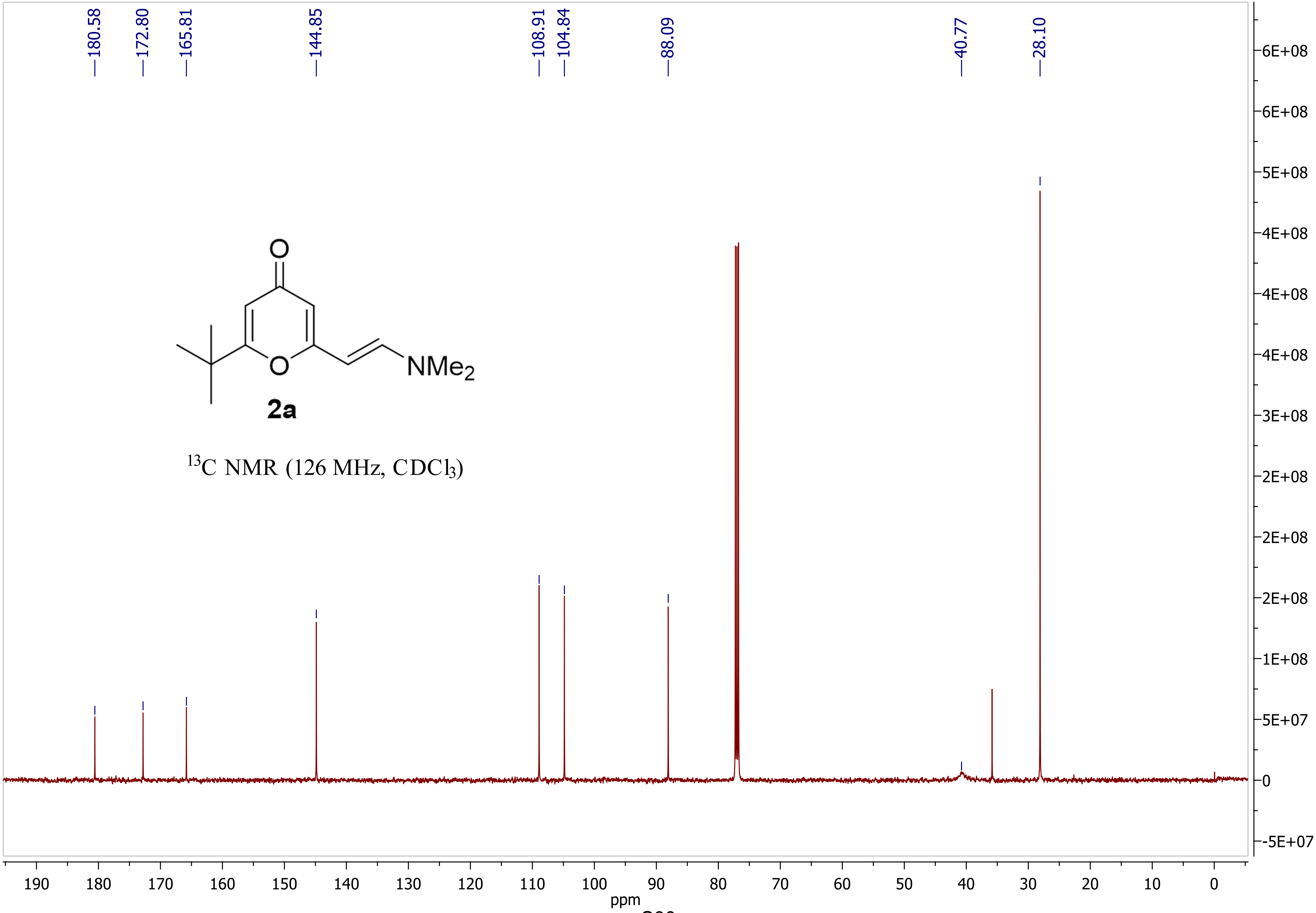

180.58
172.80
165.81
144.85
108.91
104.84
88.09
40.77
28.10
NMe2
2a
13C NMR (126 MHz, CDCl3)
6E+08
6E+08
5E+08
4E+08
4E+08
4E+08
3E+08
2E+08
2E+08
2E+08
1E+08
5E+07
0
-5E+07
190
180
170
160
150
140
130
120
110
100
90
80
70
60
50
40
30
20
10
0
ppm

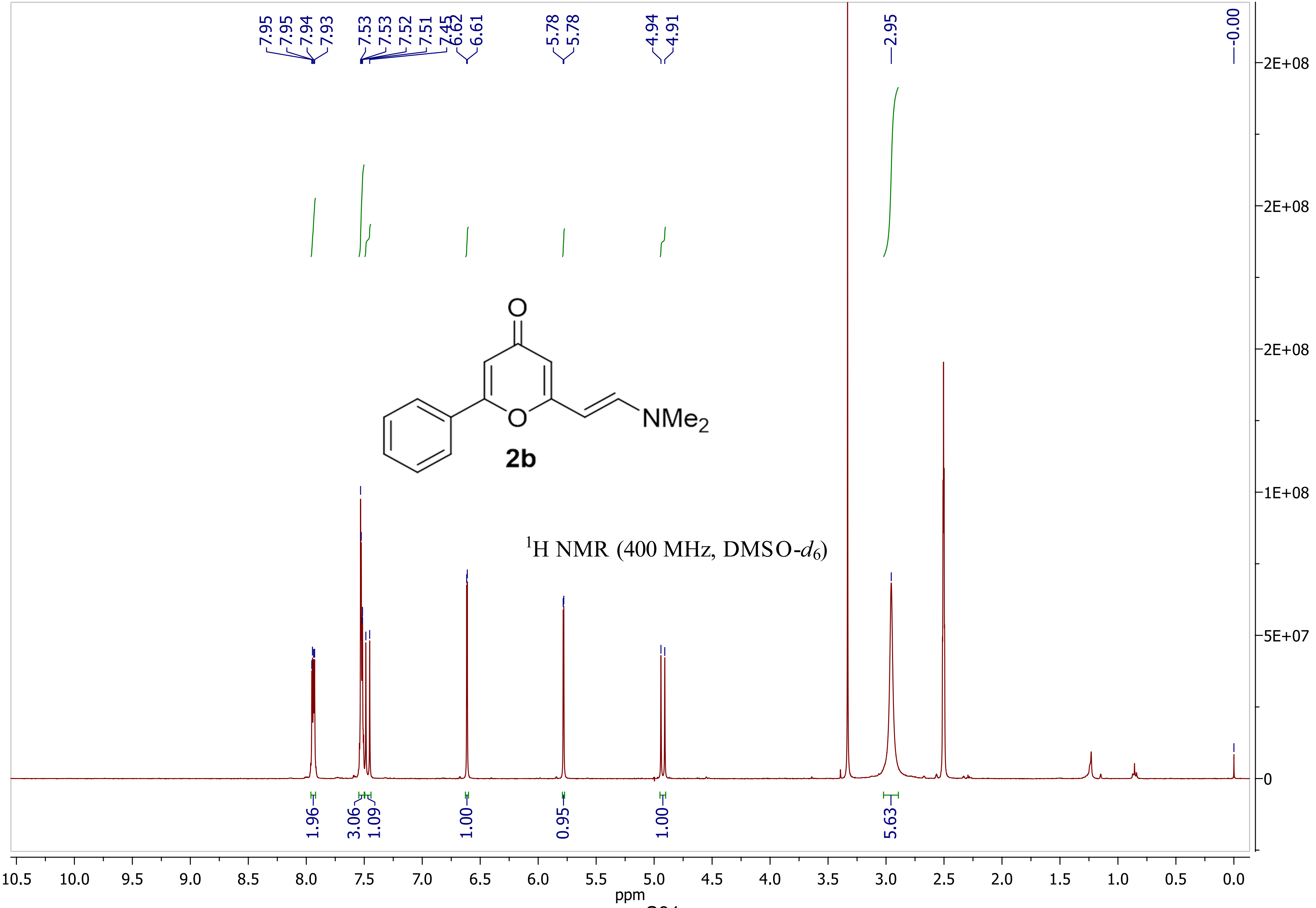

7.95
7.95
7.94
7.93
7.53
7.53
7.52
7.51
7.45
6.62
6.61
5.78
5.78
4.94
4.91
2.95
-0.00
2b
NMe2
1H NMR (400 MHz, DMSO-d6)
1.96
3.06
1.09
1.00
0.95
1.00
5.63
ppm

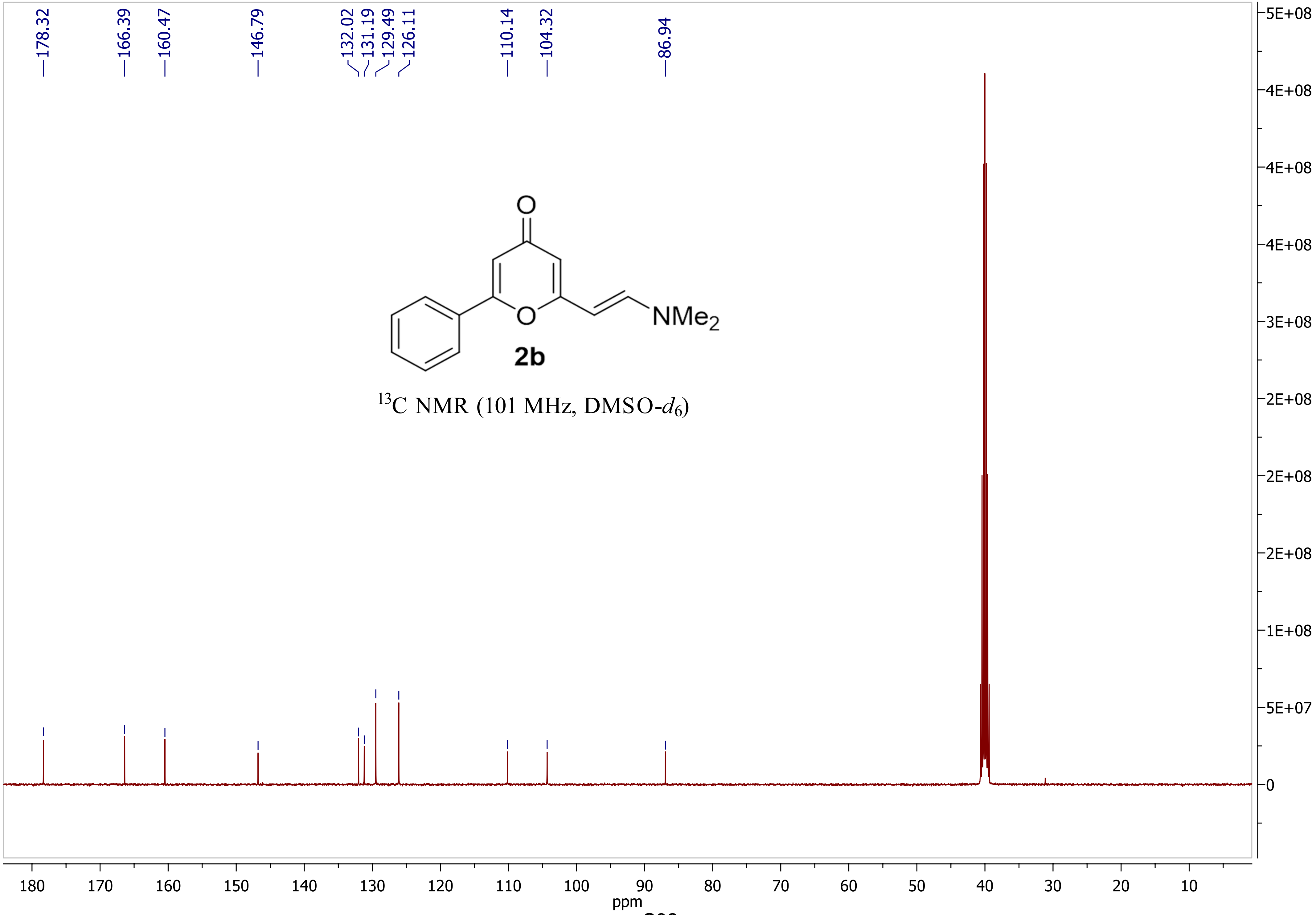
178.32
166.39
160.47
146.79
132.02
131.19
129.49
126.11
110.14
104.32
86.94
O
O
NMe2
2b
13C NMR (101 MHz, DMSO-d6)
5E+08
4E+08
4E+08
4E+08
3E+08
2E+08
2E+08
2E+08
1E+08
5E+07
0
180
170
160
150
140
130
120
110
100
90
80
70
60
50
40
30
20
10
ppm

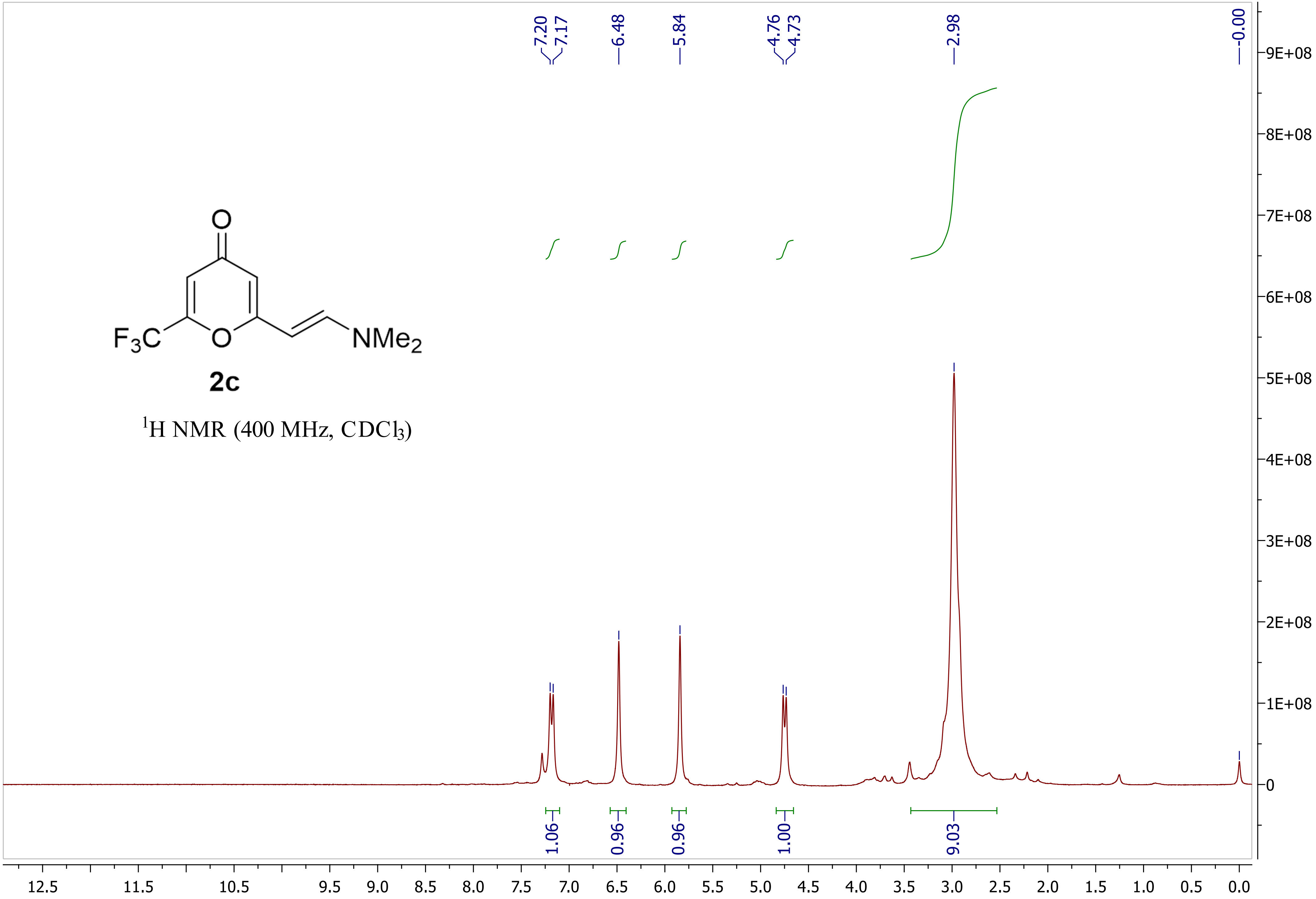

2c

^{1}H NMR (400 MHz, $CDCl_3$)

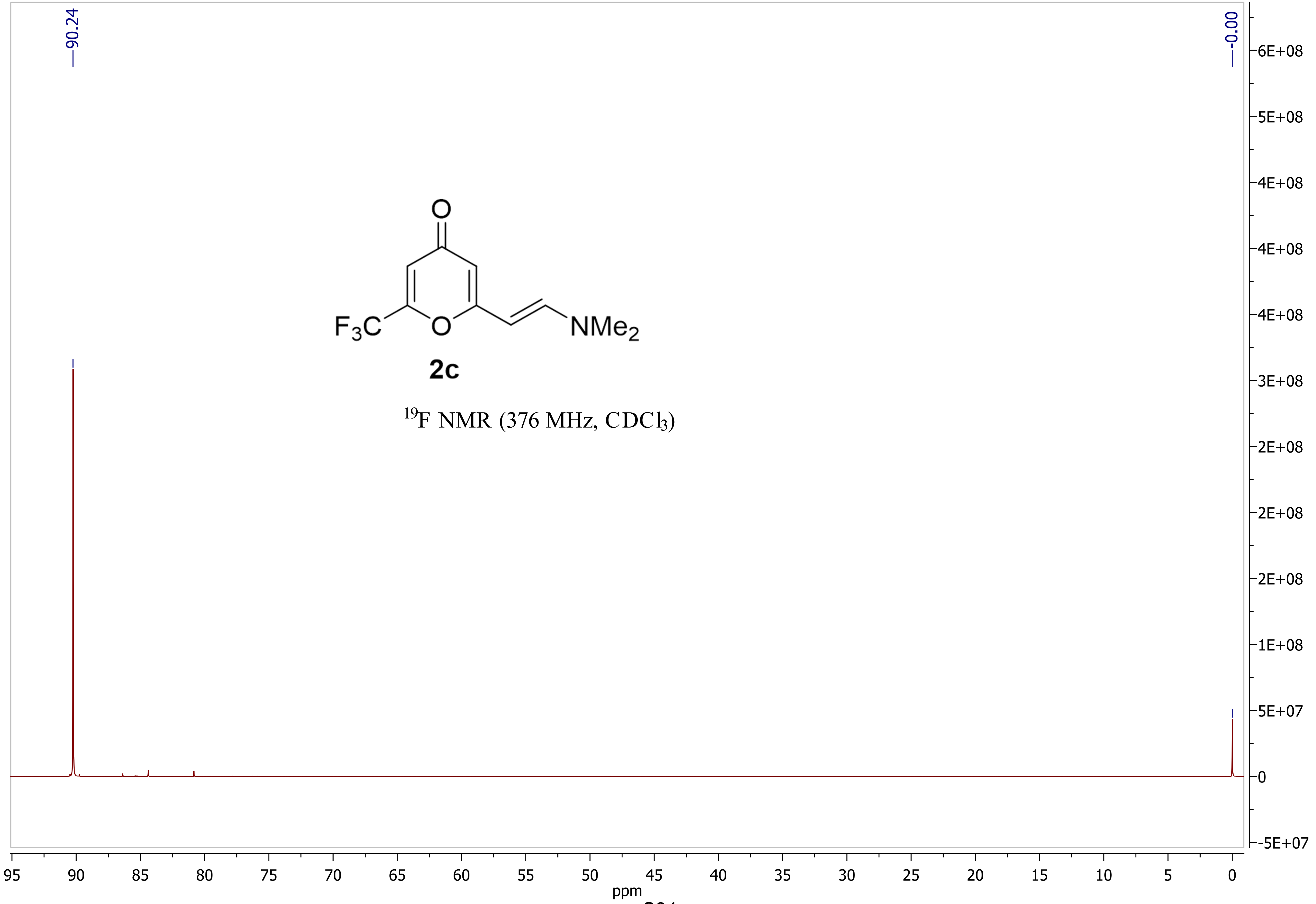

90.24
-0.00
O
F3C
O
NMe2
2c
19F NMR (376 MHz, CDCl3)
6E+08
5E+08
4E+08
4E+08
4E+08
3E+08
2E+08
2E+08
2E+08
1E+08
5E+07
0
-5E+07
95
90
85
80
75
70
65
60
55
50
45
40
35
30
25
20
15
10
5
0
ppm

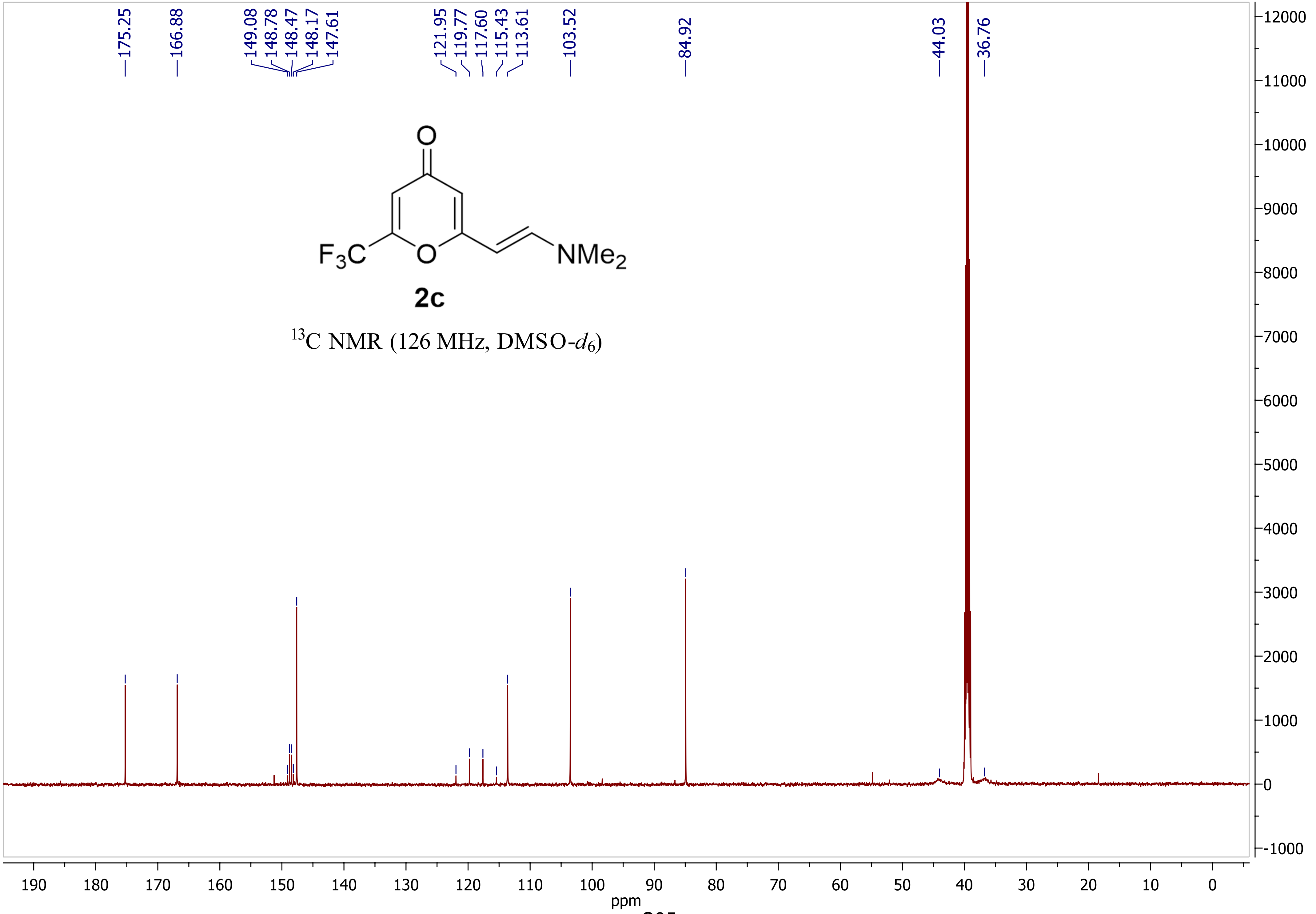
175.25
166.88
149.08
148.78
148.47
148.17
147.61
121.95
119.77
117.60
115.43
113.61
103.52
84.92
44.03
36.76
O
F3C
O
NMe2
2c
13C NMR (126 MHz, DMSO-d6)
ppm

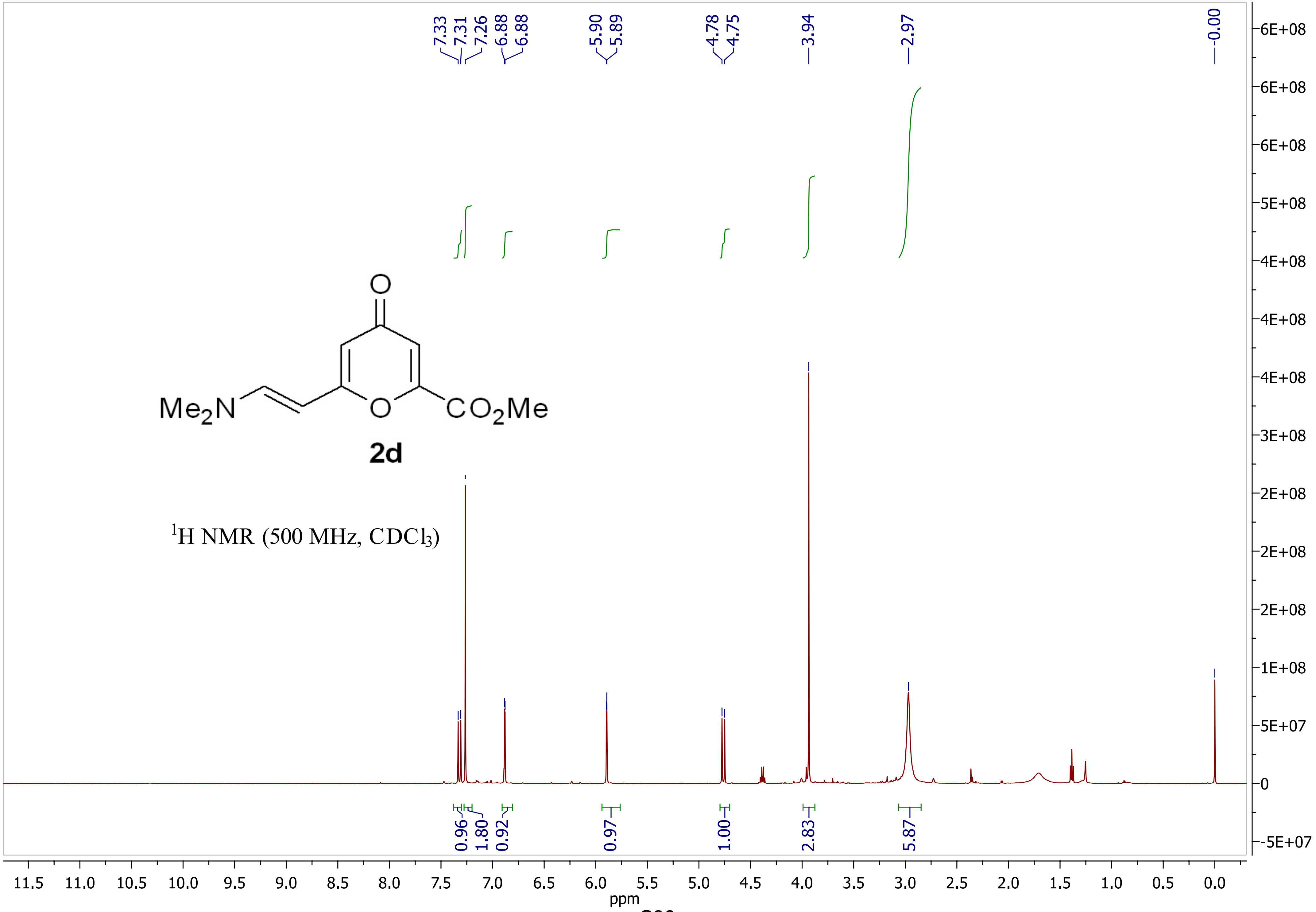

7.33
7.31
7.26
6.88
6.88
5.90
5.89
4.78
4.75
3.94
2.97
-0.00
Me2N
O
O
CO2Me
2d
1H NMR (500 MHz, CDCl3)
0.96
1.80
0.92
0.97
1.00
2.83
5.87
11.5
11.0
10.5
10.0
9.5
9.0
8.5
8.0
7.5
7.0
6.5
6.0
5.5
5.0
4.5
4.0
3.5
3.0
2.5
2.0
1.5
1.0
0.5
0.0
ppm

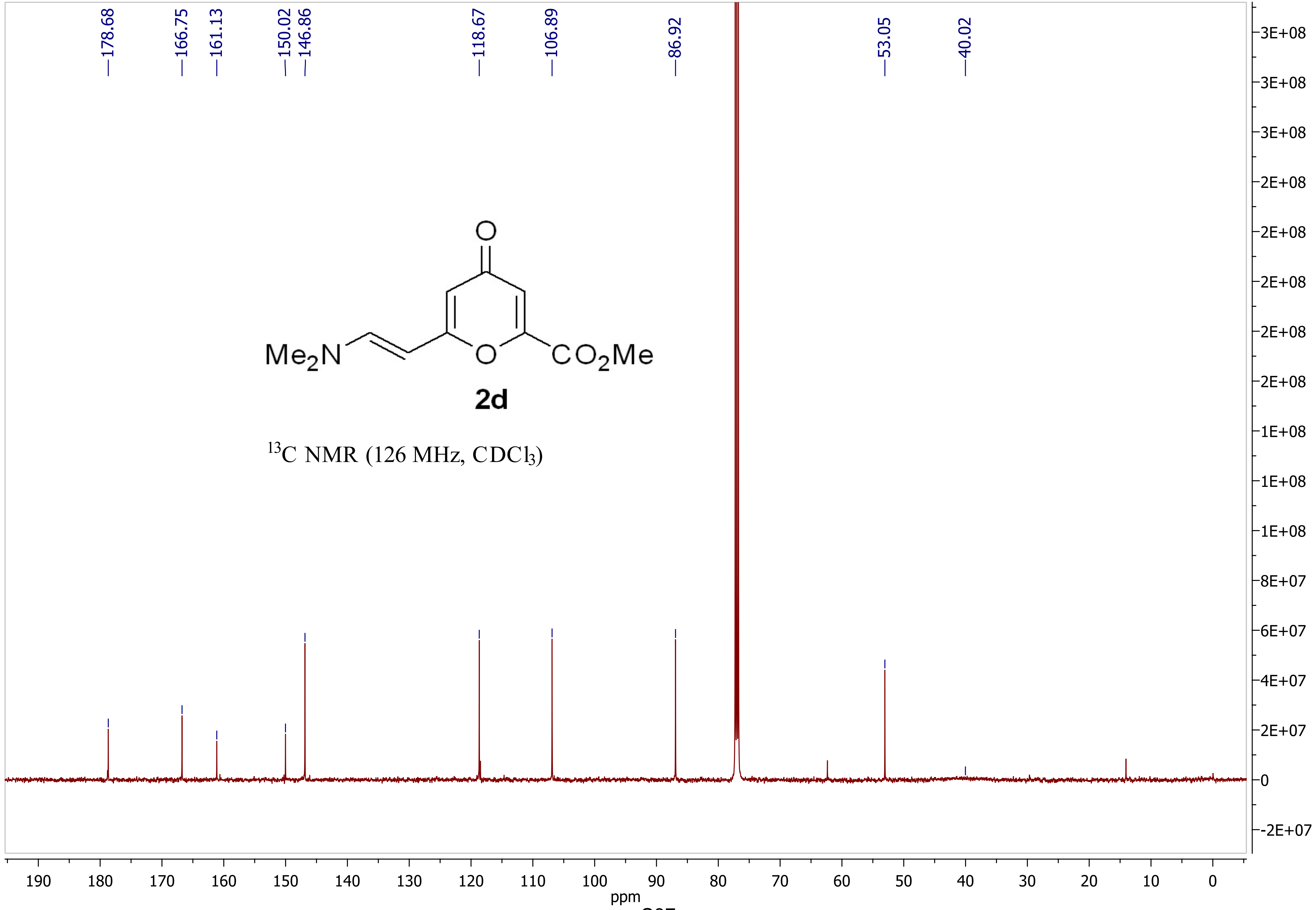
178.68
166.75
161.13
150.02
146.86
118.67
106.89
86.92
53.05
40.02
O
Me2N
O
CO2Me
2d
13C NMR (126 MHz, CDCl3)
190 180 170 160 150 140 130 120 110 100 90 80 70 60 50 40 30 20 10 0
ppm
3E+08
3E+08
3E+08
2E+08
2E+08
2E+08
2E+08
2E+08
1E+08
1E+08
1E+08
8E+07
6E+07
4E+07
2E+07
0
-2E+07

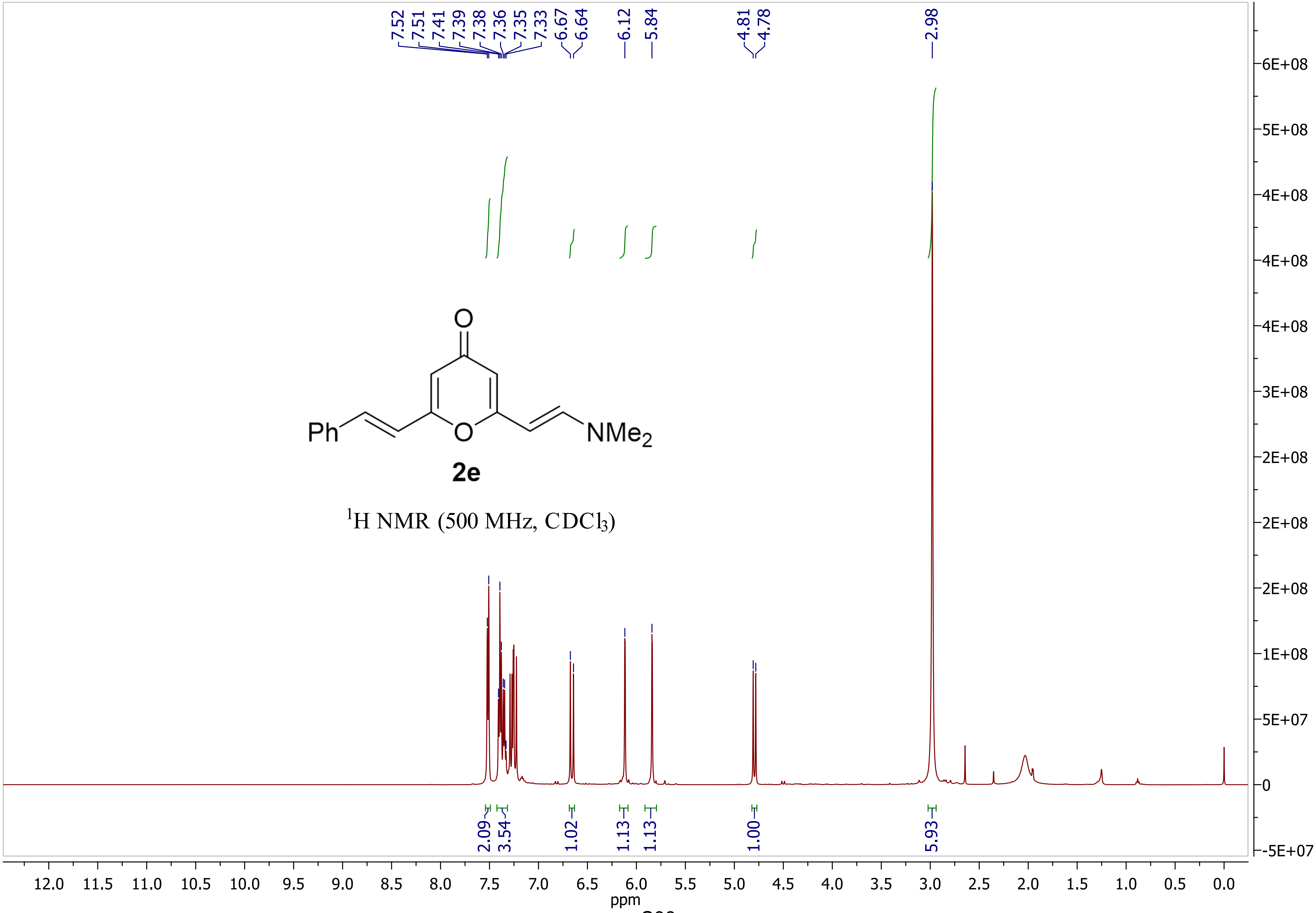

^{1}H NMR (500 MHz, $CDCl_3$)

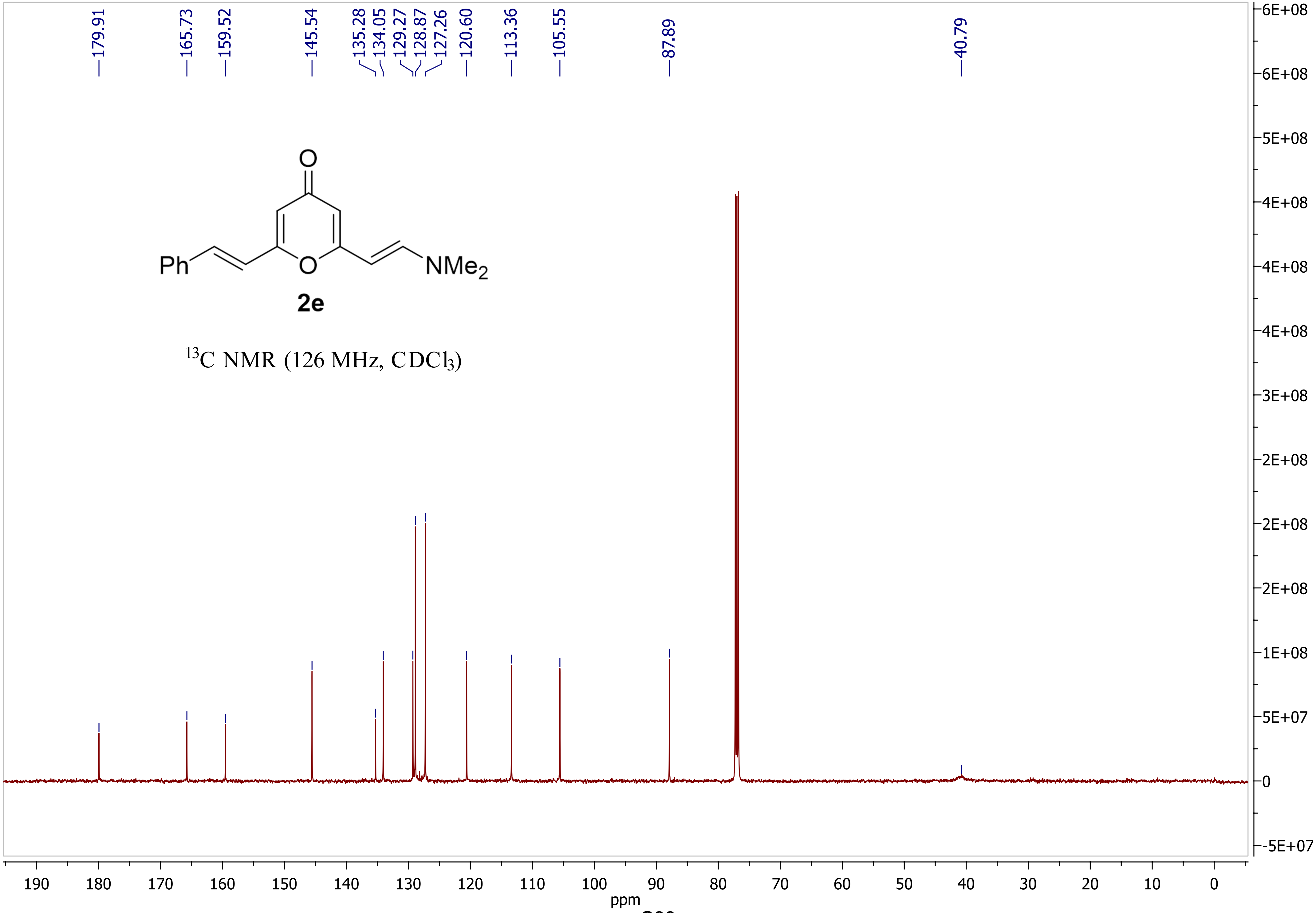
179.91
165.73
159.52
145.54
135.28
134.05
129.27
128.87
127.26
120.60
113.36
105.55
87.89
40.79
O
Ph
O
NMe2
2e
13C NMR (126 MHz, CDCl3)
6E+08
6E+08
5E+08
4E+08
4E+08
4E+08
3E+08
2E+08
2E+08
2E+08
1E+08
5E+07
0
-5E+07
190
180
170
160
150
140
130
120
110
100
90
80
70
60
50
40
30
20
10
0
ppm

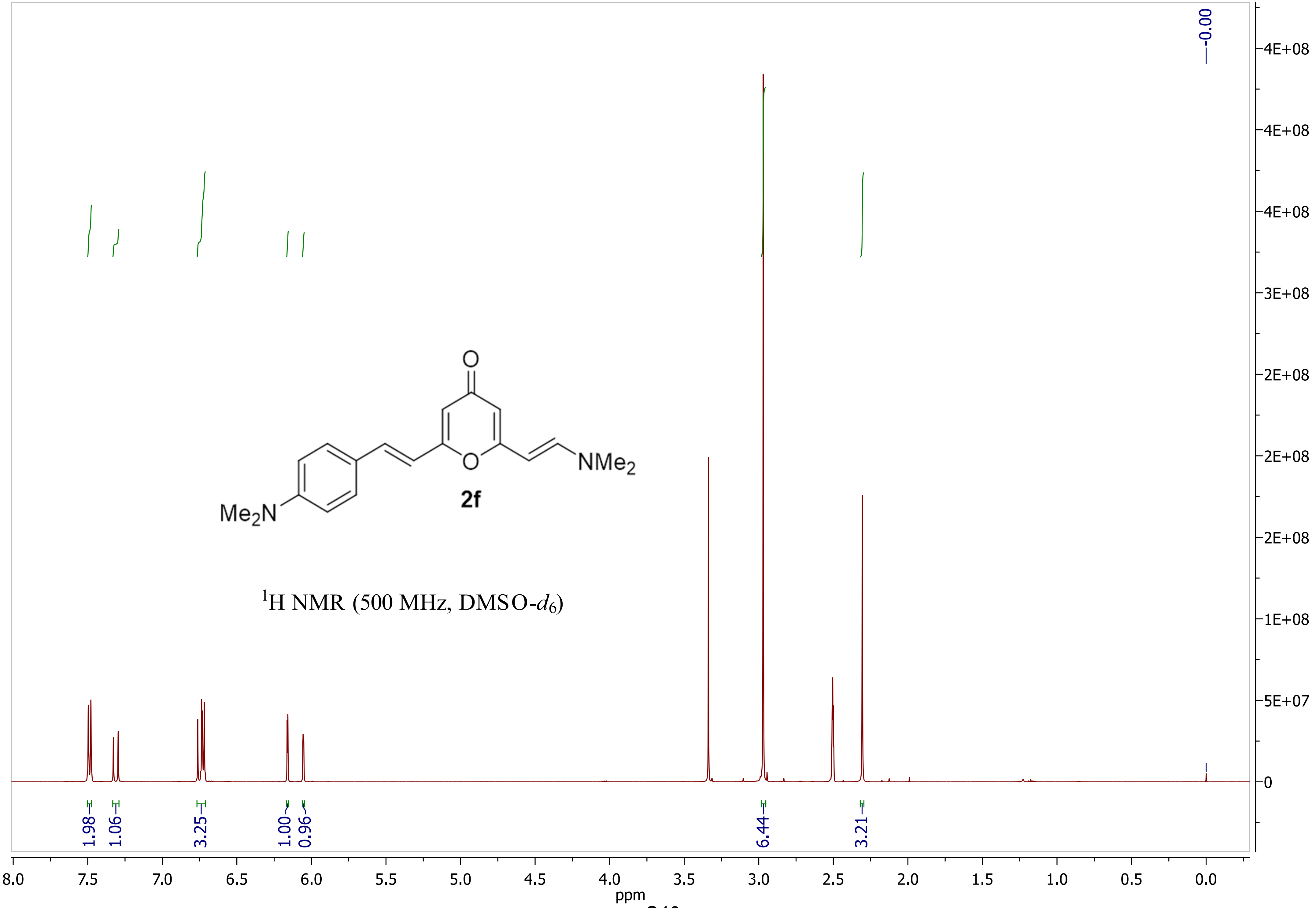

O
O
NMe2
Me2N
2f
1H NMR (500 MHz, DMSO-d6)
-0.00
1.98
1.06
3.25
1.00
0.96
6.44
3.21
4E+08
4E+08
4E+08
3E+08
2E+08
2E+08
2E+08
1E+08
5E+07
0
8.0
7.5
7.0
6.5
6.0
5.5
5.0
4.5
4.0
3.5
3.0
2.5
2.0
1.5
1.0
0.5
0.0
ppm

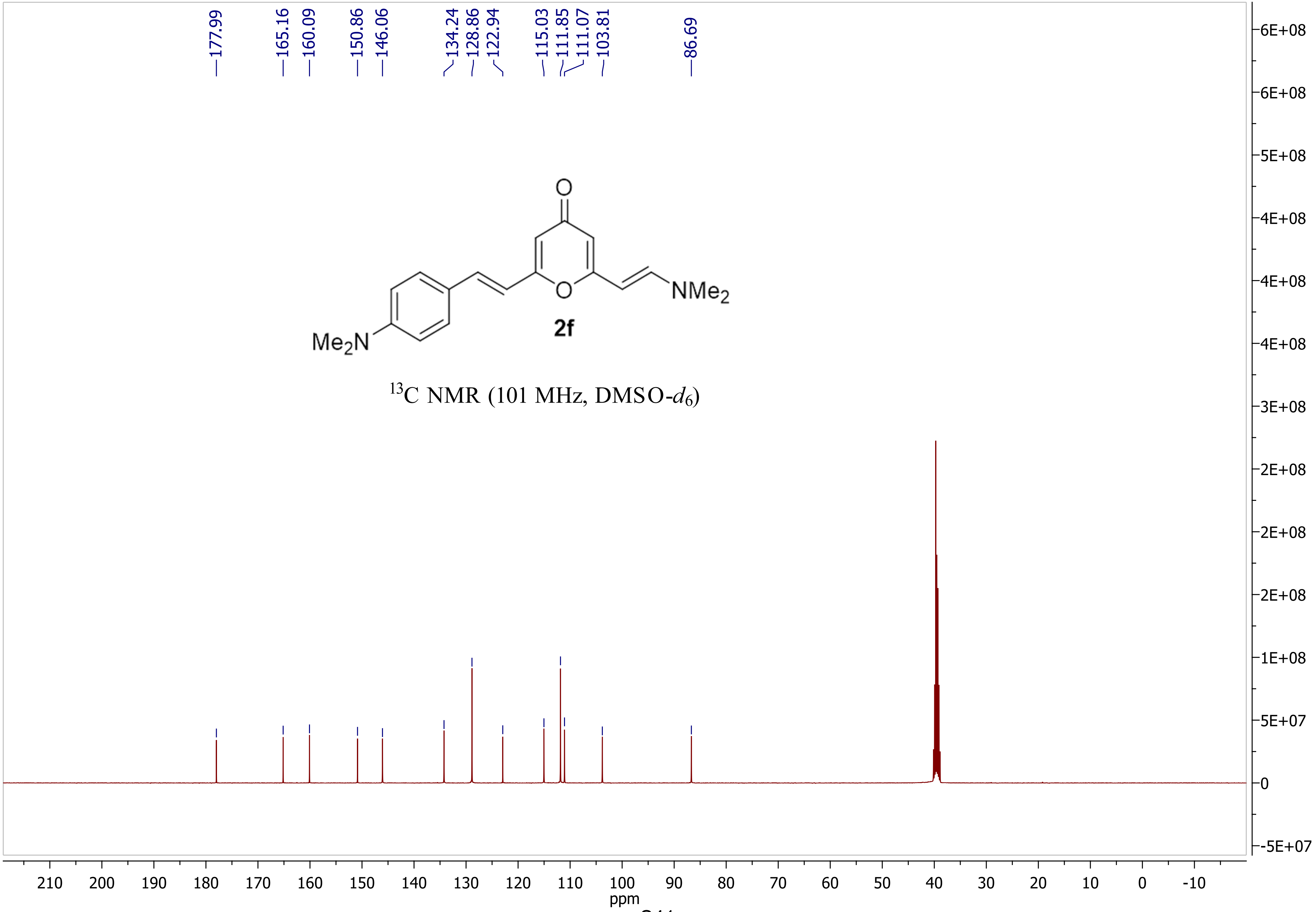
177.99
165.16
160.09
150.86
146.06
134.24
128.86
122.94
115.03
111.85
111.07
103.81
86.69
O
O
NMe2
Me2N
2f
13C NMR (101 MHz, DMSO-d6)
6E+08
6E+08
5E+08
4E+08
4E+08
4E+08
3E+08
2E+08
2E+08
2E+08
1E+08
5E+07
0
-5E+07
210 200 190 180 170 160 150 140 130 120 110 100 90 80 70 60 50 40 30 20 10 0 -10
ppm

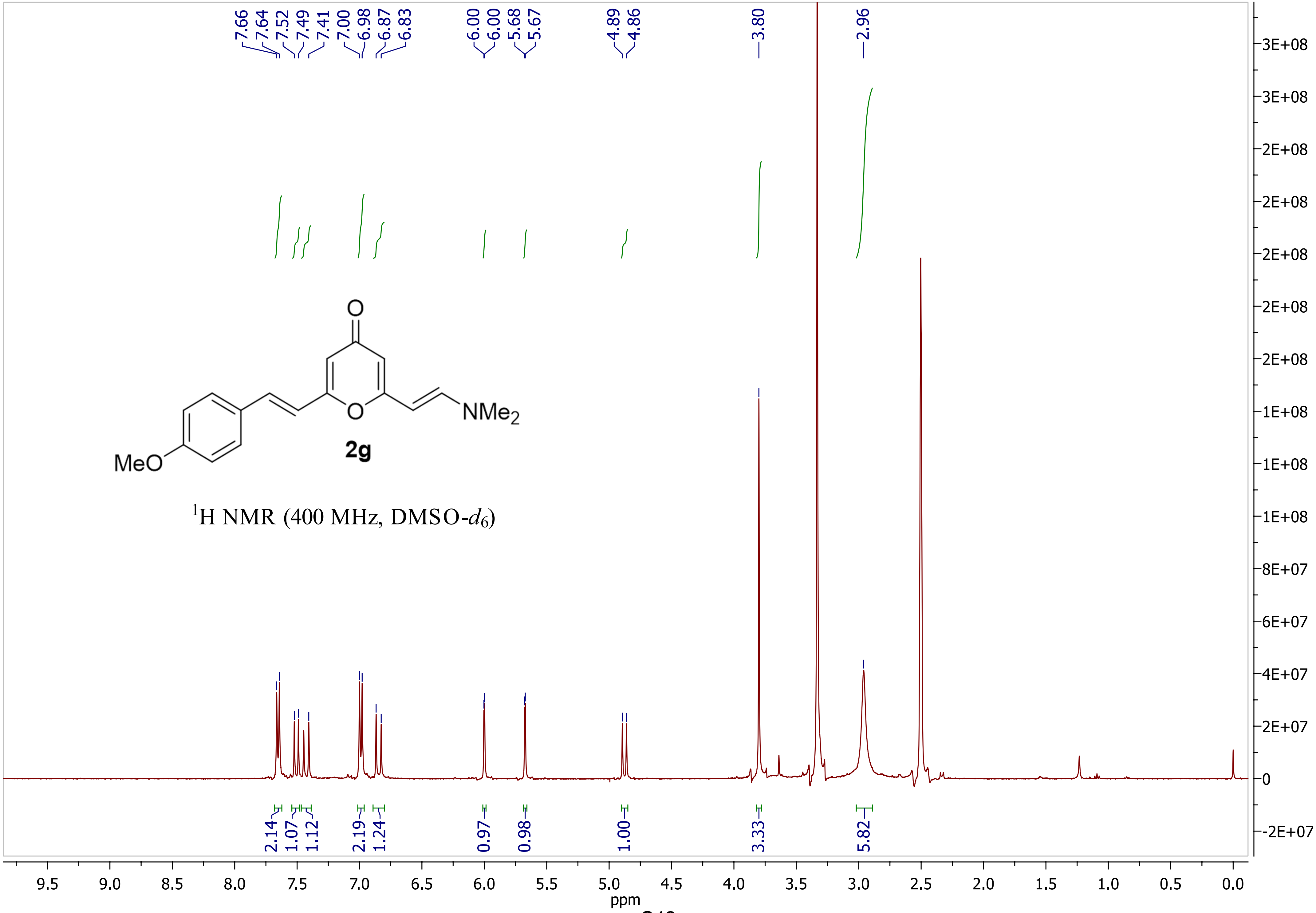
7.66 7.64 7.52 7.49 7.41 7.00 6.98 6.87 6.83
6.00 6.00 5.68 5.67
4.89 4.86
3.80
2.96
O
O
NMe2
MeO
2g
^{1}H NMR (400 MHz, DMSO-d_6)
2.14 1.07 1.12 2.19 1.24 0.97 0.98 1.00 3.33 5.82
9.5 9.0 8.5 8.0 7.5 7.0 6.5 6.0 5.5 5.0 4.5 4.0 3.5 3.0 2.5 2.0 1.5 1.0 0.5 0.0
ppm
3E+08 3E+08 2E+08 2E+08 2E+08 2E+08 2E+08 1E+08 1E+08 1E+08 8E+07 6E+07 4E+07 2E+07 0 -2E+07

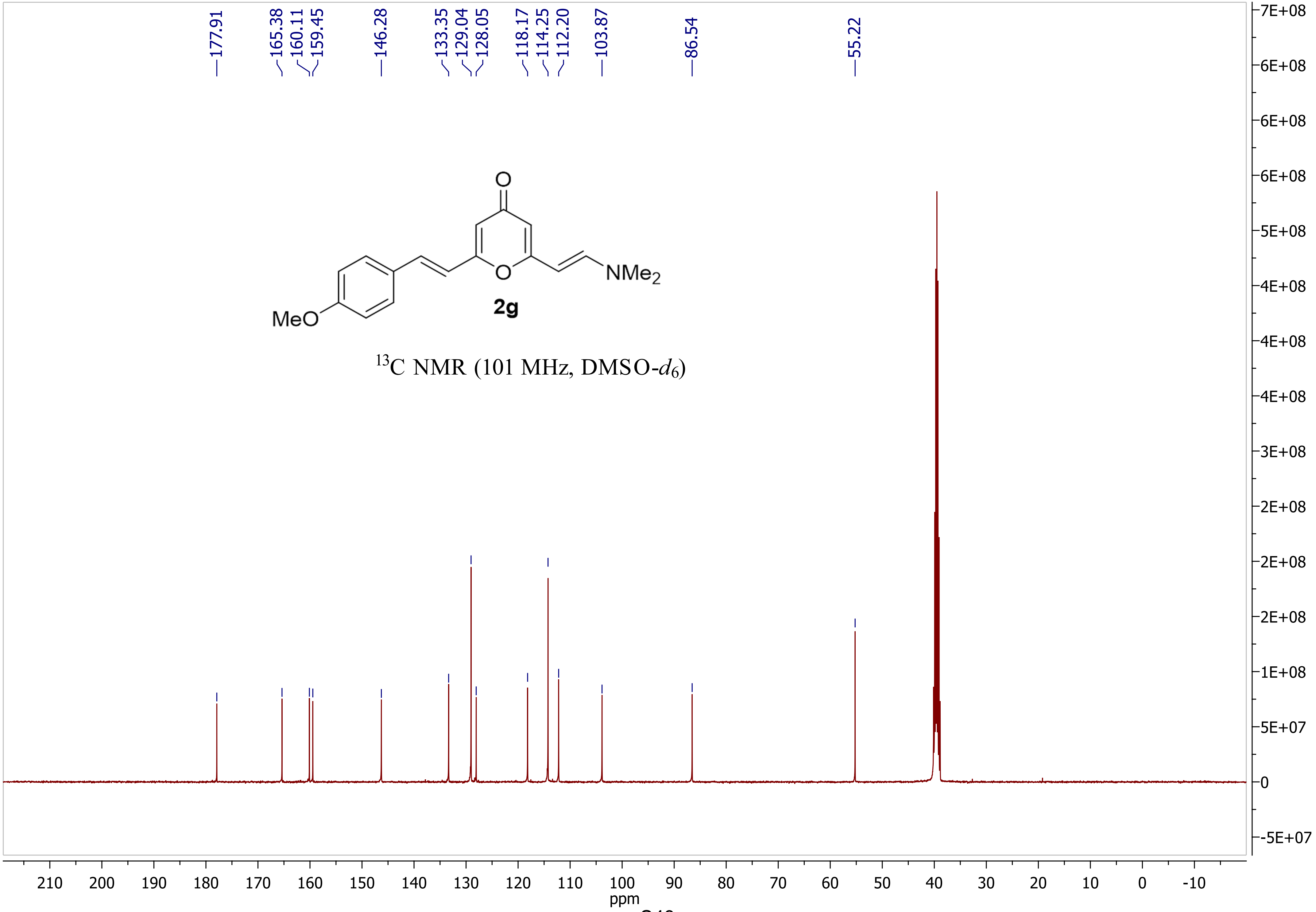
177.91
165.38
160.11
159.45
146.28
133.35
129.04
128.05
118.17
114.25
112.20
103.87
86.54
55.22
O
NMe2
MeO
2g
13C NMR (101 MHz, DMSO-d6)
ppm
210 200 190 180 170 160 150 140 130 120 110 100 90 80 70 60 50 40 30 20 10 0 -10
7E+08 6E+08 6E+08 6E+08 5E+08 4E+08 4E+08 4E+08 3E+08 2E+08 2E+08 2E+08 1E+08 5E+07 0 -5E+07

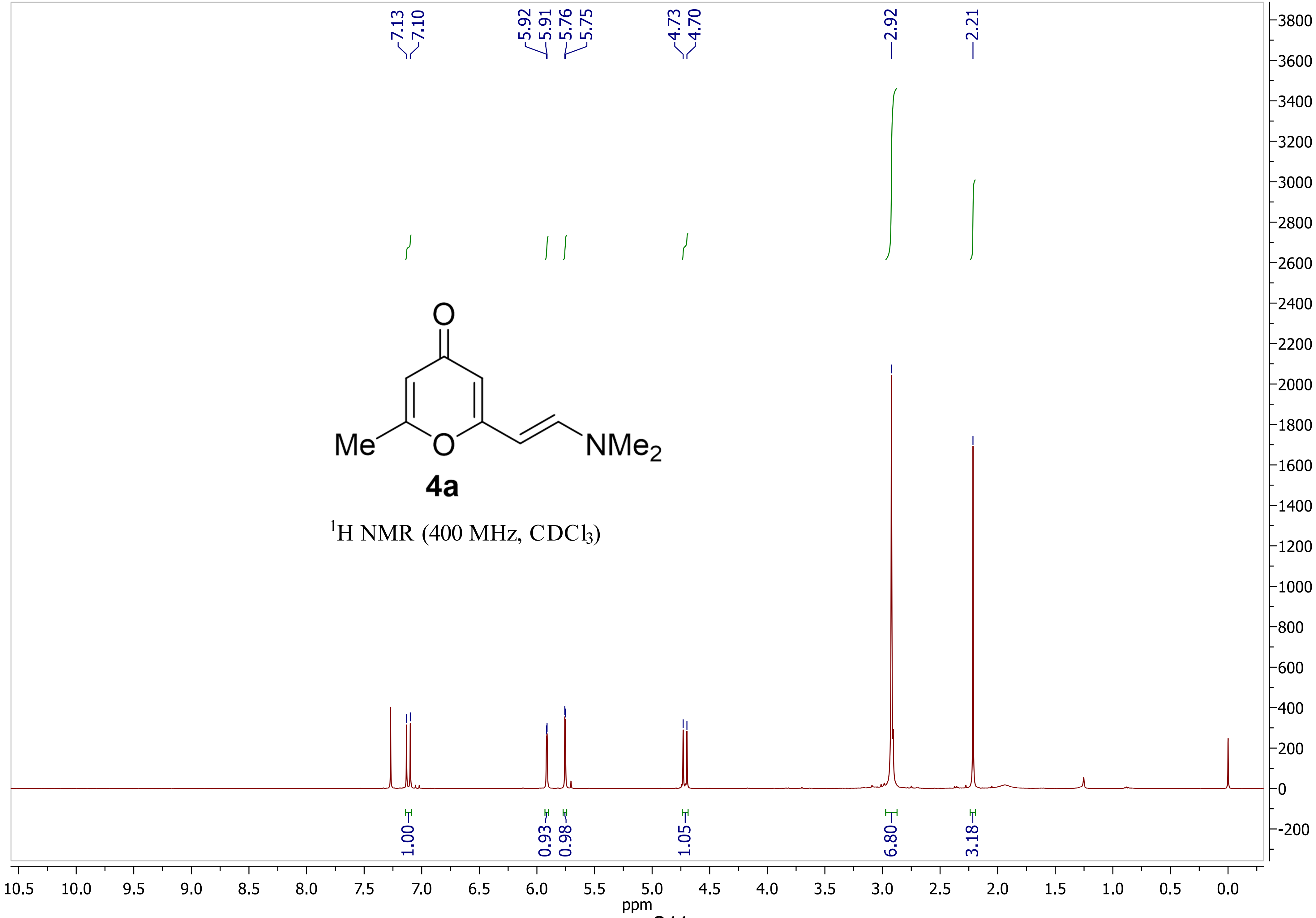
7.13
7.10
5.92
5.91
5.76
5.75
4.73
4.70
2.92
2.21
Me
O
O
NMe2
4a
1H NMR (400 MHz, CDCl3)
1.00
0.93
0.98
1.05
6.80
3.18
ppm

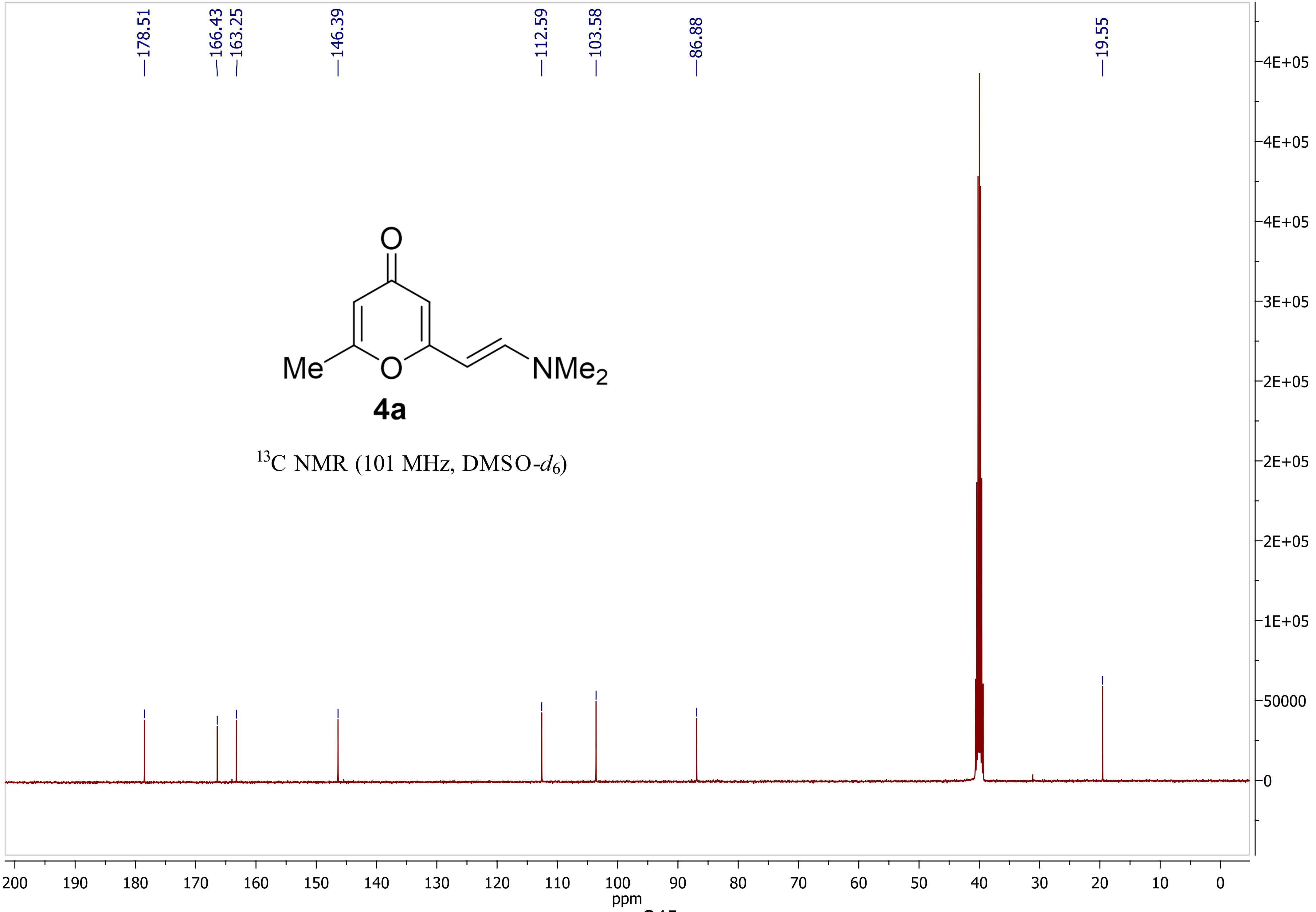

178.51
166.43
163.25
146.39
112.59
103.58
86.88
19.55
O
Me
O
NMe$_2$
4a
^{13}C NMR (101 MHz, DMSO-d_6)
4E+05
4E+05
4E+05
3E+05
2E+05
2E+05
2E+05
1E+05
50000
0
200 190 180 170 160 150 140 130 120 110 100 90 80 70 60 50 40 30 20 10 0
ppm

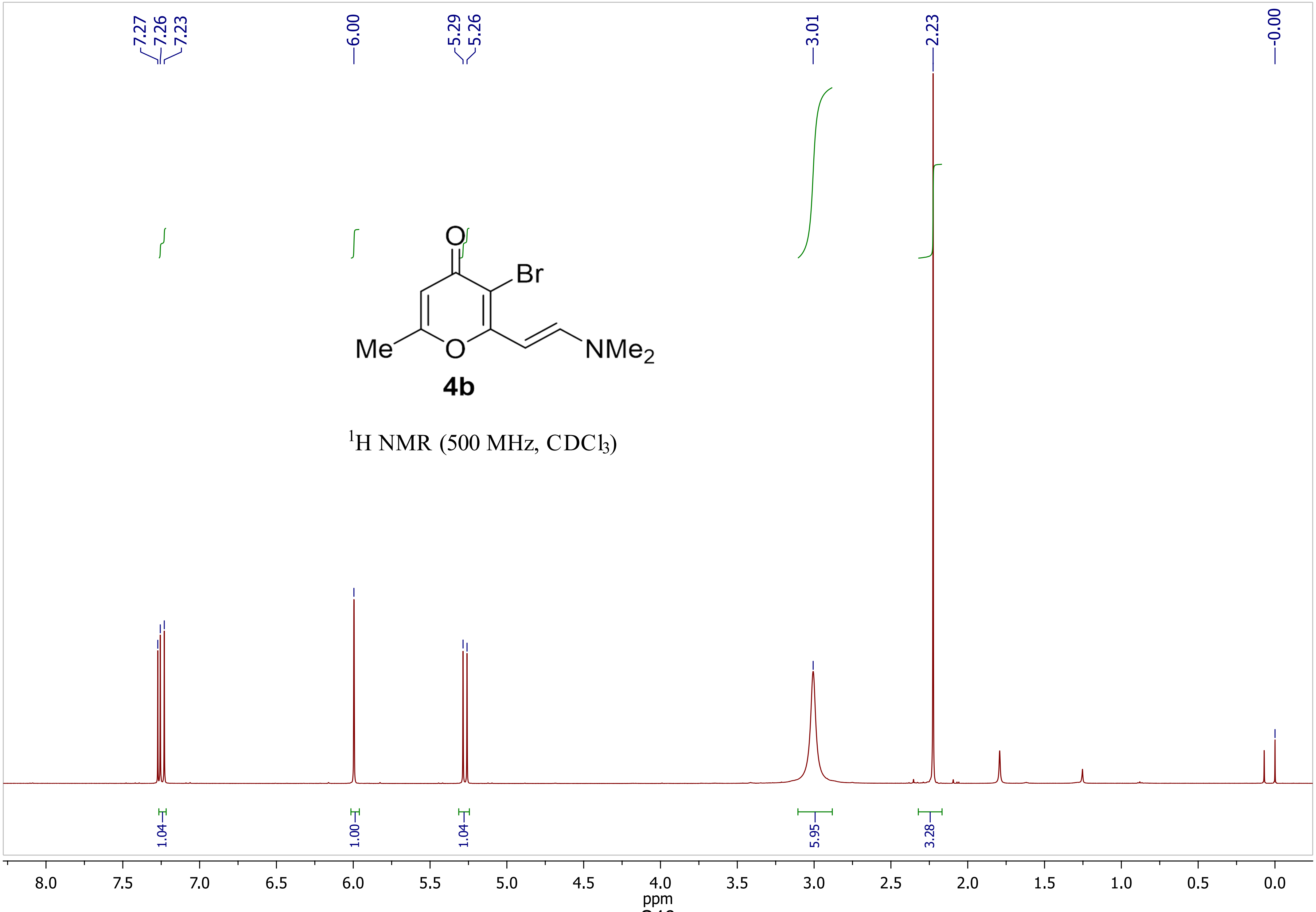

7.27
7.26
7.23
6.00
5.29
5.26
3.01
2.23
-0.00
O
Br
Me
O
NMe2
4b
1H NMR (500 MHz, CDCl3)
1.04
1.00
1.04
5.95
3.28
8.0
7.5
7.0
6.5
6.0
5.5
5.0
4.5
4.0
3.5
3.0
2.5
2.0
1.5
1.0
0.5
0.0
ppm

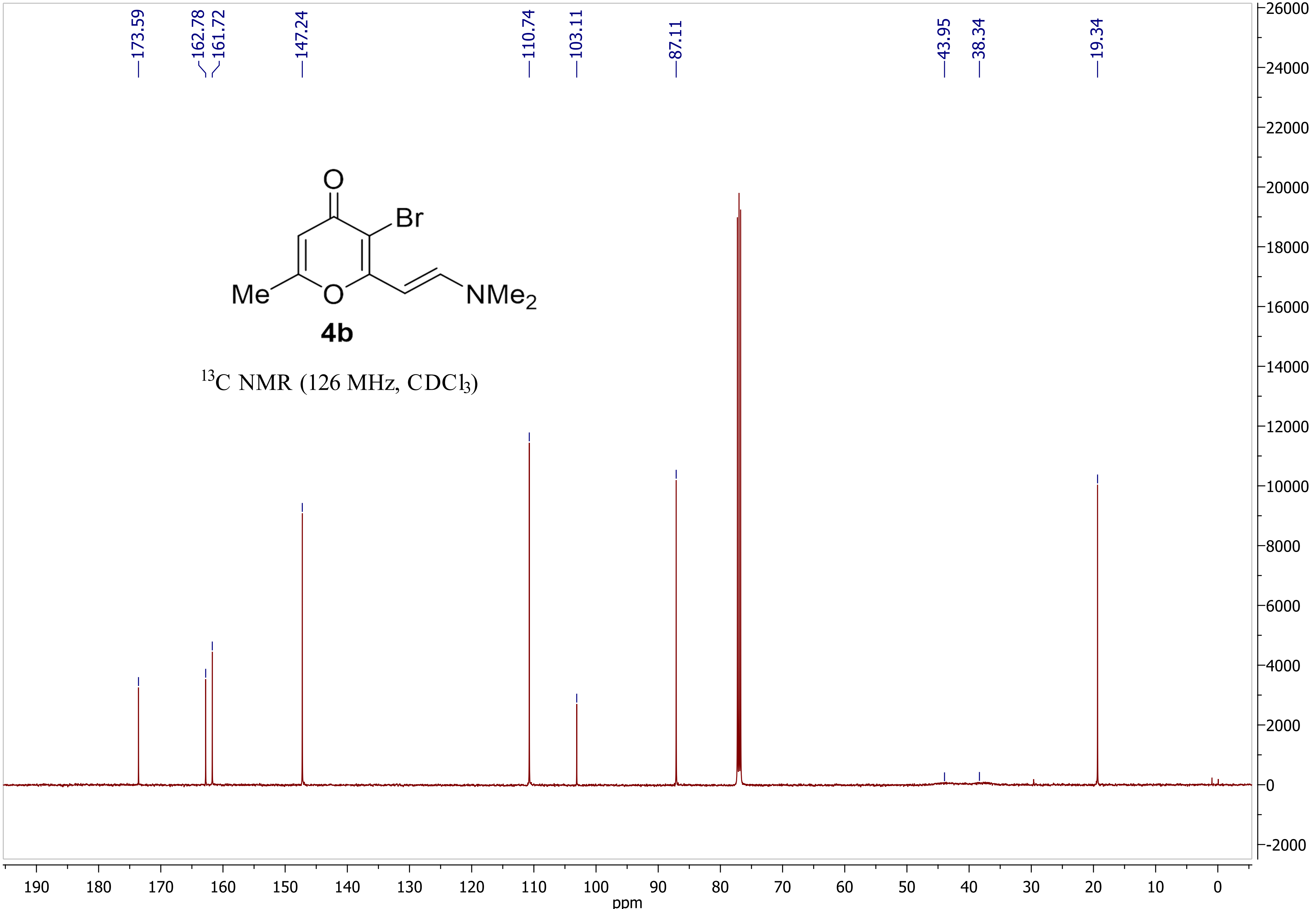
173.59
162.78
161.72
147.24
110.74
103.11
87.11
43.95
38.34
19.34
O
Br
Me
O
NMe2
4b
13C NMR (126 MHz, CDCl3)
ppm

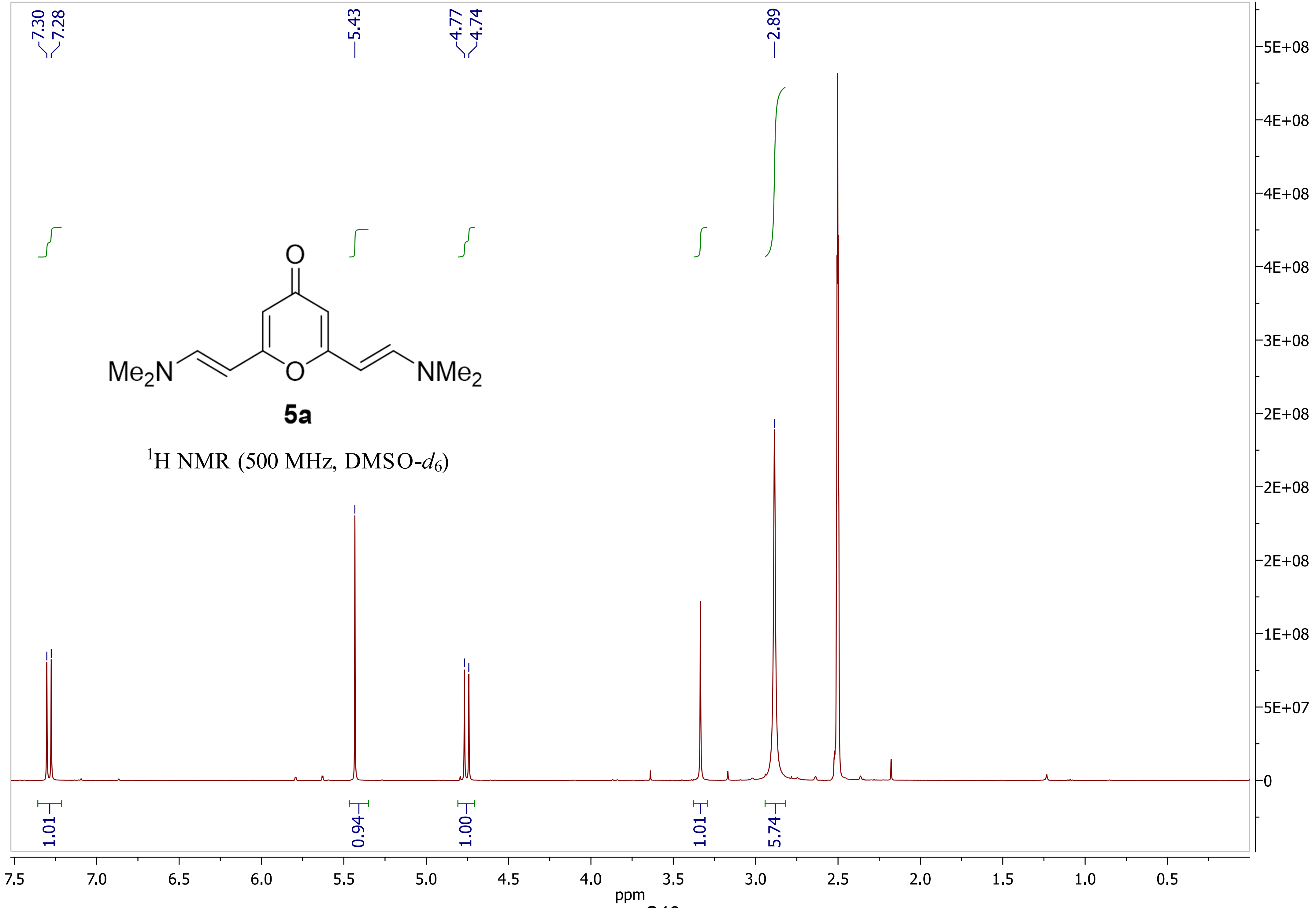
7.30
7.28
5.43
4.77
4.74
2.89
Me2N
O
O
NMe2
5a
1H NMR (500 MHz, DMSO-d6)
1.01
0.94
1.00
1.01
5.74
5E+08
4E+08
4E+08
4E+08
3E+08
2E+08
2E+08
2E+08
1E+08
5E+07
0
7.5
7.0
6.5
6.0
5.5
5.0
4.5
4.0
3.5
3.0
2.5
2.0
1.5
1.0
0.5
ppm

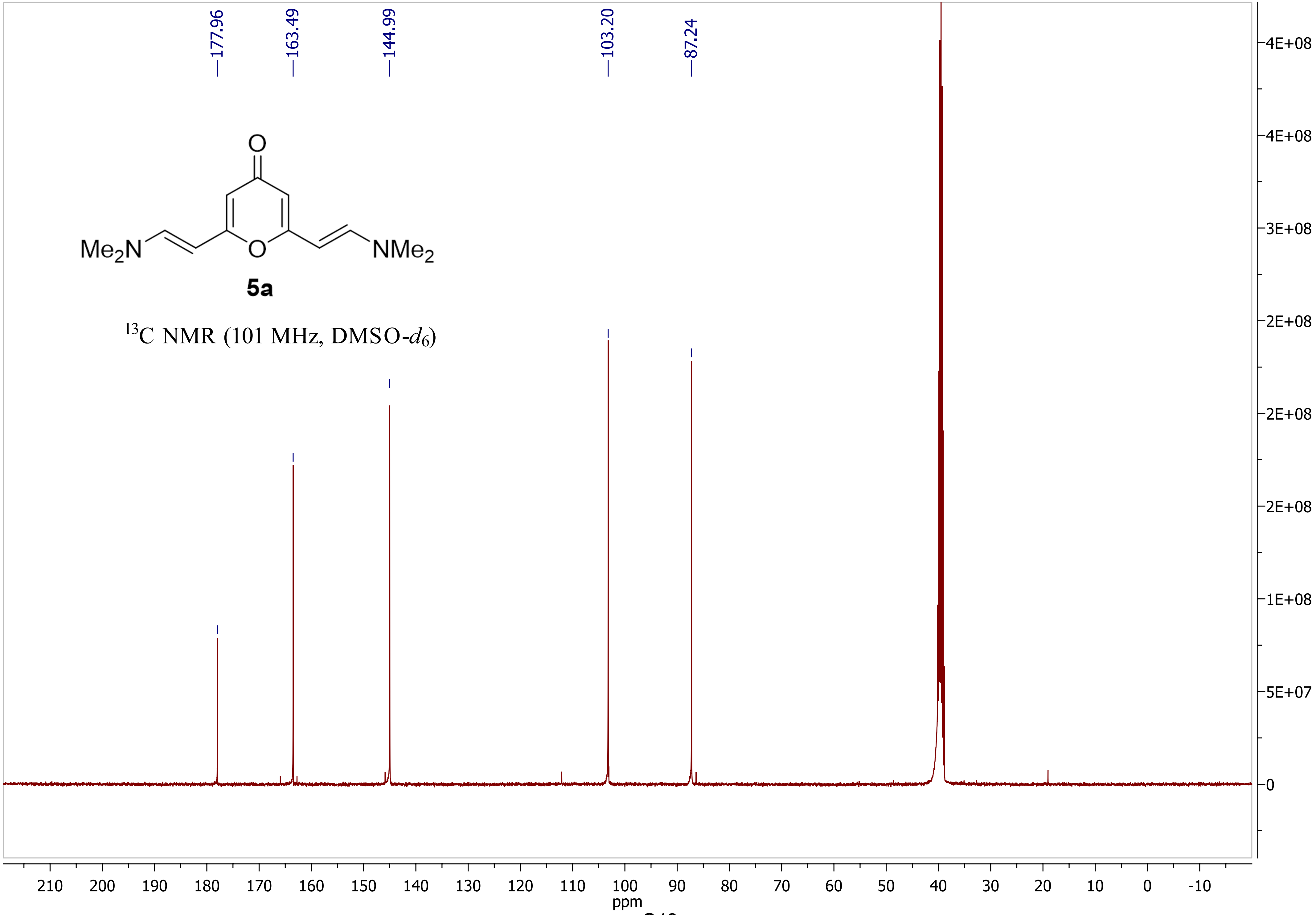
177.96
163.49
144.99
103.20
87.24
Me2N
O
O
NMe2
5a
13C NMR (101 MHz, DMSO-d6)
4E+08
4E+08
3E+08
2E+08
2E+08
2E+08
1E+08
5E+07
0
210
200
190
180
170
160
150
140
130
120
110
100
90
80
70
60
50
40
30
20
10
0
-10
ppm

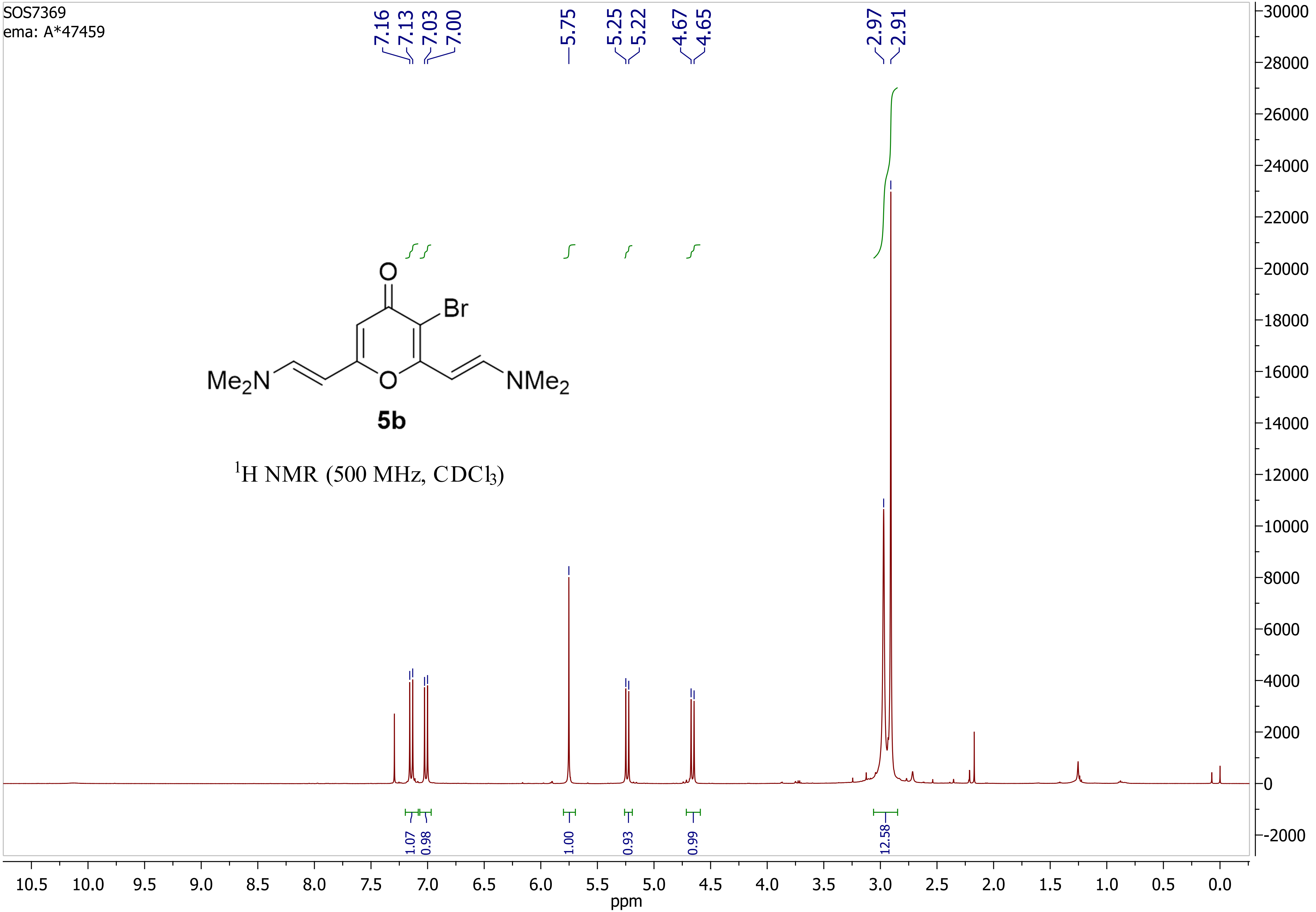

SOS7369
ema: A*47459
7.16
7.13
7.03
7.00
5.75
5.25
5.22
4.67
4.65
2.97
2.91
Br
O
O
Me2N
NMe2
5b
1H NMR (500 MHz, CDCl3)
1.07
0.98
1.00
0.93
0.99
12.58
10.5 10.0 9.5 9.0 8.5 8.0 7.5 7.0 6.5 6.0 5.5 5.0 4.5 4.0 3.5 3.0 2.5 2.0 1.5 1.0 0.5 0.0
ppm
30000 28000 26000 24000 22000 20000 18000 16000 14000 12000 10000 8000 6000 4000 2000 0 -2000

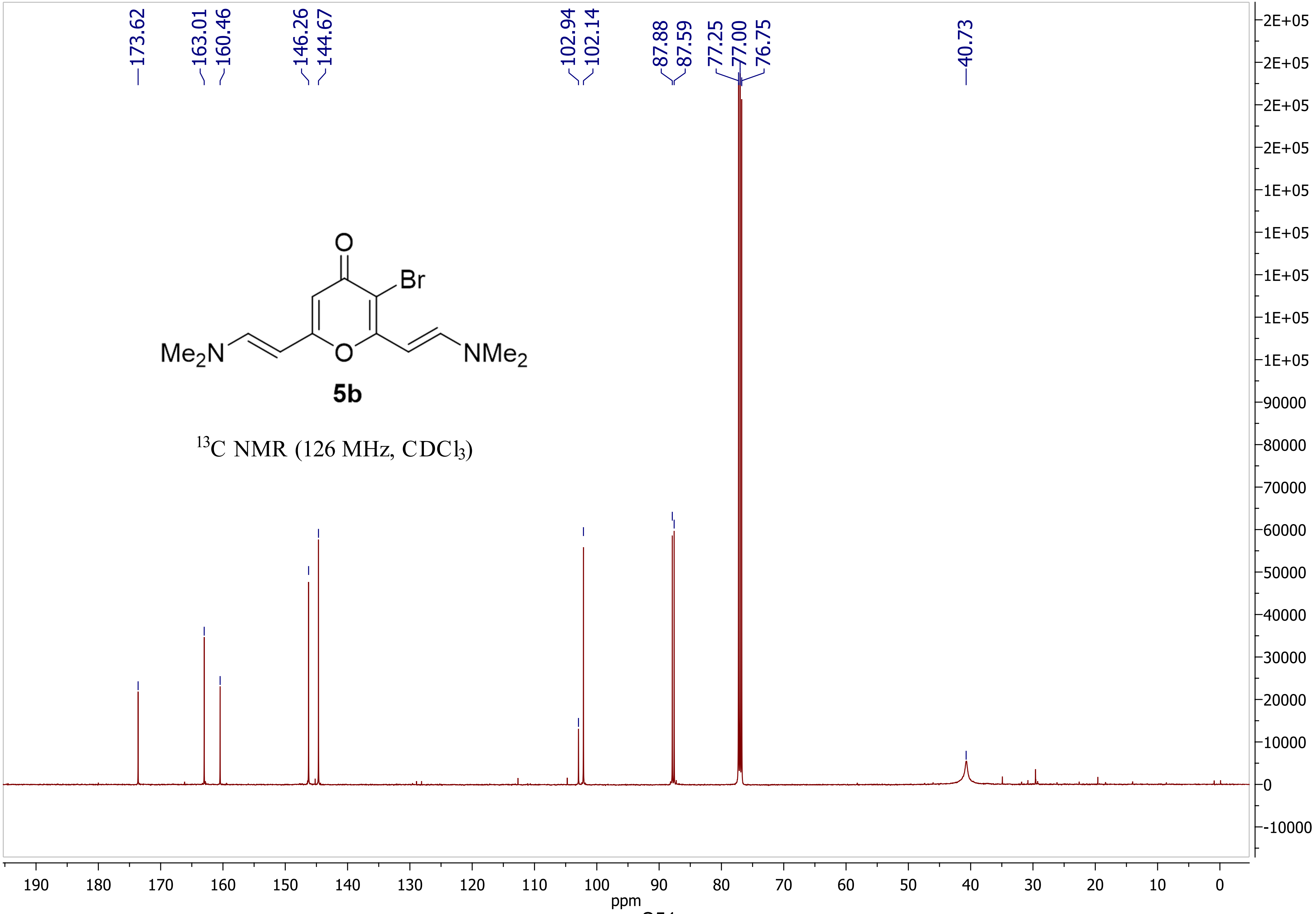
173.62
163.01
160.46
146.26
144.67
102.94
102.14
87.88
87.59
77.25
77.00
76.75
40.73
Br
O
Me2N
NMe2
5b
13C NMR (126 MHz, CDCl3)
ppm

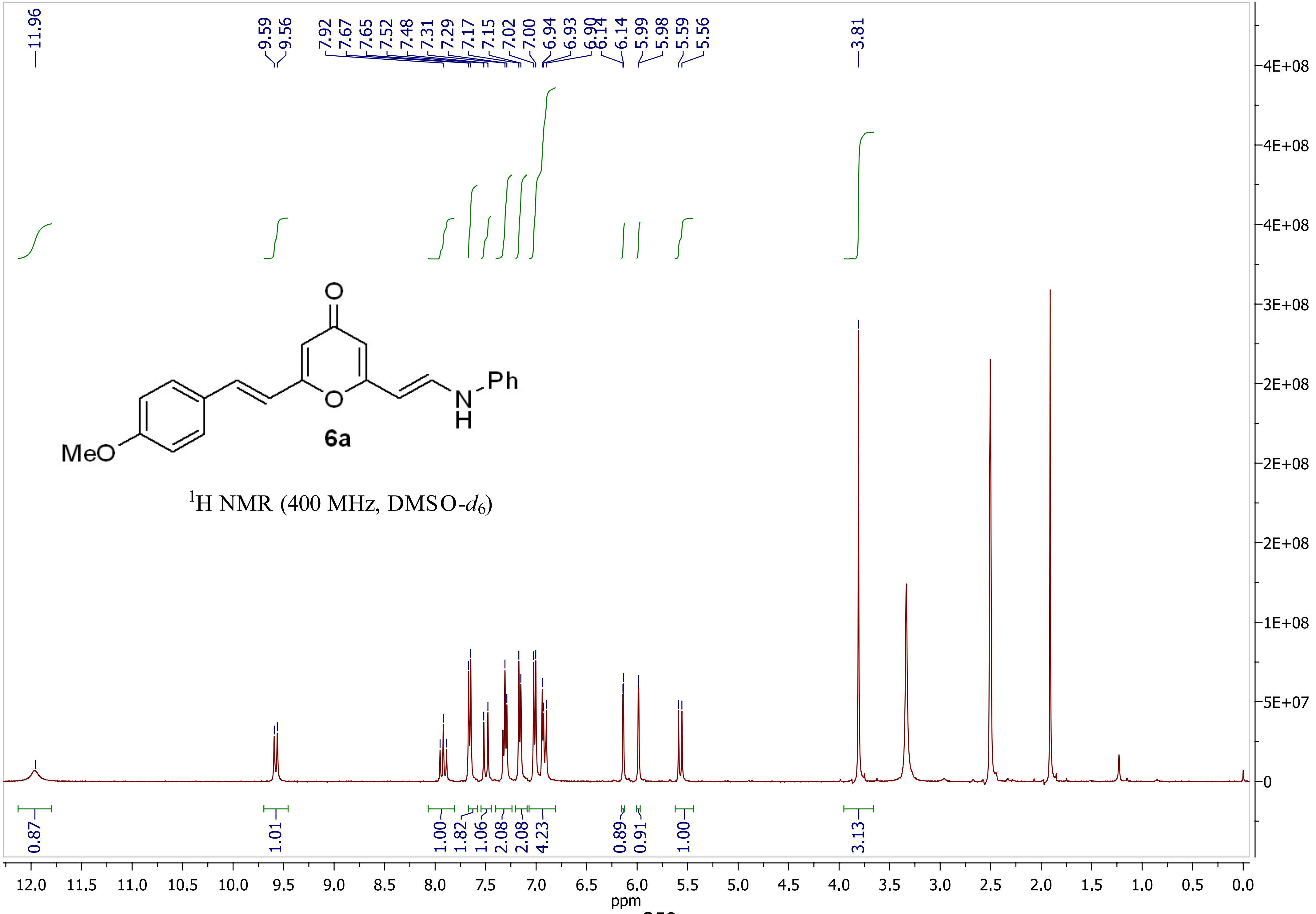
11.96
9.59
9.56
7.92
7.67
7.65
7.52
7.48
7.31
7.29
7.17
7.15
7.02
7.00
6.94
6.93
6.90
6.14
6.14
5.99
5.98
5.59
5.56
3.81
O
O
N
H
Ph
MeO
6a
^{1}H NMR (400 MHz, DMSO-d_6)
0.87
1.01
1.00
1.82
1.06
2.08
2.08
4.23
0.89
0.91
1.00
3.13
4E+08
4E+08
4E+08
3E+08
2E+08
2E+08
2E+08
1E+08
5E+07
0
12.0 11.5 11.0 10.5 10.0 9.5 9.0 8.5 8.0 7.5 7.0 6.5 6.0 5.5 5.0 4.5 4.0 3.5 3.0 2.5 2.0 1.5 1.0 0.5 0.0
ppm

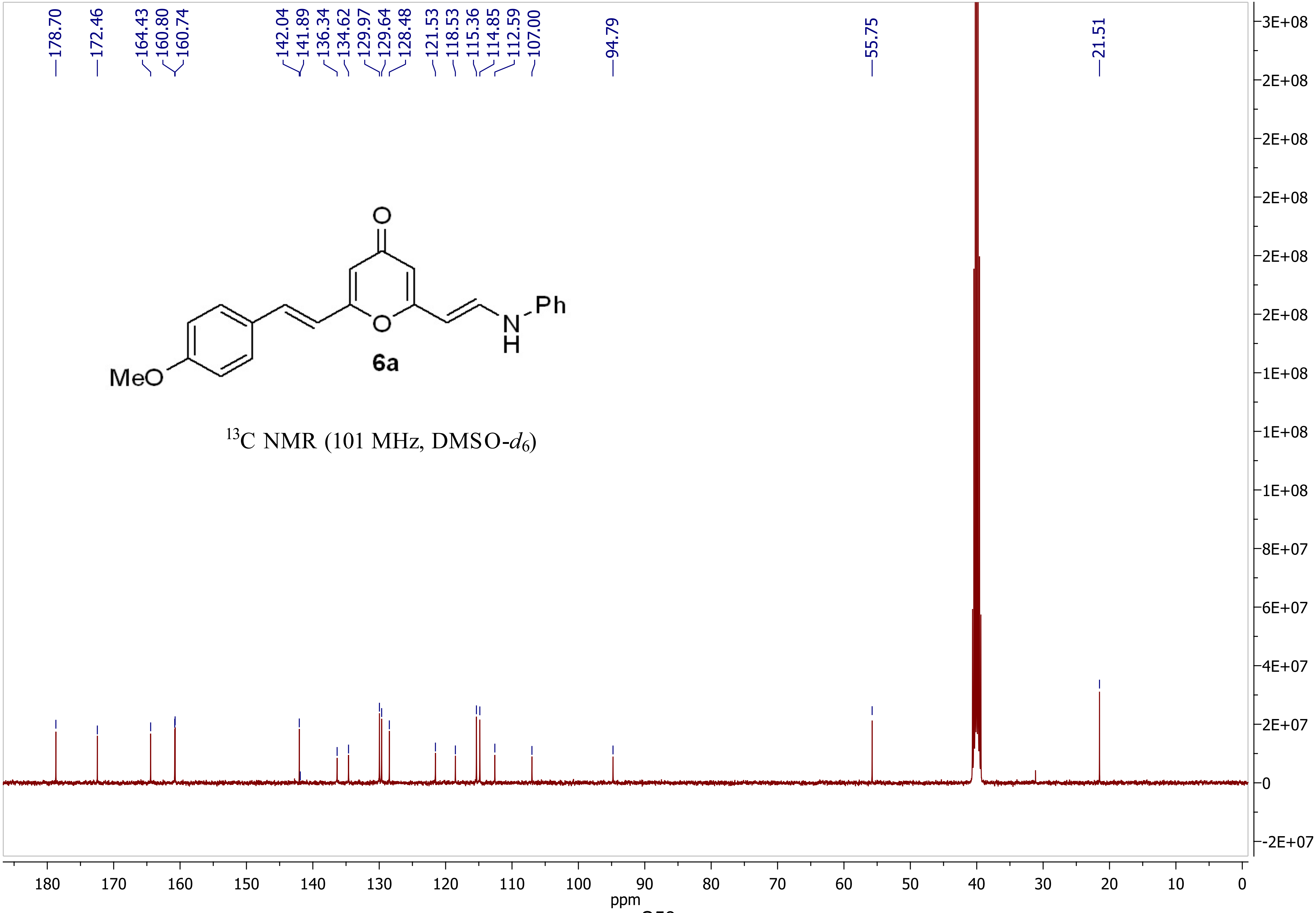

^{13}C NMR (101 MHz, DMSO-d_6)

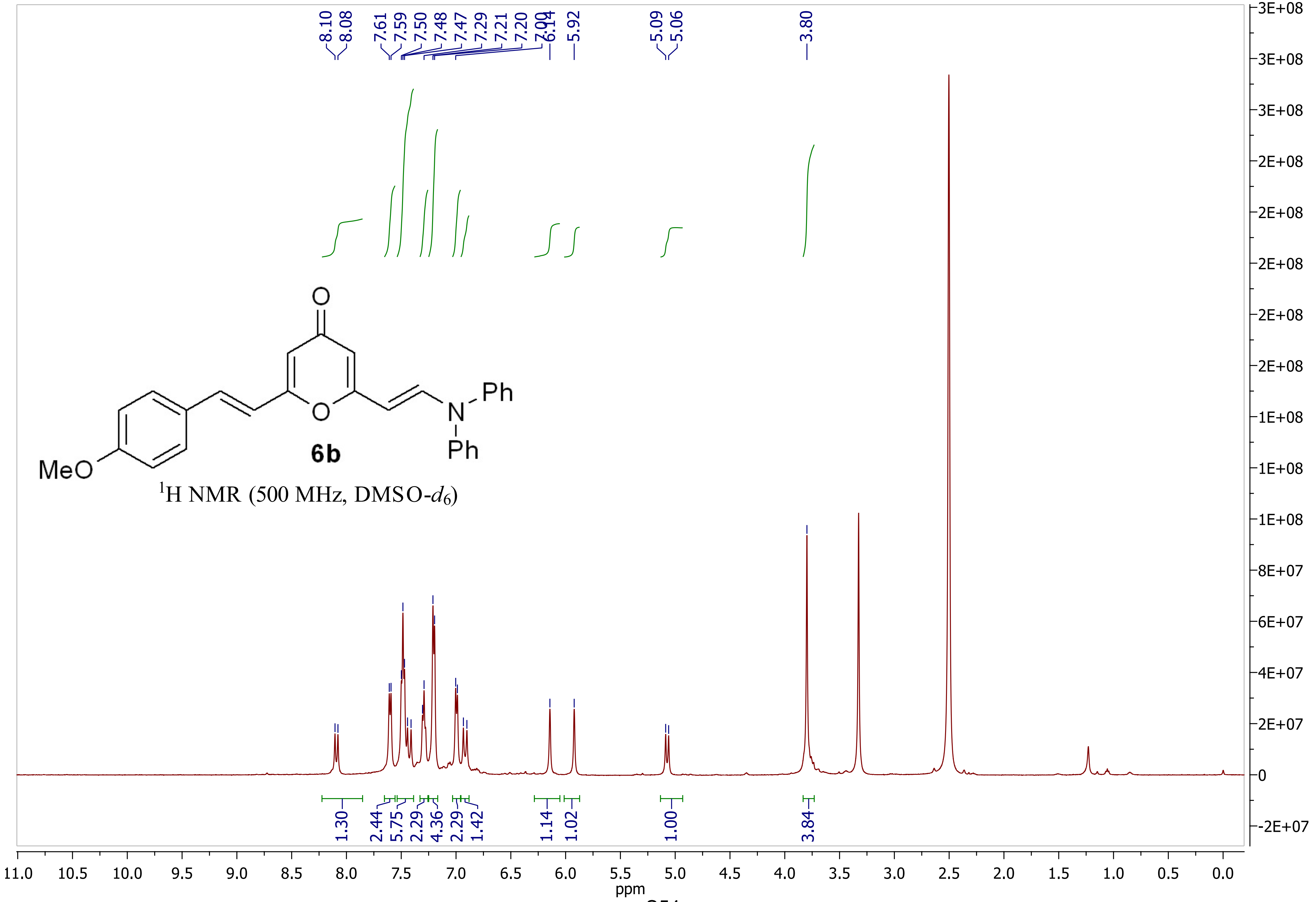

^{1}H NMR (500 MHz, DMSO-d_6)

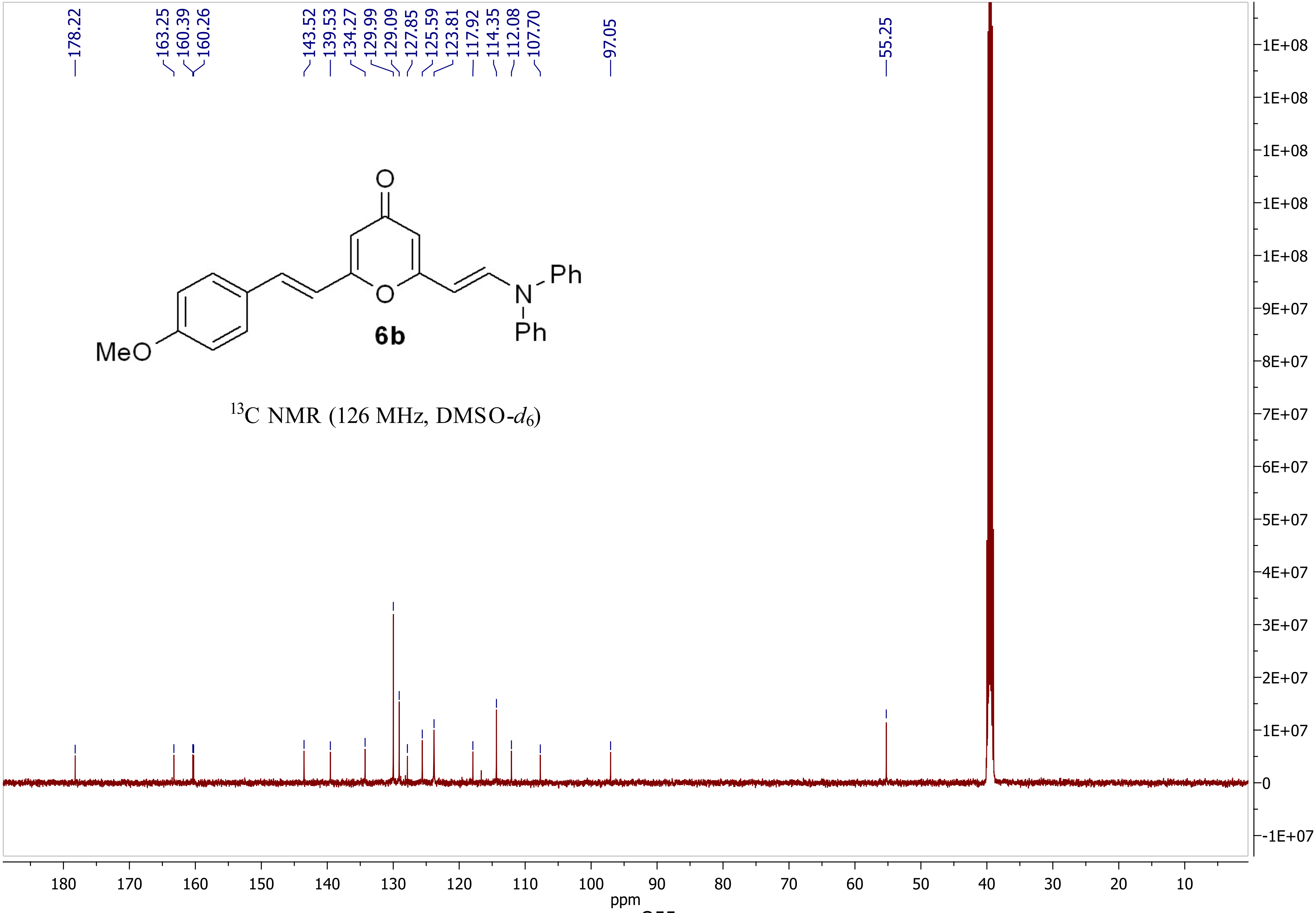

^{13}C NMR (126 MHz, DMSO-d_6)

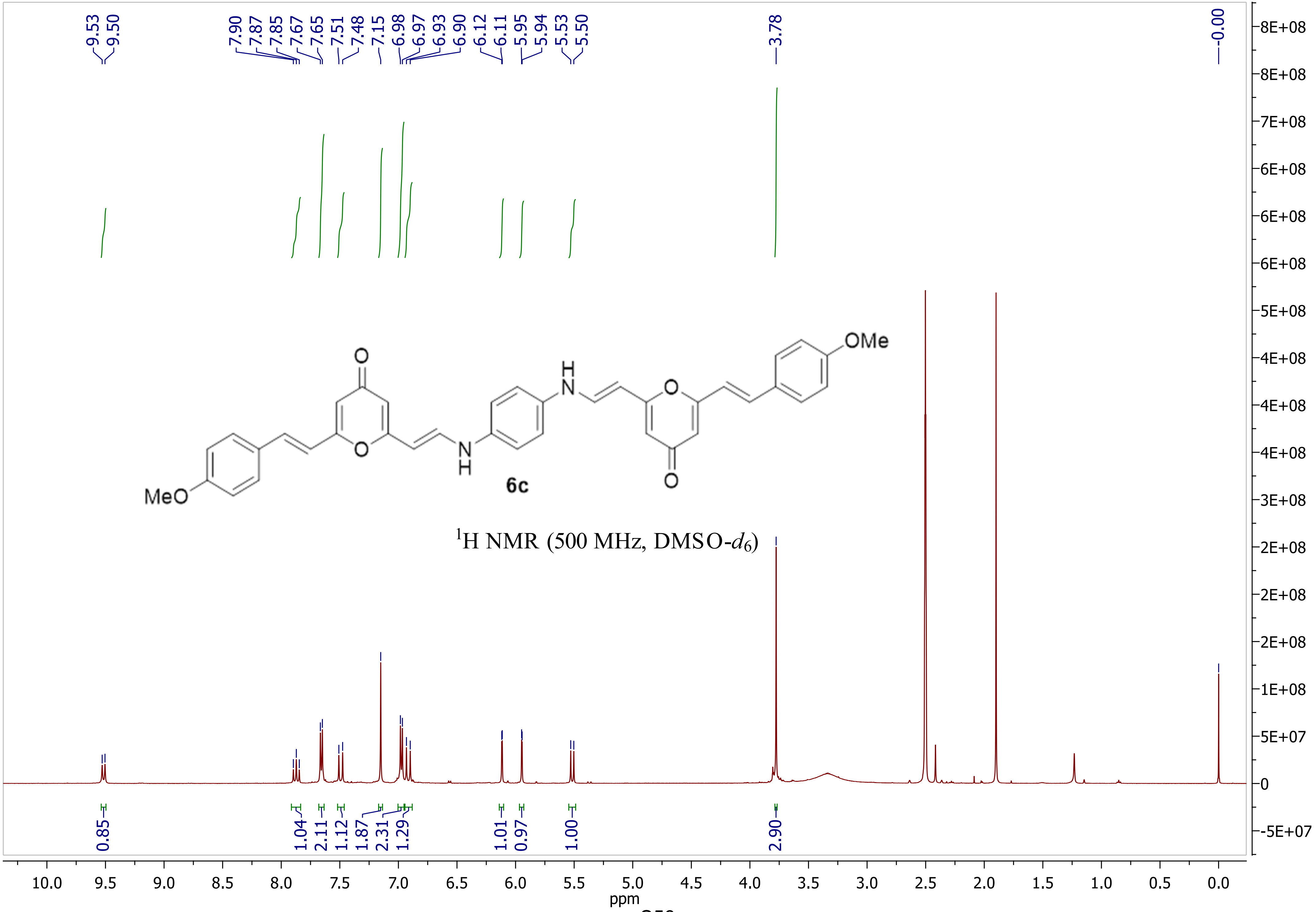

9.53
9.50
7.90
7.87
7.85
7.67
7.65
7.51
7.48
7.15
6.98
6.97
6.93
6.90
6.12
6.11
5.95
5.94
5.53
5.50
3.78
0.00
OMe
MeO
6c
^{1}H NMR (500 MHz, DMSO-d_6)
0.85
1.04
2.11
1.12
1.87
2.31
1.29
1.01
0.97
1.00
2.90
ppm

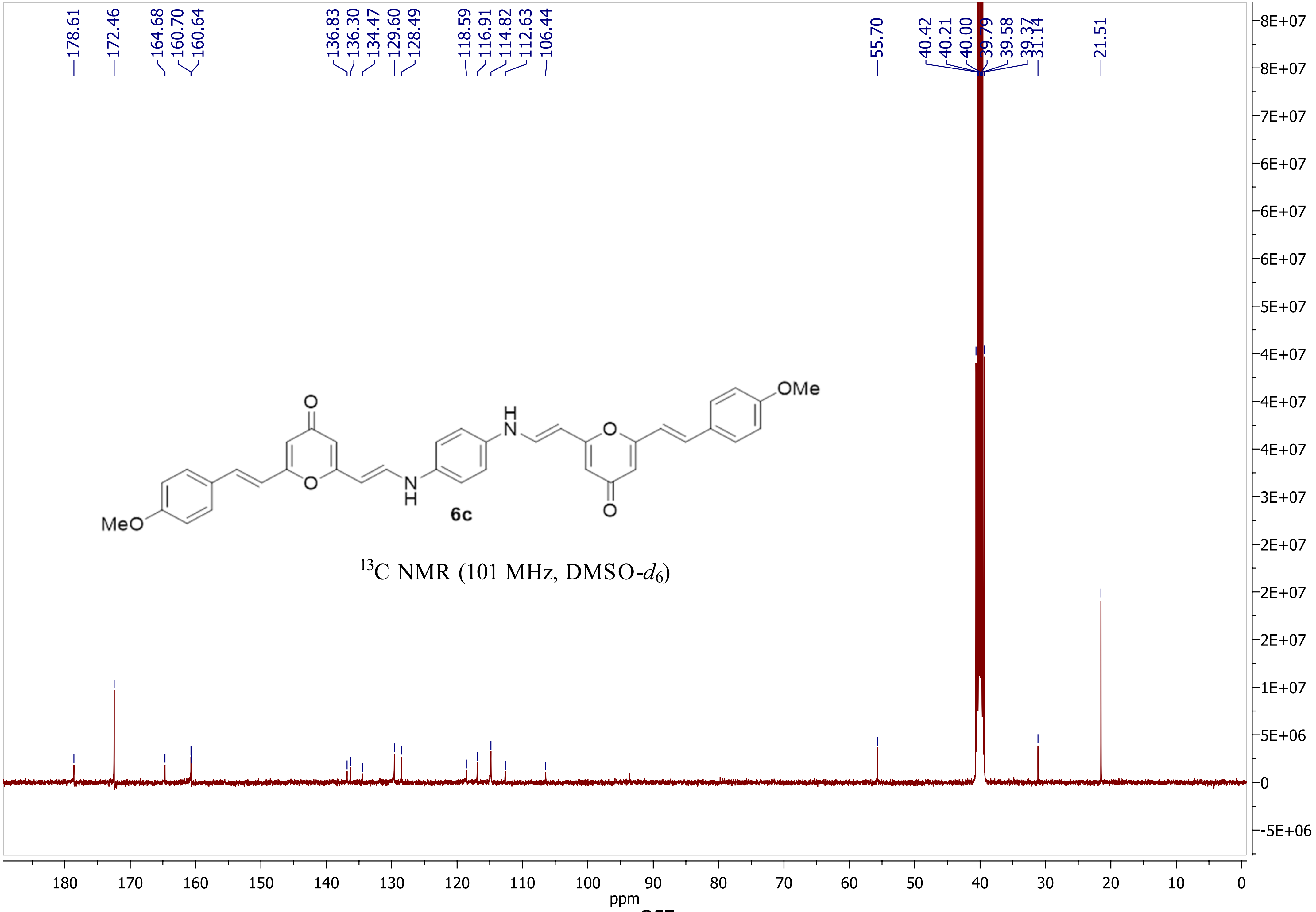
178.61
172.46
164.68
160.70
160.64
136.83
136.30
134.47
129.60
128.49
118.59
116.91
114.82
112.63
106.44
55.70
40.42
40.21
40.00
39.79
39.58
39.37
31.14
21.51
OMe
MeO
6c
13C NMR (101 MHz, DMSO-d6)
ppm

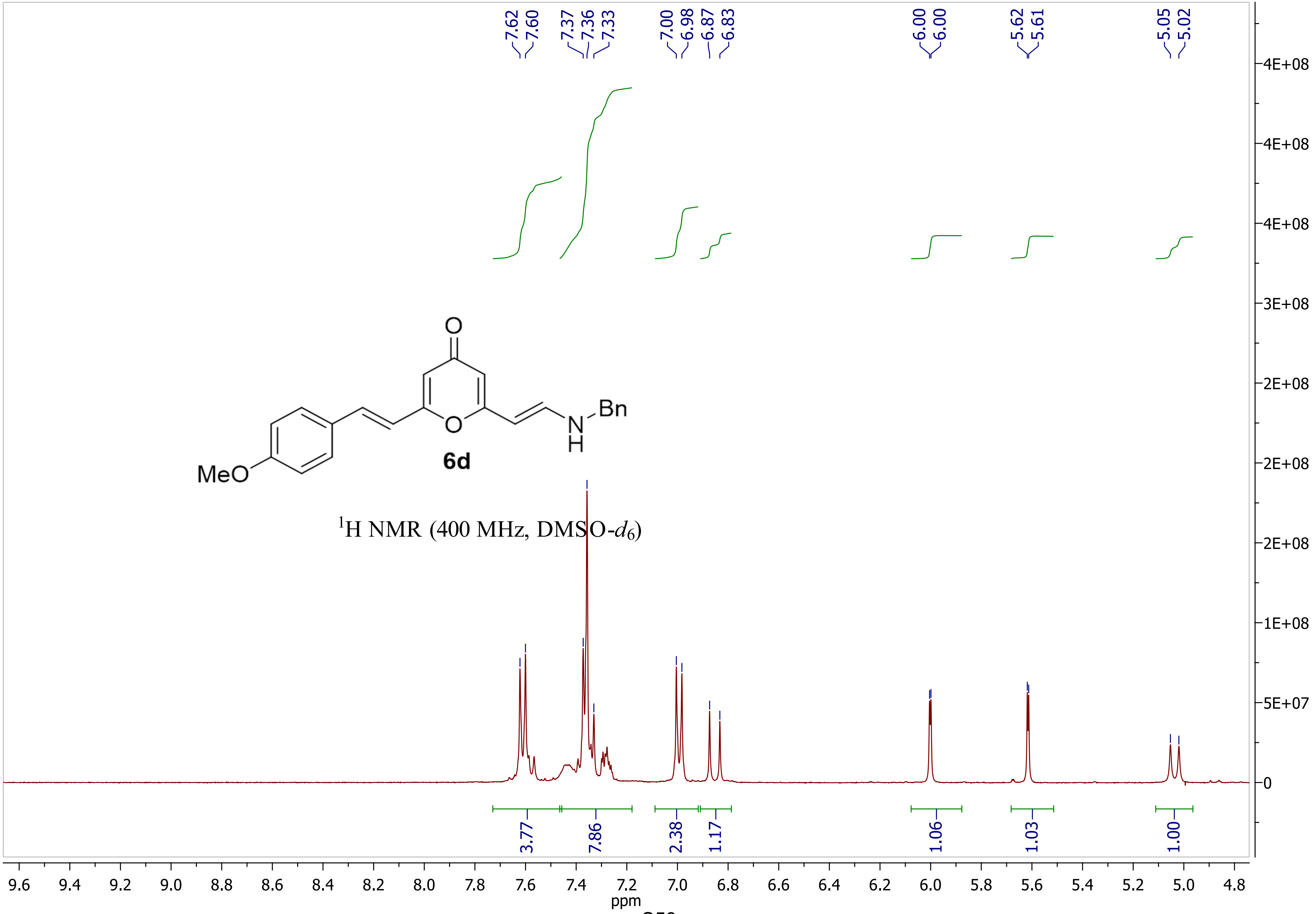
7.62
7.60
7.37
7.36
7.33
7.00
6.98
6.87
6.83
6.00
6.00
5.62
5.61
5.05
5.02
O
O
N
H
Bn
MeO
6d
1H NMR (400 MHz, DMSO-d6)
3.77
7.86
2.38
1.17
1.06
1.03
1.00
ppm

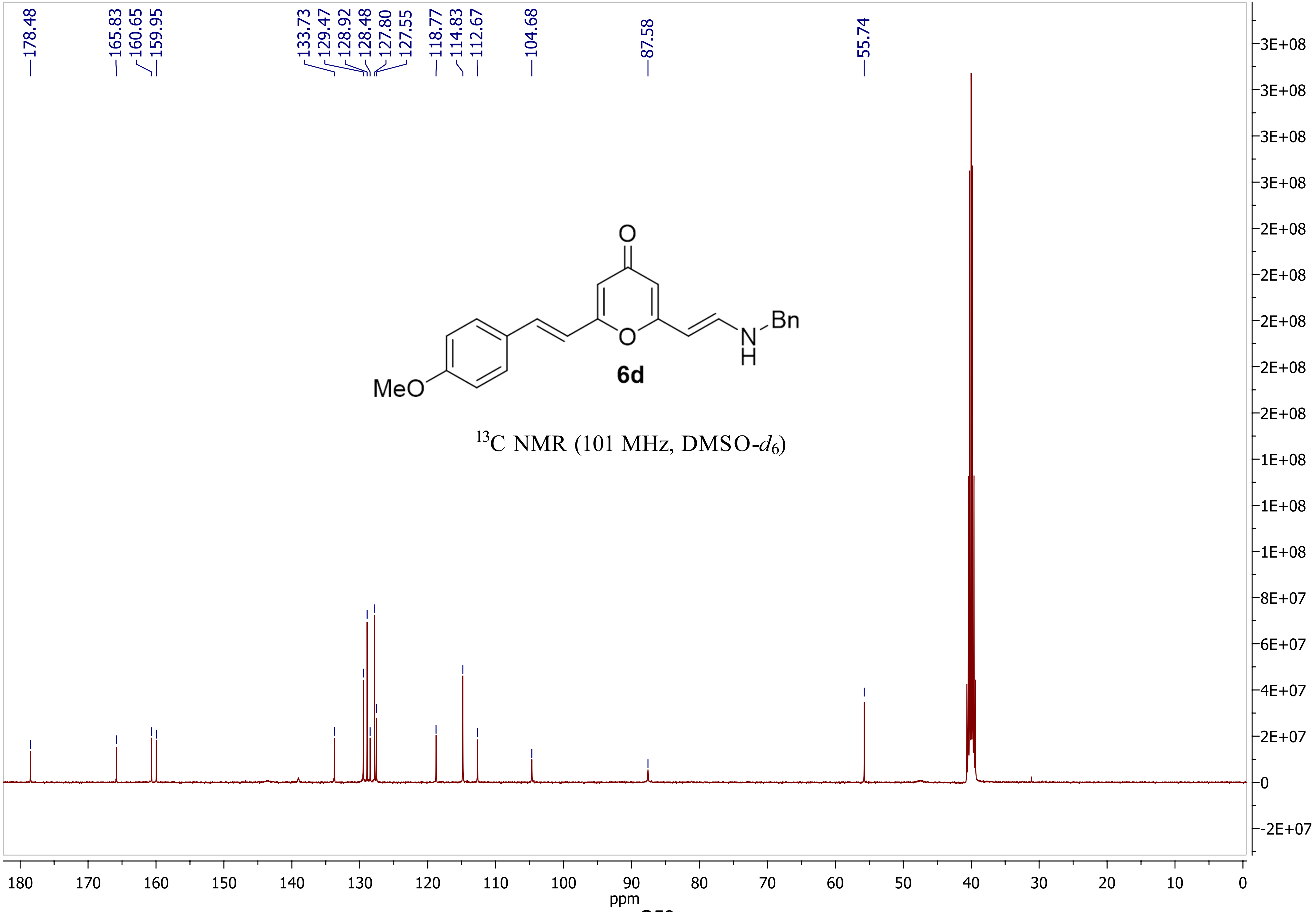
178.48
165.83
160.65
159.95
133.73
129.47
128.92
128.48
127.80
127.55
118.77
114.83
112.67
104.68
87.58
55.74
MeO
O
O
N
H
Bn
6d
^{13}C NMR (101 MHz, DMSO-d_6)
ppm

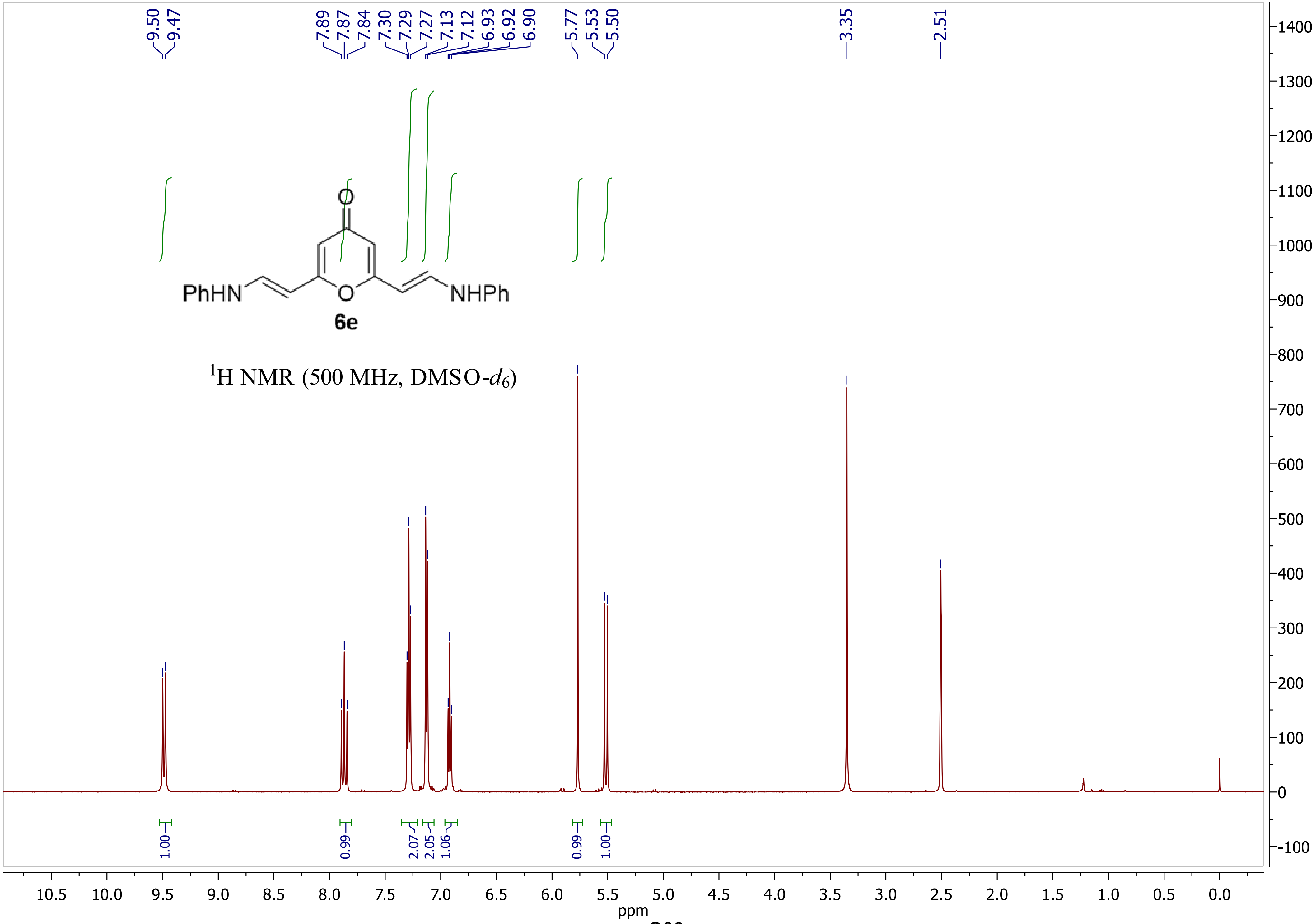
9.50
9.47
7.89
7.87
7.84
7.30
7.29
7.27
7.13
7.12
6.93
6.92
6.90
5.77
5.53
5.50
3.35
2.51
PhHN
O
NHPh
6e
^{1}H NMR (500 MHz, DMSO-d_6)
1.00
0.99
2.07
2.05
1.06
0.99
1.00
ppm

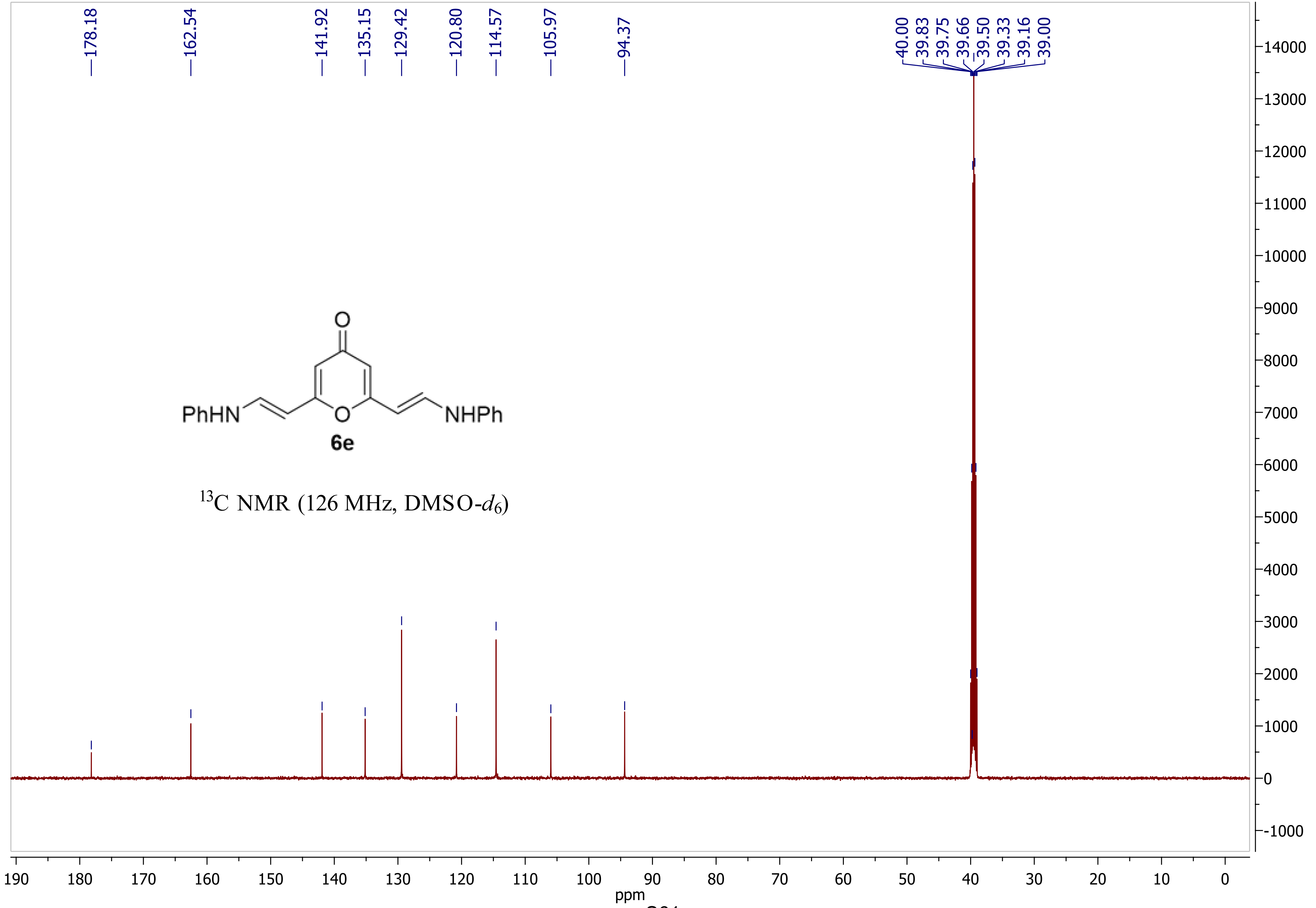
178.18
162.54
141.92
135.15
129.42
120.80
114.57
105.97
94.37
40.00
39.83
39.75
39.66
39.50
39.33
39.16
39.00
PhHN
NHPh
O
O
6e
^{13}C NMR (126 MHz, DMSO-d_6)
ppm

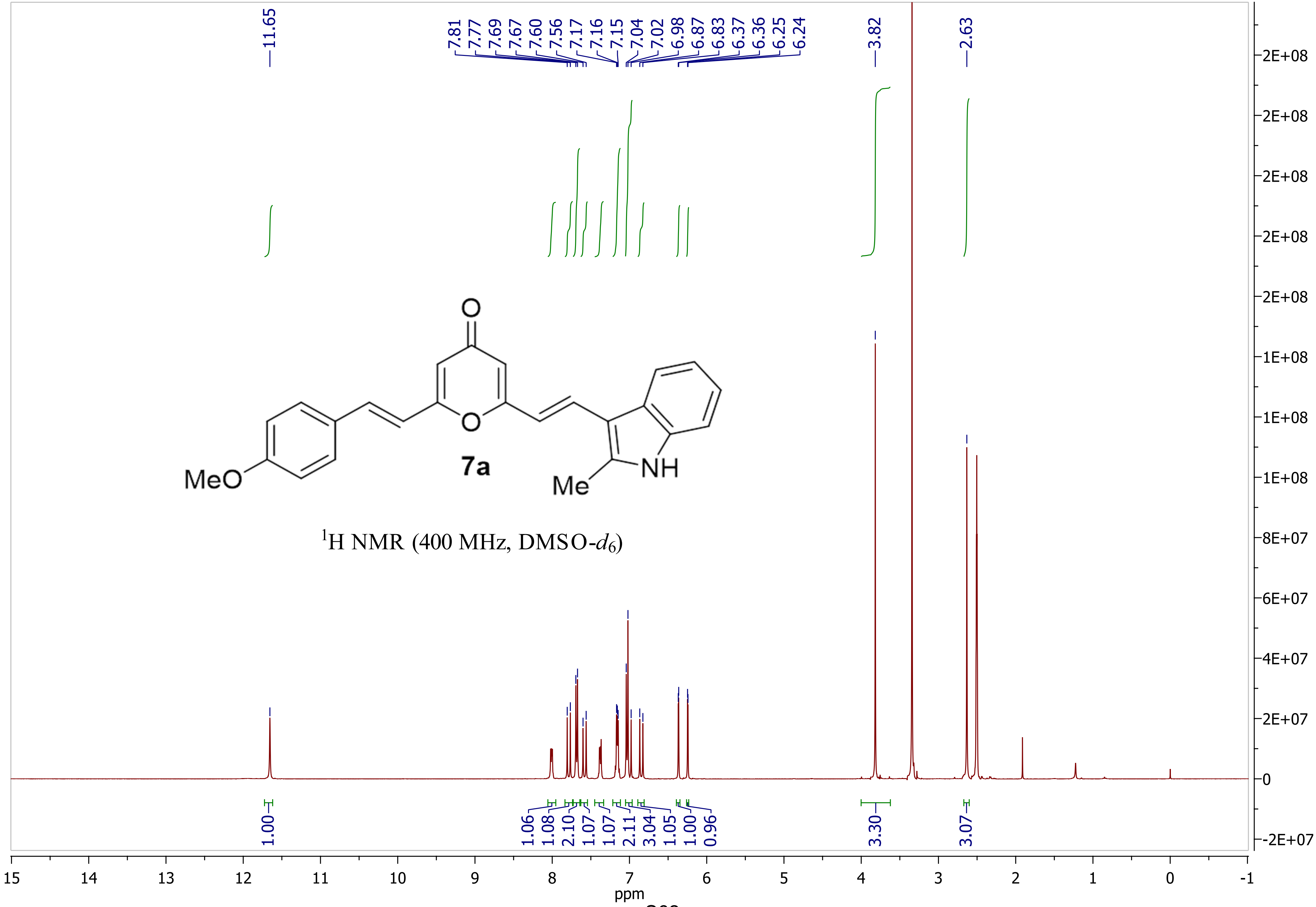

11.65
7.81
7.77
7.69
7.67
7.60
7.56
7.17
7.16
7.15
7.04
7.02
6.98
6.87
6.83
6.37
6.36
6.25
6.24
3.82
2.63
O
O
MeO
7a
Me
NH
1H NMR (400 MHz, DMSO-d6)
1.00
1.06
1.08
2.10
1.07
1.07
2.11
3.04
1.05
1.00
0.96
3.30
3.07
2E+08
1E+08
8E+07
6E+07
4E+07
2E+07
0
-2E+07
15
14
13
12
11
10
9
8
7
6
5
4
3
2
1
0
-1
ppm

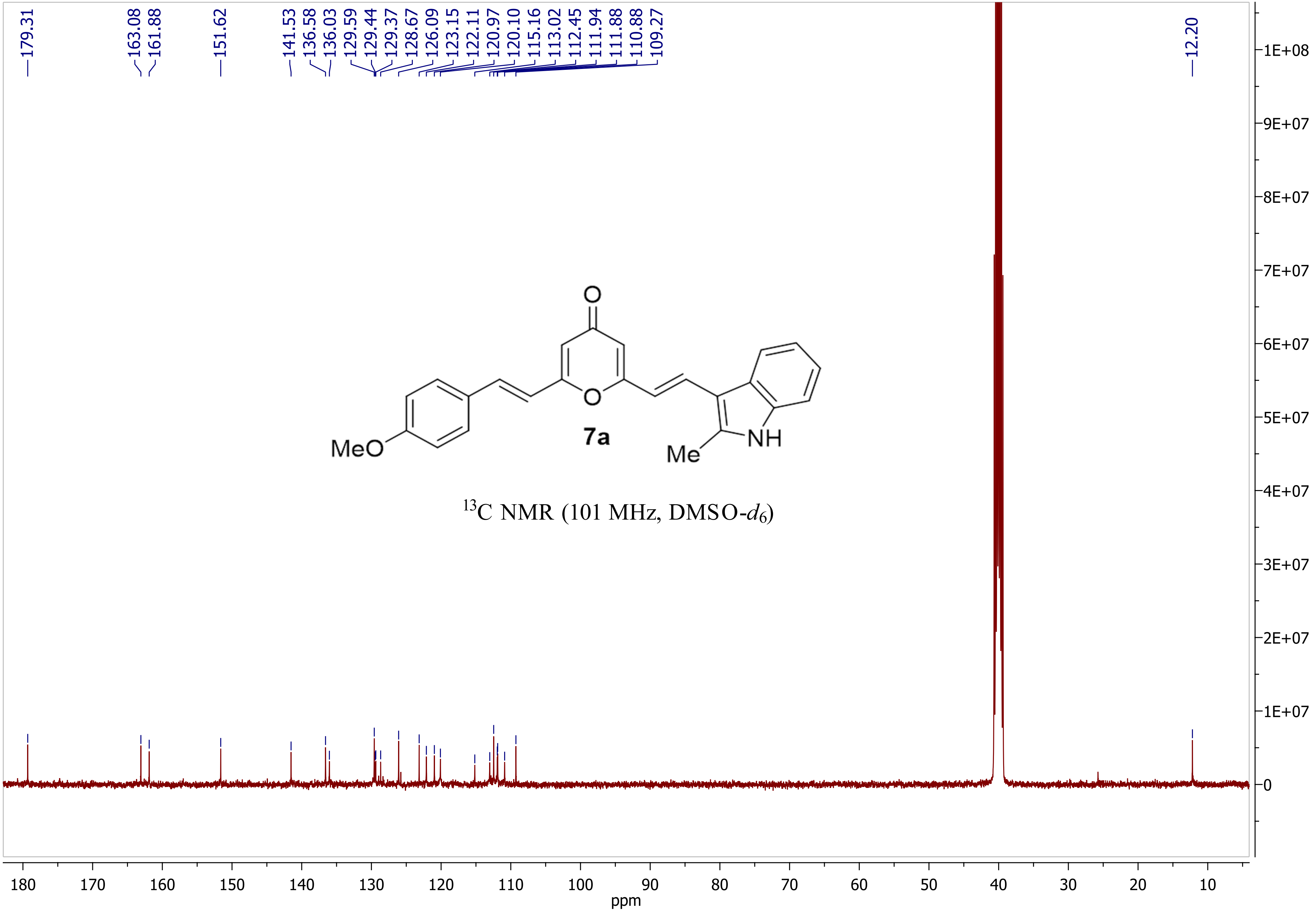

179.31
163.08
161.88
151.62
141.53
136.58
136.03
129.59
129.44
129.37
128.67
126.09
123.15
122.11
120.97
120.10
115.16
113.02
112.45
111.94
111.88
110.88
109.27
12.20
MeO
7a
Me
NH
^{13}C NMR (101 MHz, DMSO-d_6)
1E+08
9E+07
8E+07
7E+07
6E+07
5E+07
4E+07
3E+07
2E+07
1E+07
0
180 170 160 150 140 130 120 110 100 90 80 70 60 50 40 30 20 10
ppm

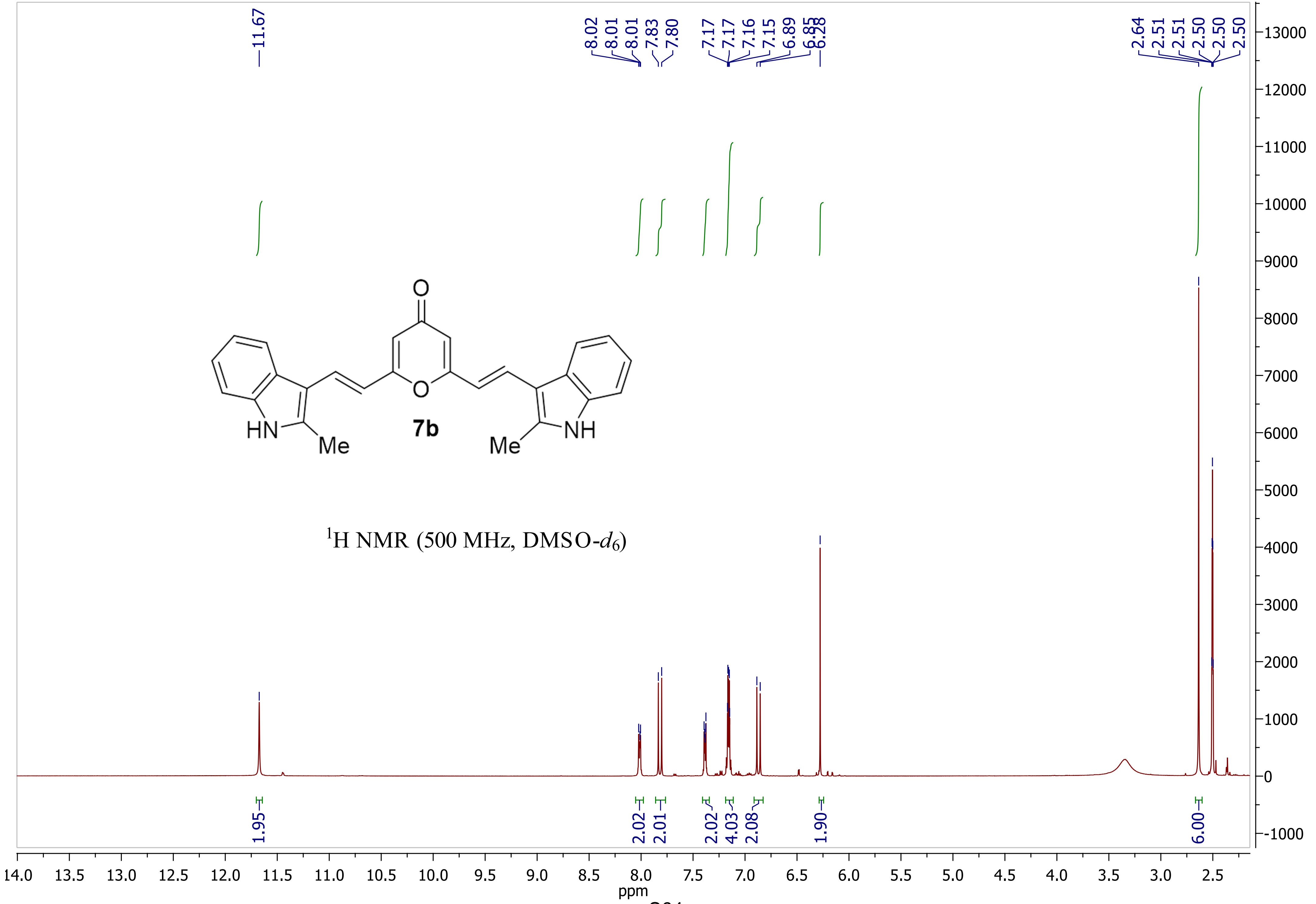
11.67
8.02
8.01
8.01
7.83
7.80
7.17
7.17
7.16
7.15
6.89
6.85
6.28
2.64
2.51
2.51
2.50
2.50
2.50
O
HN
Me
7b
Me
NH
^{1}H NMR (500 MHz, DMSO-d_6)
1.95
2.02
2.01
2.02
4.03
2.08
1.90
6.00
ppm
14.0 13.5 13.0 12.5 12.0 11.5 11.0 10.5 10.0 9.5 9.0 8.5 8.0 7.5 7.0 6.5 6.0 5.5 5.0 4.5 4.0 3.5 3.0 2.5
13000 12000 11000 10000 9000 8000 7000 6000 5000 4000 3000 2000 1000 0 -1000

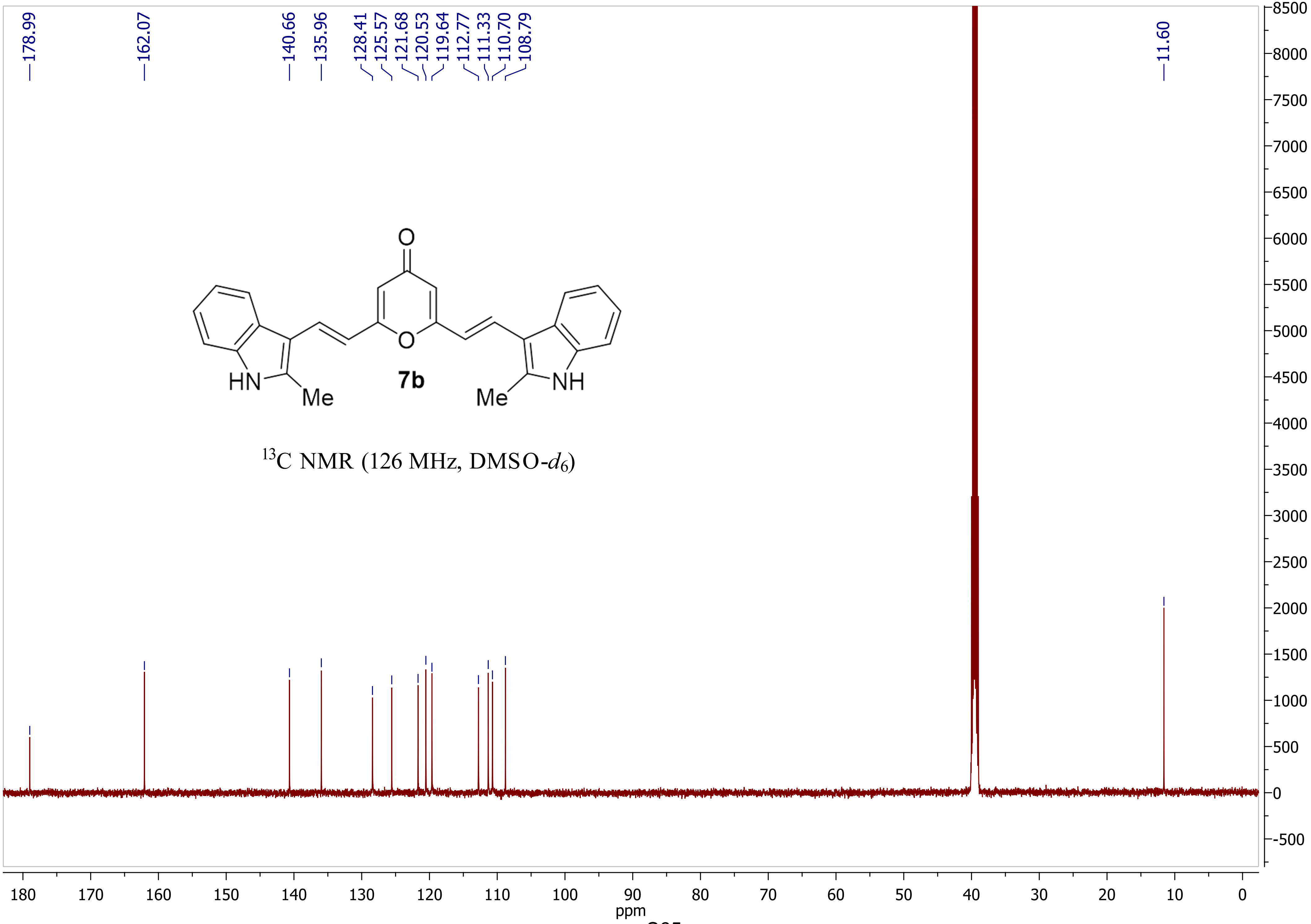
178.99
162.07
140.66
135.96
128.41
125.57
121.68
120.53
119.64
112.77
111.33
110.70
108.79
11.60
O
HN
Me
7b
Me
NH
^{13}C NMR (126 MHz, DMSO-d_6)
ppm

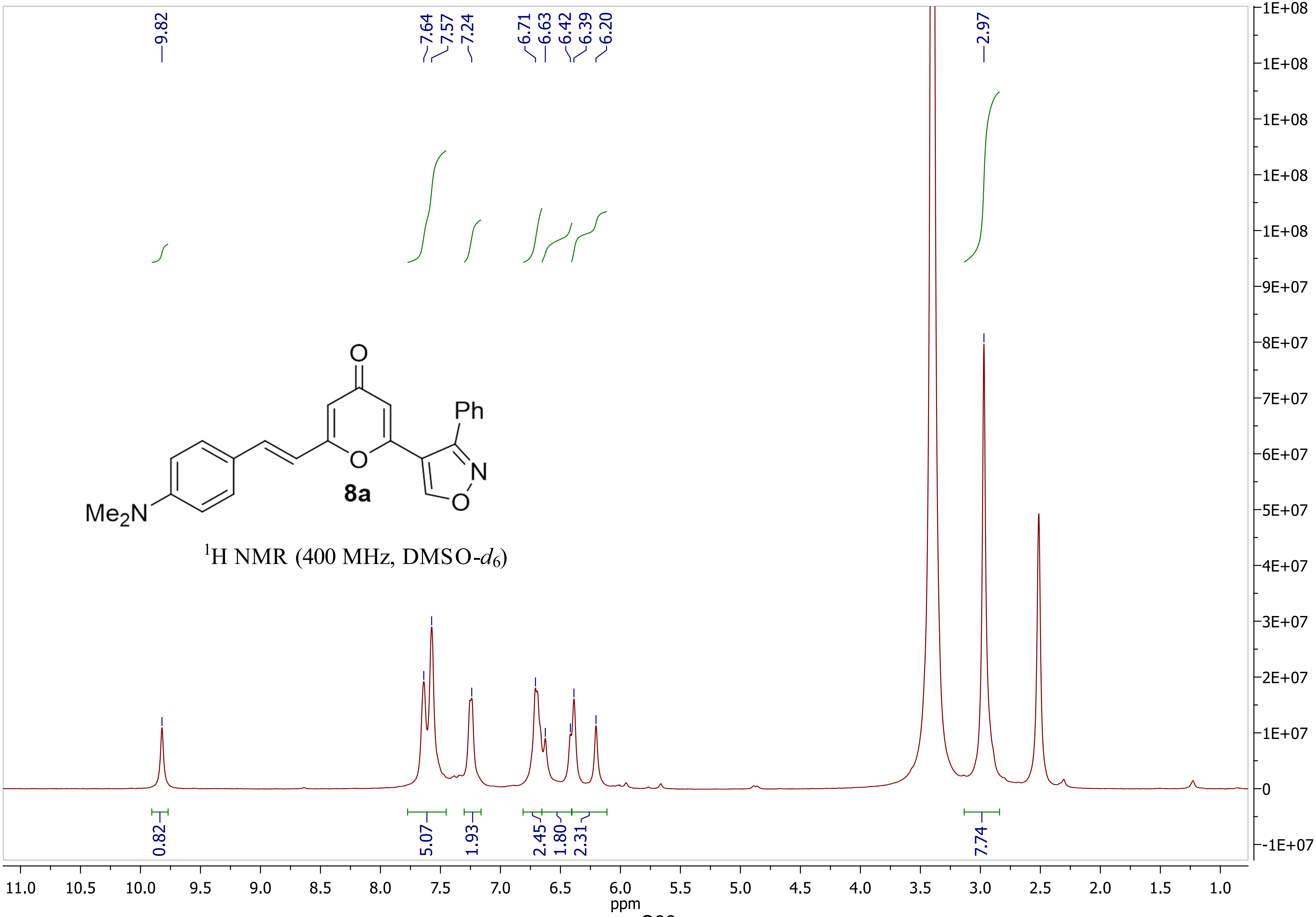

9.82
7.64
7.57
7.24
6.71
6.63
6.42
6.39
6.20
2.97
O
Ph
N
O
O
Me2N
8a
1H NMR (400 MHz, DMSO-d6)
0.82
5.07
1.93
2.45
1.80
2.31
7.74
11.0 10.5 10.0 9.5 9.0 8.5 8.0 7.5 7.0 6.5 6.0 5.5 5.0 4.5 4.0 3.5 3.0 2.5 2.0 1.5 1.0
ppm
1E+08
1E+08
1E+08
1E+08
1E+08
9E+07
8E+07
7E+07
6E+07
5E+07
4E+07
3E+07
2E+07
1E+07
0
-1E+07

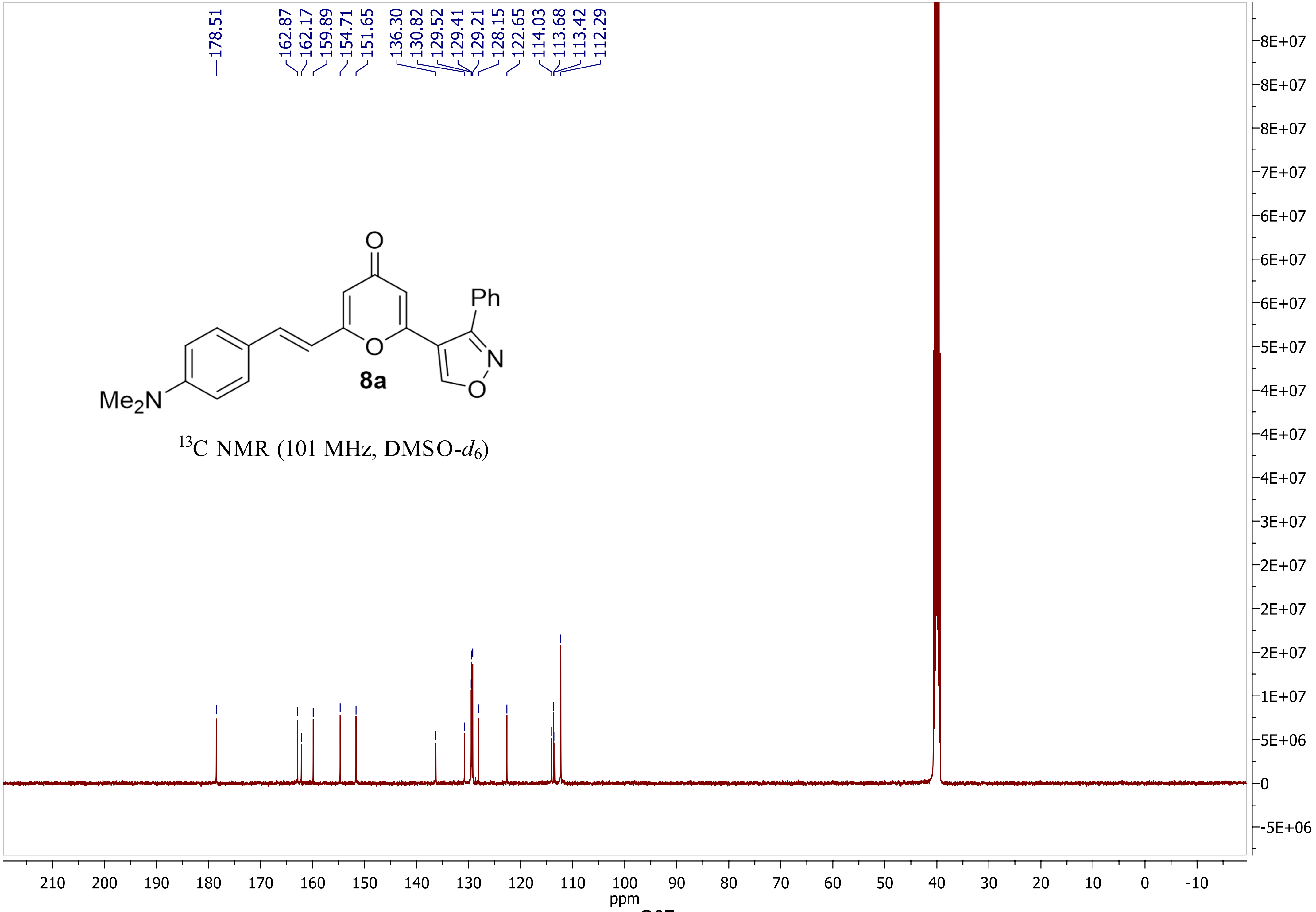
178.51
162.87
162.17
159.89
154.71
151.65
136.30
130.82
129.52
129.41
129.21
128.15
122.65
114.03
113.68
113.42
112.29
O
Ph
N
O
O
Me2N
8a
13C NMR (101 MHz, DMSO-d6)
8E+07
8E+07
8E+07
7E+07
6E+07
6E+07
6E+07
5E+07
4E+07
4E+07
4E+07
3E+07
2E+07
2E+07
2E+07
1E+07
5E+06
0
-5E+06
210 200 190 180 170 160 150 140 130 120 110 100 90 80 70 60 50 40 30 20 10 0 -10
ppm

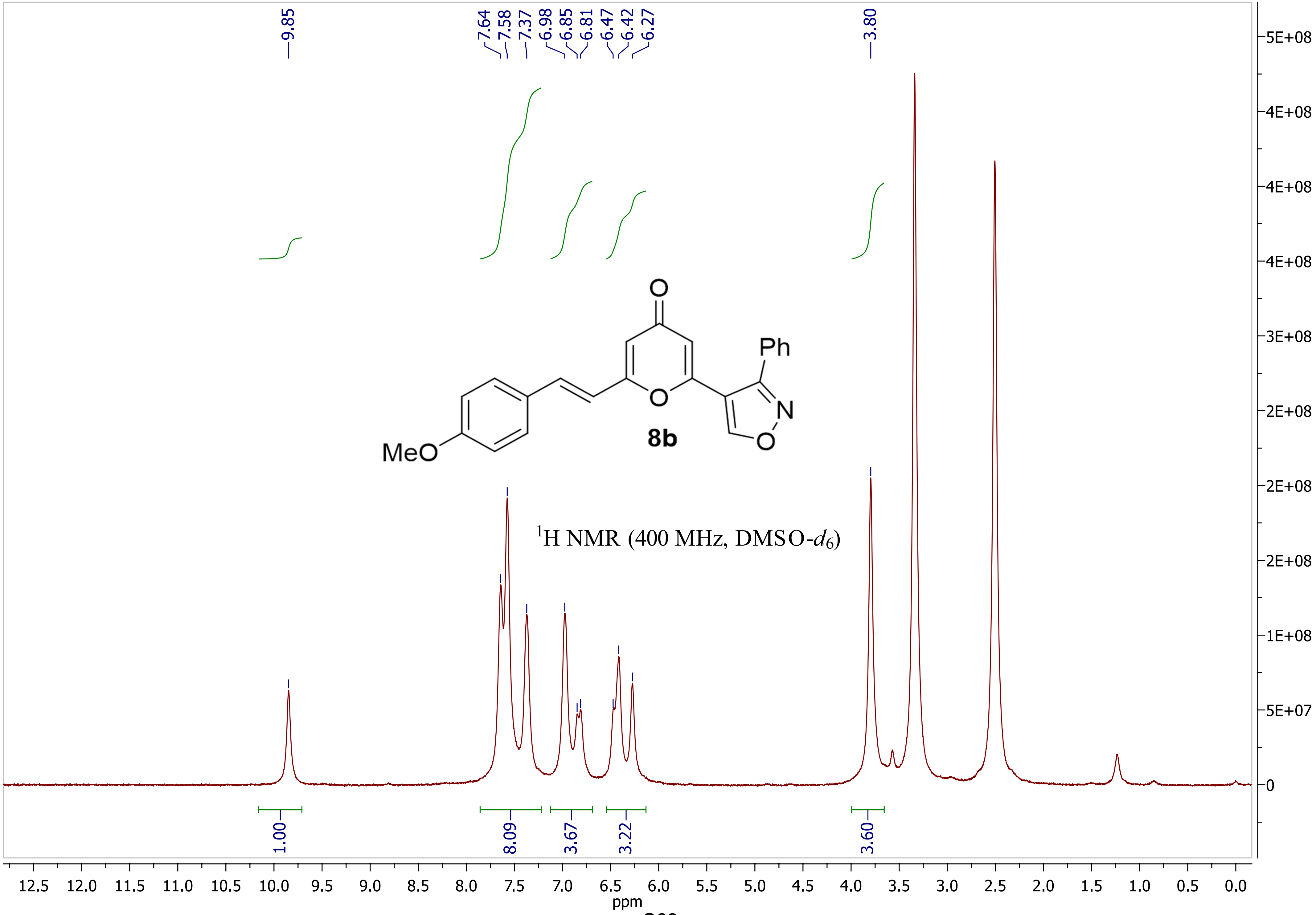

9.85
7.64
7.58
7.37
6.98
6.85
6.81
6.47
6.42
6.27
3.80
O
Ph
N
O
MeO
O
8b
¹H NMR (400 MHz, DMSO-d6)
1.00
8.09
3.67
3.22
3.60
ppm

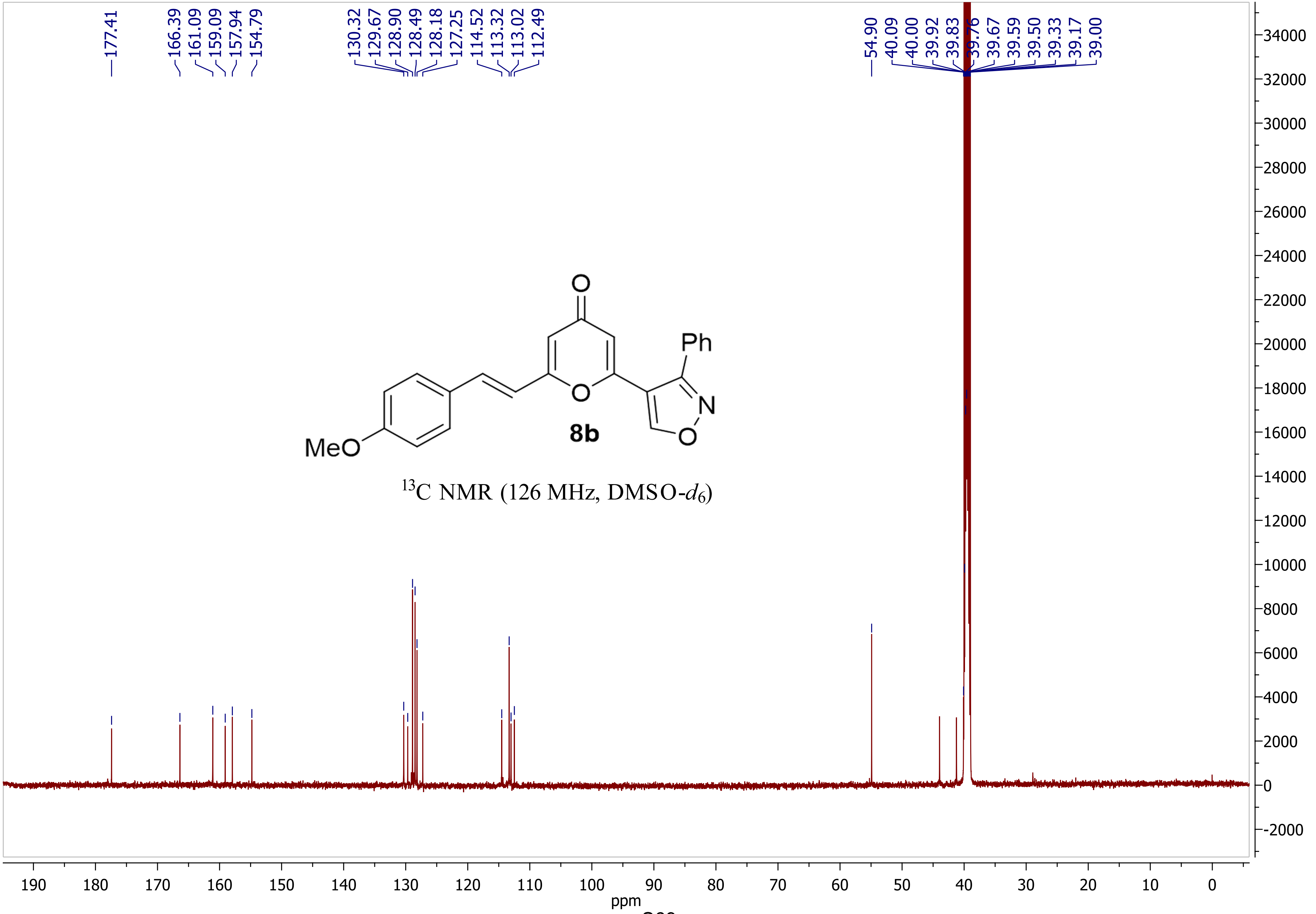
177.41
166.39
161.09
159.09
157.94
154.79
130.32
129.67
128.90
128.49
128.18
127.25
114.52
113.32
113.02
112.49
54.90
40.09
40.00
39.92
39.83
39.76
39.67
39.59
39.50
39.33
39.17
39.00
O
Ph
O
N
O
MeO
8b
^{13}C NMR (126 MHz, DMSO-d_6)
ppm

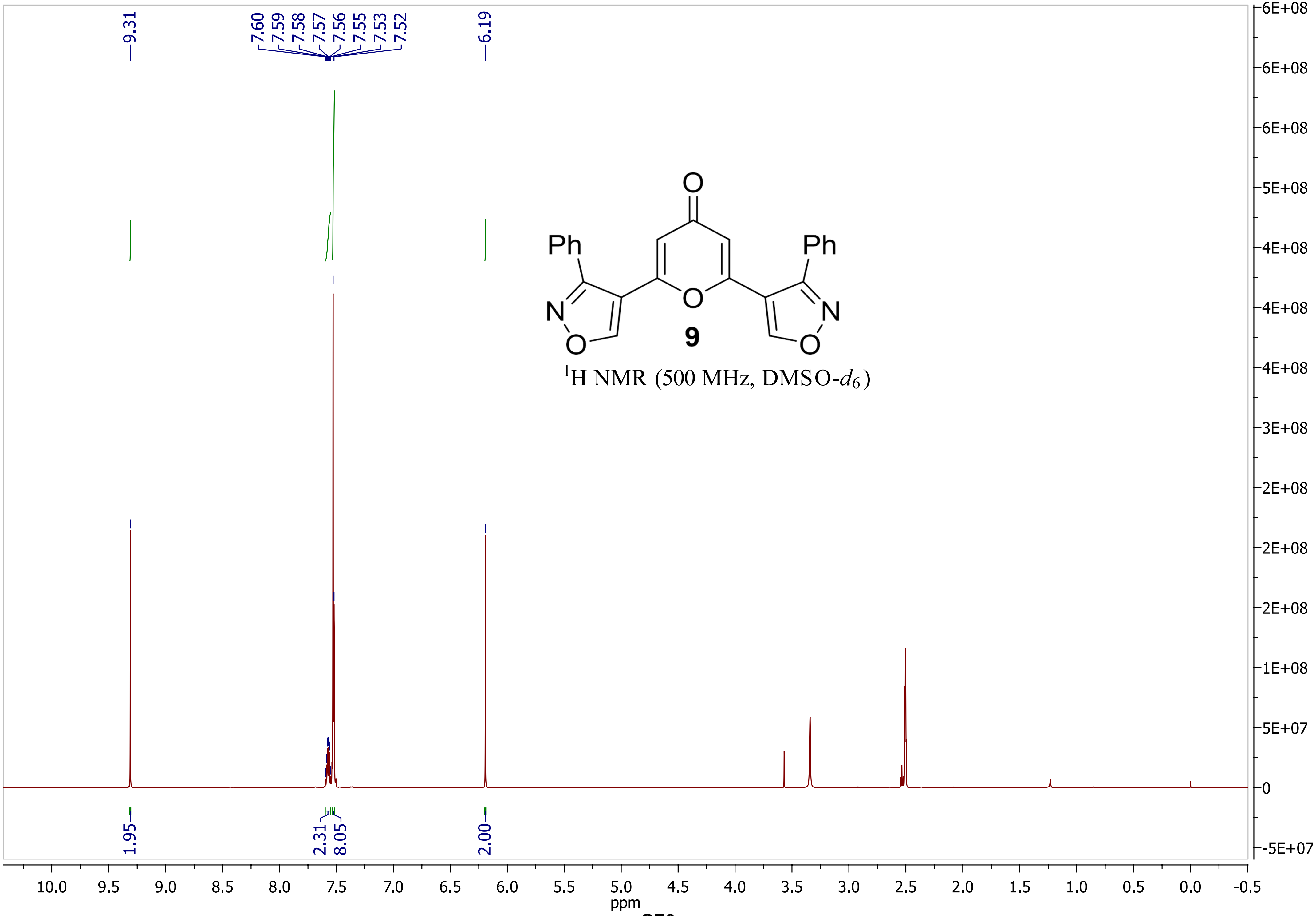
9.31
7.60
7.59
7.58
7.57
7.56
7.55
7.53
7.52
6.19
Ph
Ph
O
O
N
O
N
O
9
¹H NMR (500 MHz, DMSO-d_6)
1.95
2.31
8.05
2.00
ppm

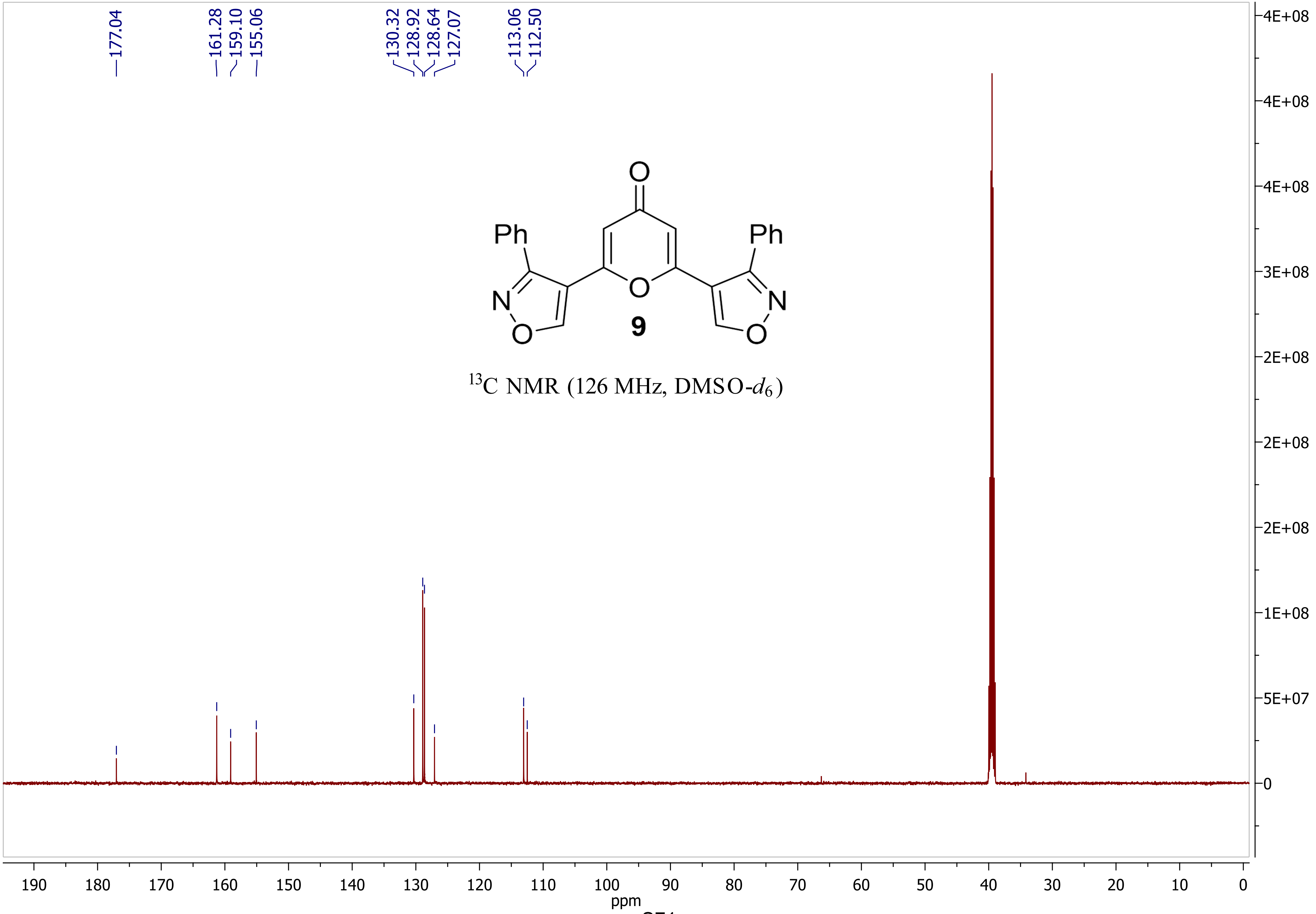
177.04
161.28
159.10
155.06
130.32
128.92
128.64
127.07
113.06
112.50
O
Ph
Ph
N
O
O
N
O
9
^{13}C NMR (126 MHz, DMSO-d_6)
4E+08
4E+08
4E+08
3E+08
2E+08
2E+08
2E+08
1E+08
5E+07
0
190
180
170
160
150
140
130
120
110
100
90
80
70
60
50
40
30
20
10
0
ppm
